# Stability-Indicating RP-HPLC Determination of Acetylsalicylic Acid, Dextromethorphan Hydrobromide, Phenylephrine Bitartrate, and Chlorpheniramine Maleate in an Effervescent Tablet Formulation

**DOI:** 10.1093/jaoacint/qsag070

**Published:** 2026-08-07

**Authors:** Selin Atlı, Özlem Aksu Dönmez

**Affiliations:** Yıldız Technical University, Department of Analytical Chemistry, Faculty of Arts and Sciences, Davutpaşa Mah. Davutpaşa Caddesi, Esenler, Istanbul 34220, Turkey; Yıldız Technical University, Department of Analytical Chemistry, Faculty of Arts and Sciences, Davutpaşa Mah. Davutpaşa Caddesi, Esenler, Istanbul 34220, Turkey

## Abstract

**Background:**

Multicomponent effervescent tablets containing acetylsalicylic acid (ASA), dextromethorphan hydrobromide (DMH), phenylephrine bitartrate (PB), and chlorpheniramine maleate (CPM) pose analytical challenges due to their complex excipient composition and moisture sensitivity. Accurate quantification of these active pharmaceutical ingredients and their related degradation products or impurities is essential for product quality and stability evaluation.

**Objective:**

To develop and validate a stability-indicating reversed-phase HPLC (RP-HPLC) method for the determination of ASA, DMH, PB, and CPM in an effervescent tablet formulation, together with the monitoring of selected related impurities.

**Methods:**

Chromatographic separation was achieved on an InertSustain C8 column (150 × 4.6 mm, 5 µm) using a phosphate buffer–methanol gradient containing sodium heptane sulfonate at pH 2.0, with dual-wavelength detection at 215 and 304 nm. The method was validated in accordance with ICH Q2 (R2) for specificity, linearity, accuracy, precision, and solution stability. Forced degradation studies under acid, base, oxidative, photolytic, and thermal conditions confirmed stability-indicating capability.

**Results:**

The method provided baseline resolution of all analytes within a single chromatographic program. Linearity (R² >0.998 for APIs, >0.993 for impurities), recoveries (98–102% for APIs, 90–110% for impurities), and precision (RSD ≤2% for APIs, ≤5% for impurities) met acceptance criteria.

**Conclusions:**

The developed RP-HPLC method demonstrated reliable performance and can be applied for routine quality control and stability testing of the evaluated effervescent tablet formulation containing multiple active ingredients.

**Highlights:**

This method employs ion-pairing at pH 2 optimized for the effervescent matrix and dual-wavelength detection for the quantification of actives and selected impurities. Fully validated in accordance with ICH Q2 (R2), it demonstrates a clear stability-indicating capability and suitability for QC and stability studies of the evaluated effervescent tablet formulation.

Multicomponent pharmaceutical formulations are widely used in the treatment of cold and cough because they provide simultaneous relief from multiple symptoms. Effervescent tablets combining acetylsalicylic acid (ASA), dextromethorphan hydrobromide (DMH), phenylephrine bitartrate (PB), and chlorpheniramine maleate (CPM) are commonly marketed as over-the-counter remedies (1–3). ASA provides analgesic and antipyretic effects (4), DMH acts as a centrally acting antitussive (5), PB reduces nasal congestion through vasoconstriction (6), and CPM alleviates allergic symptoms via H_1_-receptor antagonism (7). Their inclusion in a single formulation enables simultaneous management of fever, cough, nasal obstruction, and allergy-related discomfort, improving patient compliance.

Effervescent tablets represent a challenging dosage form for analytical quantification due to their high excipient load and reactive nature, which can compromise chromatographic separation. In addition, ASA is susceptible to hydrolytic degradation to salicylic acid (SA) (8), and DMH may contain several related impurities such as A, B, C, and D described in pharmacopeial monographs (9). The accurate quantification of these impurities is critical for ensuring product quality and regulatory compliance. Although impurities B and C were available and analyzed, impurity A could not be procured due to restrictions associated with its narcotic classification, which posed challenges in shipment and supply. Furthermore, impurity D, reported as a chiral enantiomer of DMH, could not be included under the achiral RP-HPLC conditions (10).

Several analytical methods have been reported for the determination of ASA, DMH, PB, and CPM, either alone or in limited combinations. Examples include stability-indicating assays for DMH (11), RP-HPLC methods for DMH–phenylephrine–triprolidine formulations (12), and studies on ASA–paracetamol–caffeine combinations (13) or DMH–phenylephrine–chlorpheniramine preparations (14). However, these methods generally target fewer analytes, simpler dosage forms, or do not comprehensively address related impurities.

To the best of our knowledge, no validated method has been reported for the determination of ASA, DMH, PB, and CPM together with SA, DMH impurity B, and DMH impurity C in an effervescent tablet dosage form. The present work describes the development and validation of a stability-indicating RP-HPLC method capable of quantifying four APIs and three related impurities using a single chromatographic program. The method has been validated according to ICH Q2 (R2) guidelines (15), confirmed through forced degradation studies, and optimized for the effervescent matrix. It is intended to provide a suitable tool for both quality control and stability testing in line with USP, EP, and EMA regulatory expectations. The chemical structures of the active pharmaceutical ingredients and related impurities investigated in this study are presented in Figure 1.

**Figure 1.**
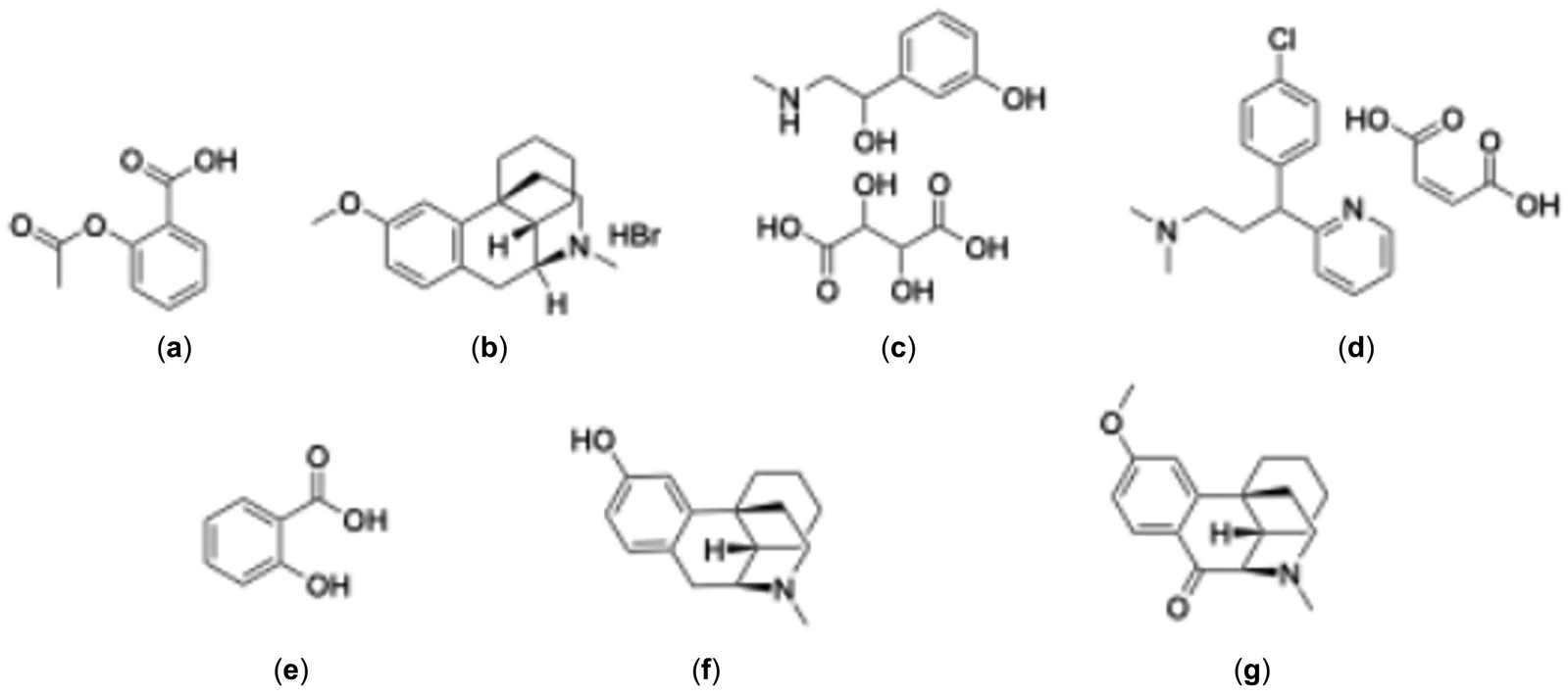
Structural formulas of the active pharmaceutical ingredients and impurities analyzed: (a) ASA; (b) DMH; (c) PB; and (d) CPM; (e) SA; (f) dextromethorphan impurity B; (g) dextromethorphan impurity C.

## Experimental

### Apparatus

- *HPLC.—*HPLC was performed using an Agilent 1260 Infinity HPLC system equipped with a quaternary pump, an autosampler, a column oven, and a photodiode array (PDA) detector. Data acquisition and processing were carried out using Empower 3 chromatography software.
- *Column.—*An InertSustain® C8 (150 mm × 4.6 mm, 5 μm particle size) column was used.

### Reagents

- *Water.*—Ultrapure water was produced using a Milli-Q water purification system.
- *Methanol (MeOH).—*HPLC grade (JT Baker, Phillipsburg, NJ, USA).
- *Orthophosphoric acid (85%).*—ACS grade (SRL, Mumbai, India).
- *Sodium dihydrogen phosphate monohydrate (NaH_2_PO_4_.H_2_O).*—ACS grade (Merck, Darmstadt, Germany).
- *Sodium heptane sulfonate (C_7_H_15_SO_3_Na).*—ACS grade (SRL, Mumbai, India).
- *Sodium hydroxide (NaOH)*.—ACS grade (ChemLab, Zedelgem, Belgium).
- *Hydrochloric acid (HCl).*—ACS grade (IsoLab, Eschau, Germany).
- *Hydrogen peroxide (H_2_O_2_, 30%, w/w).*—ACS grade (Merck, Darmstadt, Germany).

### Reference Standards

- *Acetylsalicylic acid (ASA*, *≥99%).*—(Novacyl, Paris, France).
- *Salicylic acid (SA, ≥99%)*.—(Merck, Darmstadt, Germany).
- *Dextromethorphan hydrobromide monohydrate (DMH, ≥99%)*.—(Divis Laboratories, Hyderabad, India).
- *DMH impurity B, ≥98%.*—(Divis Laboratories, Hyderabad, India).
- *DMH impurity C, ≥95%*.—(Divis Laboratories, Hyderabad, India).
- *Phenylephrine bitartrate (PB, ≥99%)*.—(Malladi Drugs and Pharmaceuticals, Chennai, India).
- *Chlorpheniramine maleate (CPM, ≥98%)*.—(Supriya Lifescience, Mumbai, India).

All reference standards were obtained from commercial suppliers and were used as working standards for the purposes of this study.

Certified impurity standards of DMH impurity B and DMH impurity C were obtained from a commercial supplier (Divis Laboratories, Hyderabad, India). These impurities correspond to those described in the European Pharmacopoeia monograph for dextromethorphan hydrobromide. The chemical names of the impurities are as follows: DMH impurity B (ent-17-methylmorphinan-3-ol) and DMH impurity C (ent-3-methoxy-17-methylmorphinan-10-one). The identity and purity of these standards were confirmed by the supplier certificates of analysis.

### Samples

Effervescent tablets (Alka-Seltzer® Plus Cold & Cough PowerFast Fizz™, citrus flavor), manufactured by Bayer, containing ASA (325 mg), DMH (10 mg), PB (7.8 mg), and CPM (2 mg) per tablet, were purchased from the United States commercial market. According to the product label, the formulation also contained anhydrous citric acid, calcium silicate, dimethicone, flavoring agents, mannitol, potassium bicarbonate, povidone, sodium bicarbonate, and sucralose as inactive ingredients. All samples were stored in their original packaging under controlled temperature and humidity conditions until analysis. All analyses were performed using a single commercially available product from one production batch.

### HPLC Analysis

Chromatographic analysis was performed as summarized in Table 1. Separation was achieved on a C8 column at 45°C using a phosphate buffer with sodium heptane sulfonate (pH 2.0) and methanol under gradient elution (1.5–2 mL/min). Detection was carried out at 215 nm for all analytes except DMH impurity C (304 nm). Injection volume was 50 µL, and tray temperature was 5°C.

**Table 1.** Chromatographic conditions of the HPLC method

Method parameter	Value
Mobile phase A	Phosphate buffer (pH 2.0) containing sodium heptane sulfonate
Mobile phase B	MeOH
Diluent	MeOH + H_2_O (5:95, v/v)
Column	InertSustain C8, 150 × 4.6 mm, 5 µm
Column temperature	45°C
Tray temperature	5°C
Detector	215 nm, 304 nm
Injection volume	50 µL

Mobile phase program	Time, min	Mobile phase A, %	Mobile phase B, %	Flow rate, mL/min
	0.00	80	20	1.5
	4.00	80	20	1.5
	5.00	77	23	1.5
	17.00	77	23	1.5
	18.00	71	29	2.0
	55.00	71	29	2.0
	55.01	80	20	1.5
	59.00	80	20	1.5

### Preparation of Solutions

pH 2 Phosphate Buffer: The buffer was prepared by accurately weighing 1.38 g of sodium dihydrogen phosphate monohydrate and 0.6 g of sodium heptane sulfonate, which were transferred into a 1000 mL volumetric flask containing purified water. The solution was mixed until complete dissolution, and the pH was adjusted to 2 using orthophosphoric acid. The buffer solution was then filtered through a 0.45 µm membrane filter and degassed before use.

### Standard Solutions

Two separate stock standard solutions were prepared to ensure accurate quantification of all active pharmaceutical ingredients.

*Stock Standard Solution 1 (ASA)* was prepared by accurately weighing 65 mg of ASA working standard into a 100 mL volumetric flask. The standard was dissolved in a small volume of diluent using ultrasonic agitation for 5 min and diluted to volume with the same diluent to obtain a final concentration of 650 µg/mL ASA.

*Stock Standard Solution 2 (CPM, DMH, and PB)* was prepared by accurately weighing 10 mg of CPM, 50 mg of dextromethorphan hydrobromide, and 39 mg of PB working standards into a 50 mL volumetric flask. The components were dissolved in diluent with ultrasonic agitation for 5 min and diluted to volume, yielding final concentrations of 200 µg/mL CPM, 1000 µg/mL DMH, and 780 µg/mL PB.

The assay standard solution was prepared by transferring 2.5 mL of Stock Standard Solution 1 and 5 mL of Stock Standard Solution 2 into a 50 mL volumetric flask, followed by dilution to volume with diluent and mixing. The final assay concentrations were 32.5 µg/mL ASA, 100 µg/mL DMH, 20 µg/mL CPM, and 78 µg/mL PB. The solution was filtered through a 0.45 µm nylon membrane filter before injection.

### Impurity Standard Solutions

Stock SA solution was prepared by accurately weighing 5.2 mg of SA working standard into a 100 mL volumetric flask. The standard was dissolved in a small volume of diluent using ultrasonic agitation for 5 min and diluted to volume with the same diluent to obtain a final concentration of 52 µg/mL SA.

Stock Impurity Solution (DMH impurity B and C) was prepared by accurately weighing 2.5 mg of dextromethorphan impurity B and 2.5 mg of dextromethorphan impurity C working standards into a 200 mL volumetric flask. The standards were dissolved in a small volume of water–methanol (50:50, v/v) and diluted to volume with the same solvent mixture to obtain final concentrations of 12.5 µg/mL DMH impurity B and 12.5 µg/mL DMH impurity C.

The impurity standard solution was prepared by transferring 2 mL of the Stock Impurity Solution into a 50 mL volumetric flask, followed by the addition of 2.5 mL of the SA stock solution. The mixture was diluted to volume with diluent and mixed thoroughly. The final concentrations were 0.50 µg/mL DMH impurity B, 0.50 µg/mL DMH impurity C, and 2.6 µg/mL SA. The solution was filtered through a 0.45 µm nylon membrane filter before injection.

### Sample Solutions

Sample Solution 1 (CPM, DMH, and PB) was prepared by accurately weighing 770 mg (equivalent to one-fourth of a tablet) of powdered tablets into a 25 mL volumetric flask. Approximately 15 mL of diluent was added, and the sample was dissolved by mixing. The solution was then diluted to volume with the same diluent and mixed thoroughly. The solution was filtered through a 0.45 µm nylon membrane filter before vialing. The resulting concentrations were 100 µg/mL DMH, 20 µg/mL CPM, and 78 µg/mL PB.

Sample Solution 2 (ASA and SA) was prepared by transferring 1 mL of Sample Solution 1 into a 100 mL volumetric flask and diluting to volume with diluent. The solution was filtered through a 0.45 µm nylon membrane filter before vialing to obtain a final concentration of 32.5 µg/mL ASA. This solution was prepared freshly and injected immediately to minimize ASA degradation to SA.

### Calculations

Calculations for assay determinations were performed by comparing the peak areas of analytes in the sample solutions with those obtained from the corresponding assay standard solutions. Final assay results were calculated as mg/tablet using the peak area ratio together with standard weight, standard potency, dilution factors, sample weight, and average tablet weight.

The concentrations reported throughout the validation studies (linearity, accuracy, precision, LOD, and LOQ) represent the concentrations of the analytical solutions injected into the HPLC system and were therefore expressed in µg/mL.

The assay results for PB, ASA, CPM, and dextromethorphan hydrobromide (DMH) were calculated using the general Equation 1 below:


(1) $$\mathrm{Assay}\;(\mathrm{mg}/\mathrm{tablet})=\;\frac{At}{As}\times \frac{Ws}{50}\times \frac{5}{50}\times \frac{P}{100}\times \frac{25}{Wt}\times \mathrm{Wavg}$$

For ASA, an additional 100-fold dilution step was applied during sample preparation and was included in the calculation factor Equation 2:


(2) $$\mathrm{ASA}\;(\mathrm{mg}/\mathrm{tablet})=\;\frac{At}{As}\times \frac{Ws}{50}\times \frac{2.5}{50}\times \frac{P}{100}\times \frac{25}{Wt}\times \frac{1}{100}\times \mathrm{Wavg}$$

where At and As represent the peak area of the corresponding analyte in the sample solution and the average peak area of the assay standard solution, respectively; Ws is the weight of the corresponding working standard (mg); P is the potency of the corresponding standard (%); Wt is the sample weight used for analysis (mg); and $W_{\mathrm{avg}}$ is the average tablet weight (mg).

Calculations for impurity determinations were performed by comparing the peak areas of impurity peaks in the sample solutions with those obtained from the corresponding impurity standard solutions. The impurity contents were calculated as percentage relative to the labeled amount of the corresponding active pharmaceutical ingredient using the peak area ratio together with standard weight, standard potency, dilution factors, sample weight, and average tablet weight.

The impurity calculations for SA, dextromethorphan impurity B, and dextromethorphan impurity C were performed using Equations 3–4, respectively.


(3) $$\mathrm{SA}\;(\%)\;=\;\frac{At}{As}\times \frac{Ws}{100}\times \frac{2.5}{50}\times \frac{P}{100}\times \frac{25}{Wt}\times \frac{1}{100}\times \frac{\mathrm{Wavg}}{325}\times 100$$

(4) $$\mathrm{DMH}\;\mathrm{Impurity}\;B/C\;(\%)\;=\;\frac{At}{As}\times \frac{Ws}{200}\times \frac{2}{50}\times \frac{P}{100}\times \frac{25}{Wt}\times \frac{\mathrm{Wavg}}{10}\times 100$$

where At and As represent the peak area of the corresponding impurity in the sample solution and the average peak area of the impurity standard solution, respectively; Ws is the weight of the corresponding impurity standard (mg); P is the potency of the impurity standard (%); Wt is the sample weight used for analysis (mg); and $W_{\mathrm{avg}}$ is the average tablet weight (mg).

The recovery of each concentration level for accuracy (Tables 3 and 4) was determined using Equation 5:


(5) $$\mathrm{Recovery}\;(\%)=\;\frac{\mathrm{Experimental}\;\mathrm{concentration}}{\mathrm{Prepared}\;\mathrm{concentration}}\times 100$$

## Results

### Method Development and Chromatographic Considerations

Initial chromatographic experiments using a phosphate buffer–methanol system at pH 3 under gradient elution demonstrated that, although all analytes were detectable, the resolution between ASA and its primary degradation product, SA, was insufficient. The strong dependence of retention behavior on mobile phase pH resulted in pronounced selectivity changes even with minor pH variations, indicating that strict pH control was essential to achieve adequate separation.

Further evaluation revealed partial coelution of dextromethorphan hydrobromide (DMH)–related impurities and interference from the effervescent tablet matrix. Optimization of the diluent composition showed that the use of 5% methanol in water minimized peak distortion at higher injection volumes, which was necessary to ensure sufficient sensitivity for impurity determination. To further improve chromatographic performance, sodium heptane sulfonate was introduced as an ion-pairing reagent at pH 2. This approach markedly enhanced the separation of basic analytes, particularly DMH and its related impurities, while reducing excipient-related interference arising from the effervescent formulation.

The final chromatographic conditions, including the use of an InertSustain C8 column, ion-pairing at pH 2.0, gradient elution with methanol, elevated column temperature, and dual-wavelength detection, collectively enabled complete baseline separation of all active pharmaceutical ingredients and related impurities within a single chromatographic program. The selection of a C8 stationary phase, in combination with ion-pairing, provided improved selectivity and reduced peak tailing compared with more conventional approaches, contributing to more stable and reliable peak shapes during routine analysis.

Compared with previously reported HPLC methods for multi-ingredient cold and cough preparations, which generally focused on fewer active components and did not comprehensively address impurity profiling, the present method allows the determination of four active pharmaceutical ingredients together with degradation and some selected related impurities using a single chromatographic program. This approach was specifically optimized to address the analytical challenges associated with the evaluated effervescent tablet formulation and was subsequently validated in accordance with ICH Q2 (R2) requirements for its intended application in quality control and stability testing.

### Method Validation

- *Specificity.—*Specificity was demonstrated by injecting diluent, placebo solutions, assay and impurity standard solutions, individual component solutions at their specification levels, and sample solutions with and without spiking under the optimized chromatographic conditions. As shown in Figure 2, the assay standard chromatogram confirmed clear separation of ASA, DMH, PB, and CPM, whereas Figures 3 and 4 illustrate the distinct peaks of SA, DMH impurity B, and DMH impurity C in the impurity standards. Chromatograms of sample solutions at 215 nm and 304 nm (Figure 5) showed baseline resolution of all analytes and related impurities with no coeluting or interfering peaks. Placebo chromatograms confirmed matrix selectivity, as no peaks were detected at the retention times of the analytes or impurities. Peak purity results, evaluated using Empower 3 software, demonstrated that the purity angle was lower than the purity threshold for each analyte and impurity peak, confirming peak homogeneity.
- *Linearity of response*.—Linearity was evaluated for both assay and impurity determinations across a broad concentration range. For assay, detector response linearity of ASA, PB, CPM, and DMH was assessed using six concentration levels covering 25–150% of the target concentration. The evaluated concentration ranges were 8.13–48.75 µg/mL for ASA, 5.0–30 µg/mL for CPM, 25.0–150 µg/mL for DMH, and 19.5–117 µg/mL for PB. As shown in Table 2 and illustrated in Figure 6, all assay analytes demonstrated excellent linearity, with correlation coefficients (r) ≥ 0.998 and y-intercepts not exceeding 1.2%, confirming a strong linear relationship between peak area and concentration.
  For impurity determination, linearity was established from the LOQ to 150% of the specification level for SA, DMH impurity B, and DMH Impurity C. The evaluated concentration ranges were 0.13–3.90 µg/mL for SA, 0.35–0.75 µg/mL for DMH impurity B, and 0.20–0.75 µg/mL for DMH impurity C. Eleven levels were prepared for SA (5–150%), six levels for DMH impurity B (70–150%), and eight levels for DMH impurity C (40–150%). As presented in Table 2, all impurities exhibited correlation coefficients (r) > 0.993, with y-intercepts below 1.9%, supporting reliable quantification across the studied ranges.
- *Accuracy.*—Accuracy was evaluated for both assay and impurity determinations across three concentration levels. For assay, triplicate preparations at 80, 100, and 120% of the nominal concentration were analyzed for ASA, PB, CPM, and DMH. As presented in Table 3, mean recoveries ranged from 98.57 to 101.68%, with RSD values ≤1.52%, meeting the acceptance criteria of 98–102% and RSD ≤2%.
  For impurities, accuracy was assessed at the LOQ, 100%, and 120% levels for SA, DMH impurity B, and DMH impurity C. LOQ samples were spiked into placebo, while the 100 and 120% levels were spiked into sample solutions. As shown in Table 4, recoveries ranged between 94.48 and 106.81%, with mean values of 100.64% for SA, 101.61% for DMH impurity B, and 105.50% for DMH impurity C. Precision at each level complied with acceptance criteria (RSD ≤4.80%).
  The reported concentrations in Tables 3 and 4 represent the concentrations of the analytical solutions injected into the HPLC system and were therefore expressed in µg/mL.
- *System suitability*.—System suitability was evaluated for both assay and impurity determinations to confirm adequate chromatographic performance. For assay, five consecutive injections of the standard solution met all predefined acceptance criteria, with RSD of peak areas ≤2%, theoretical plates ≥1500, and tailing factors ≤2.0. For assay analytes PB, ASA, CPM, and dextromethorphan hydrobromide (DMH), the RSD of replicate injections was ≤0.47%, indicating excellent precision. Theoretical plate counts ranged from 1796 (PB) to 9689 (CPM), and tailing factors were between 1.13 and 1.30, all within acceptable limits.
  For impurities, three replicate injections of the impurity standard solution also fulfilled suitability criteria, with RSD ≤5%, theoretical plates ≥2000, and tailing factors ≤2.0. RSD values were ≤0.13% for SA and DMH impurity C, and 3.19% for DMH impurity B. Theoretical plate numbers were 2937 (SA) to 13 575 (DMH impurity B), and tailing factors ranged between 1.09 and 1.23.
- *Precision*.—Precision was evaluated for both assay and impurity determinations in terms of repeatability and intermediate precision. Repeatability was assessed using six independent sample preparations at the nominal concentration analyzed under the same conditions on the same day. As presented in Table 2, RSD values for the assay analytes (PB, ASA, CPM, and DMH) ranged from 0.62% to 1.06%, meeting the acceptance criterion of ≤2%. For impurities (SA, DMH impurity B, and DMH impurity C), repeatability RSD values ranged from 3.30 to 4.51%, all within the predefined limit of ≤5%.
  Intermediate precision was evaluated by repeating the analysis on different days using different instruments following the same procedure. Interday variability for assay analytes ranged from 0.12 to 1.76%, and for impurities from 1.89 to 8.70%, also meeting acceptance criteria (Table 2).

### LOD and LOQ

According to the ICH Q2 (R2) Analytical Procedure Validation Guideline, detection and quantitation limits can be estimated using different approaches, including the signal-to-noise ratio, the standard deviation of the response and slope of the calibration curve, or evaluation based on accuracy and precision at low concentration levels. For chromatographic methods exhibiting measurable baseline noise, the signal-to-noise approach is commonly applied.

**Figure 2.**
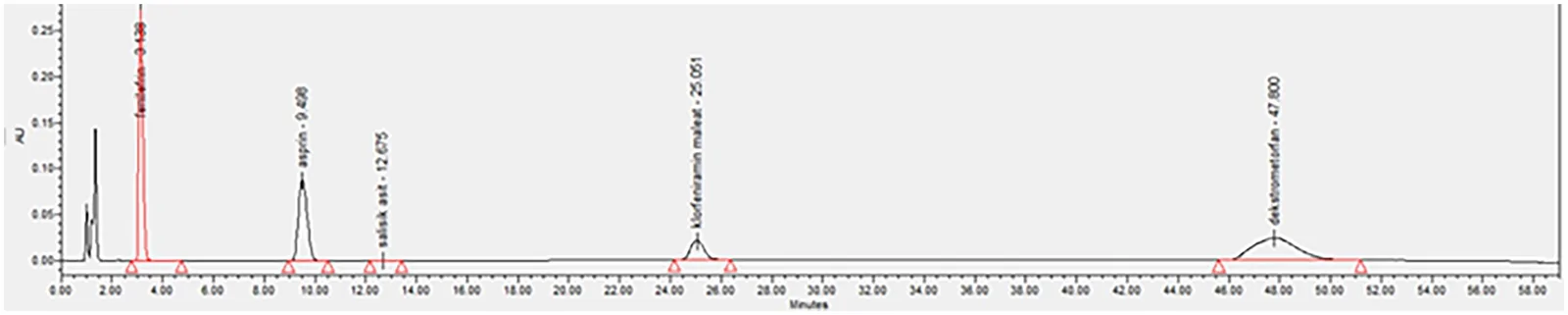
Representative chromatogram of the assay standard mixture containing ASA, DMH, PB, and CPM at 215 nm.

**Figure 3.**
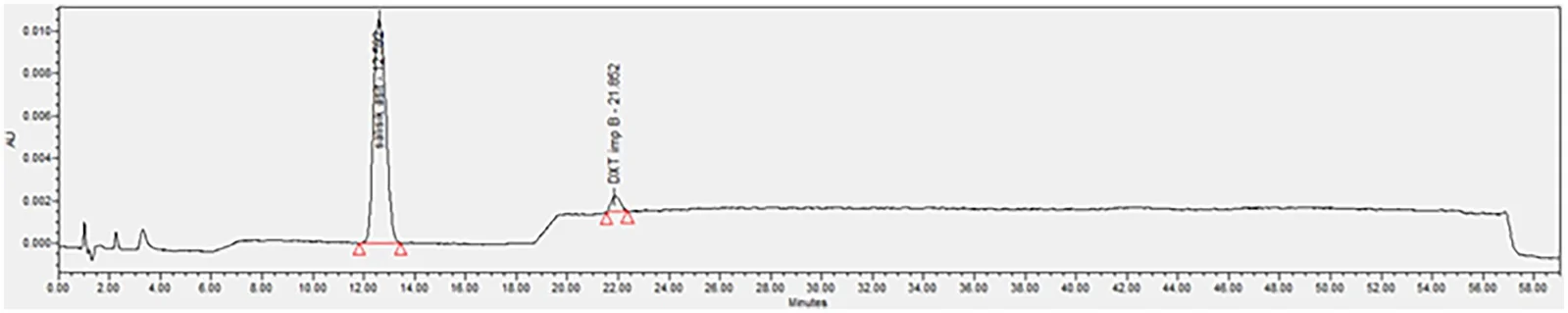
Impurity standard chromatogram showing SA, DMH impurity B (215 nm).

**Figure 4.**
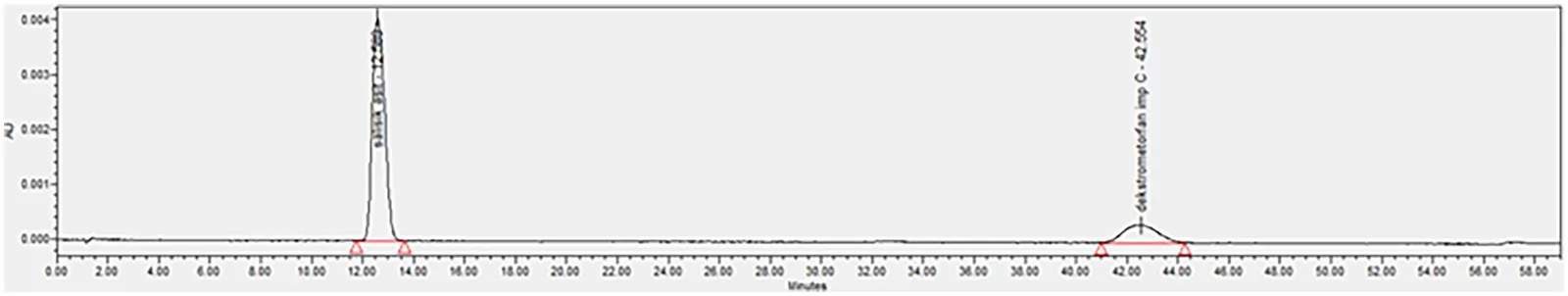
Impurity standard chromatogram showing SA, DMH impurity C (304 nm).

**Figure 5.**
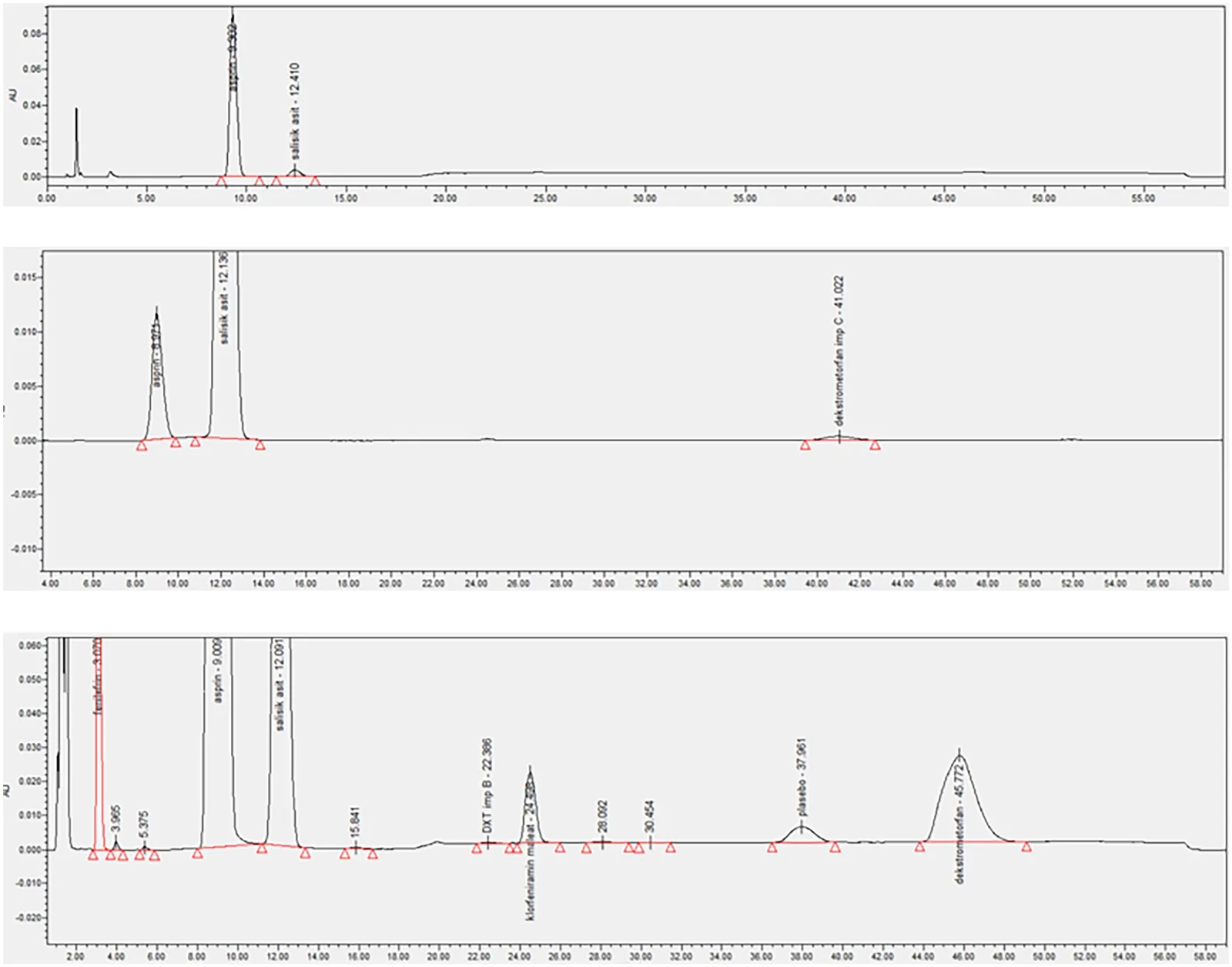
Representative chromatograms of sample solution showing complete separation of all active ingredients and related impurities at 215 nm and 304 nm.

**Figure 6.**
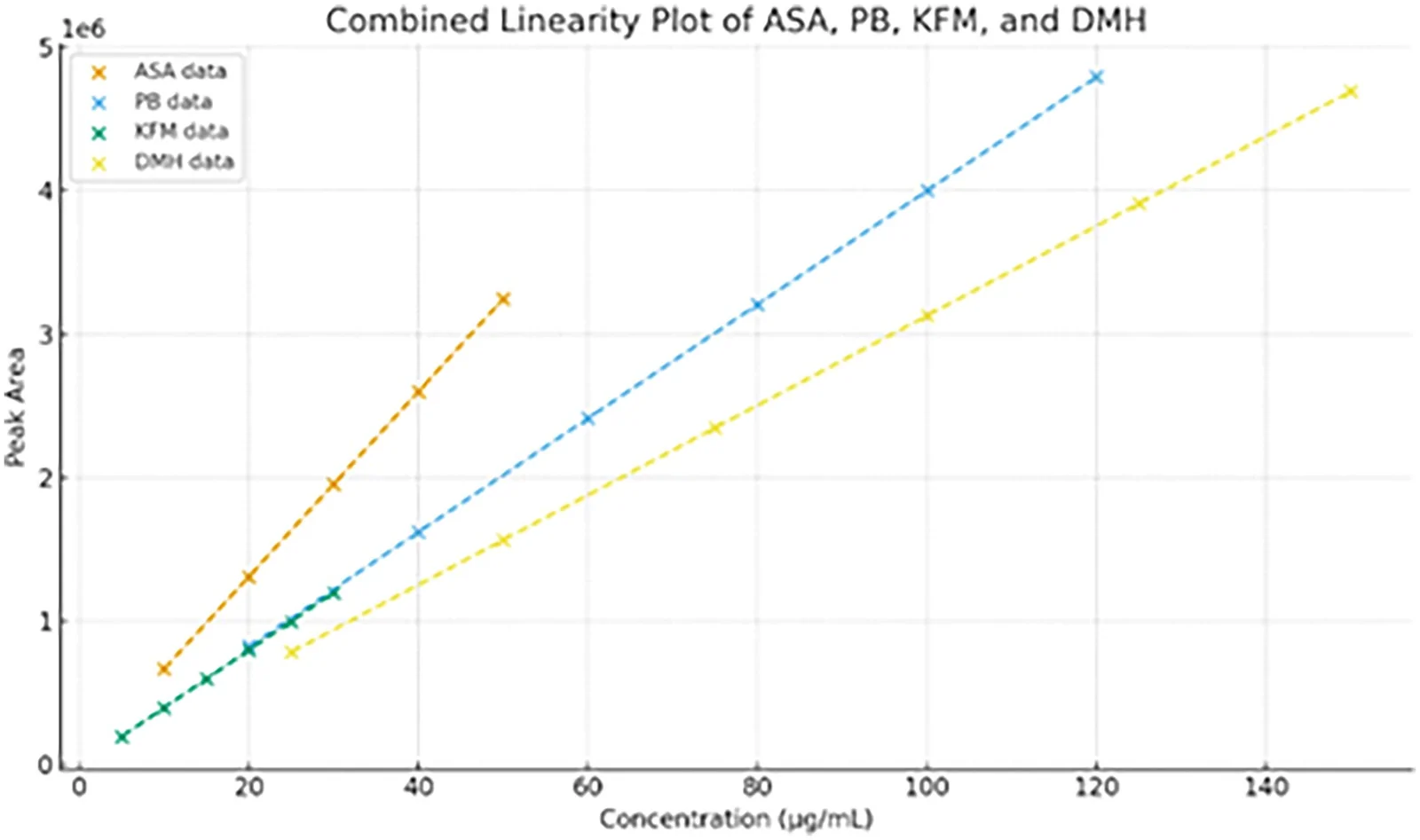
Combined linearity plots of ASA, PB, CPM, and DMH over the range of 25–150% of target concentration.

**Table 2.** LOD, LOQ, regression, and precision data

	Assay Analytes				Impurity Analytes		
Parameters	PB	ASA	CPM	DMH	SA	DMH Imp. B	DMH Imp. C
LOQ, µg/mL	0.24	0.27	0.71	2.30	0.13	0.35	0.20
LOD, µg/mL	0.07	0.08	0.21	0.69	0.04	0.11	0.06
Regression equation (y)							
Slope (b)	39 659	64 465	40 108	31 267	113 808	36 696	63 080
Intercept (a)	33 018	20 369	−5364	1077	3086	−257	−585
R²	1.0000	0.9980	0.9999	0.9999	0.9968	0.9932	0.9980
Correlation coefficient	1.0000	0.9990	0.9999	1.0000	0.9984	0.9966	0.9990
Y-intercept (% at 100%)	1.05	1.21	0.67	0.05	1.08	1.42	1.91
Precision (RSD, %; n = 6)	1.06	0.73	0.62	1.05	4.11	4.51	3.30
Intermediate precision (% difference)	0.12	1.76	0.76	1.64	8.70	2.08	1.89

**Table 3.** Accuracy results of assay for ASA, DMH, PB, and CPM at three concentration levels

	Amount Added, µg/mL				Amount Found, µg/mL				Recovery, %				RSD, %			
Level	ASA	DMH	PB	CPM	ASA	DMH	PB	CPM	ASA	DMH	PB	CPM	ASA	DMH	PB	CPM
80%	26.18	76.20	62.56	15.95	26.62	76.50	62.14	16.01	101.68	100.39	99.33	100.38	0.40	0.36	0.35	0.01
100%	32.50	95.25	78.20	19.94	32.80	94.46	77.08	19.82	100.92	99.17	98.58	99.40	1.52	0.72	0.46	1.41
120%	39.03	118.94	97.25	24.83	39.52	120.32	98.07	25.16	101.26	101.16	100.84	101.33	0.89	0.69	0.83	0.61

**Table 4.** Accuracy results of impurity for SA, DMH impurity B, and DMH impurity C at three concentration levels

	Amount Added, µg/mL			Amount Found, µg/mL			Recovery, %			**RSD, %**		
	SA	DMH Imp B	DMH Imp C	SA	DMH Imp B	DMH Imp C	SA	DMH Imp B	DMH Imp C	SA	DMH Imp B	DMH Imp C
LOQ	0.1305	0.3528	0.2016	0.1233	0.3583	0.2113	94.48	101.56	104.81	4.80	2.97	3.14
100%	2.6100	0.5040	0.5040	2.6266	0.5121	0.5317	100.64	101.61	105.50	4.54	1.16	2.67
120%	3.1320	0.6048	0.6048	3.0012	0.6235	0.6460	95.82	103.09	106.81	1.91	1.81	1.04

In the present study, the limits of detection (LOD) and quantitation (LOQ) for both assay analytes and related impurities were established using the signal-to-noise approach under the described chromatographic conditions, representing method detection and quantitation limits rather than instrumental limits.

A series of dilute standard solutions were analyzed and the signal-to-noise ratio was evaluated in the chromatographic baseline region surrounding the retention time of each analyte. A signal-to-noise ratio of approximately 3:1 was considered for the detection limit, whereas a ratio of at least 10:1 was used to define the quantitation limit.

Analytical performance at the LOQ level was further verified through replicate analyses to ensure acceptable performance at low concentration levels. The corresponding recovery and precision results at these concentration levels are included in the accuracy and precision validation studies.

As summarized in Table 2 (assay analytes and selected impurities), the method demonstrated adequate sensitivity, with LOD and LOQ values enabling reliable detection and quantification of the studied analytes and related impurities at trace levels.

### Ruggedness

Ruggedness was evaluated by introducing small deliberate variations in chromatographic conditions, including adjustments to column temperature (±2°C) and detection wavelength (±2 nm). Variations in mobile phase composition and pH were not included in the ruggedness evaluation because preliminary experiments demonstrated that small pH changes produced substantial retention shifts, indicating that strict pH control is a critical parameter for this method. Therefore, ruggedness evaluation was intentionally limited to column temperature and detection wavelength, which represent the most practically adjustable parameters under routine quality control conditions. The impact of these variations was assessed based on system suitability parameters. In this study, ruggedness was not assessed based on quantitative assay or selected impurity results; instead, it was evaluated solely through system suitability parameters to verify chromatographic performance under minor method variations. As shown in the ruggedness evaluation data (Tables 5 and 6), no significant changes were observed, and all system suitability criteria remained within the predefined acceptance limits.

**Table 5.** Effect of detection wavelength on system suitability parameters

	**213 nm/302** ^a^ **nm**			**215 nm/306** ^a^ **nm**		
	Retention time	Symmetry factor	Theoretical plates number	Retention time	Symmetry factor	Theoretical plates number
PB	2.75	1.28	1620	2.75	1.28	1623
ASA	8.22	1.14	3309	8.22	1.14	3318
CPM	21.57	1.08	16 312	21.57	1.08	16 302
DMH	38.46	1.14	4096	38.46	1.13	4107
SA	10.88	1.13	3059	10.88	1.12	3074
DMH Imp B	19.81	1.21	12 772	19.81	1.08	13 764
DMH Imp C^a^	34.80	1.12	5361	34.80	1.10	5430

a DMH Impurity C was evaluated at 302 nm and 306 nm, as specified in the method for this impurity.

**Table 6.** Effect of column temperature on system suitability parameters

	43°C			47°C		
	Retention time	Symmetry factor	Theoretical plates number	Retention time	Symmetry factor	Theoretical plates number
PB	2.79	1.27	1618	2.69	1.27	1645
ASA	8.22	1.14	3284	8.01	1.14	3330
CPM	21.86	1.09	15 139	21.21	1.06	16 582
DMH	39.72	1.16	3943	37.01	1.11	4119
SA	11.25	1.12	3134	10.51	1.12	3139
DMH Imp B	20.30	0.99	13 232	19.47	0.96	13 825
DMH Imp C	36.38	1.13	5418	33.87	1.09	5691

### Solution Stability

Solution stability was evaluated for both standard and sample solutions stored at tray temperature (5°C) by monitoring peak area changes over time. Acceptance criteria were defined as a variation within ±2% for assay and ±5% for impurities, with no significant change in peak shape, retention time, or response compared to initial (T_0_) measurements. The assay standard solution remained stable for up to 8 h at room temperature, while impurity standard solutions of SA, DMH impurity B, and DMH impurity C were stable for up to 48 h. Sample solutions containing PB, CPM, and DMH were also stable for up to 48 h without an increase in impurity levels. However, ASA in the sample solution showed rapid degradation to SA, indicating that ASA sample solutions must be freshly prepared and analyzed immediately.

### Forced Degradation Study

Forced degradation studies were performed at the nominal assay concentration to assess the stability-indicating capability of the method. Samples were subjected to acidic (1 N HCl, 60°C, 3 h), basic (1 N NaOH, 60°C, 30 min), oxidative (3% H_2_O_2_, ambient temperature, 30 min), thermal (60°C, 3 days), and photolytic (UV ≥200 W·h/m² and daylight, 7 days) stress conditions. Acidic and basic samples were neutralized before analysis. The developed method successfully resolved all analytes and their degradation products under each condition, with no coeluting peaks observed. Peak purity analysis confirmed that purity angles were below their respective thresholds for all analytes, demonstrating peak homogeneity and supporting method specificity.

As summarized in Table 7, ASA exhibited notable degradation under acidic, basic, oxidative, thermal, and photolytic stress, predominantly forming SA. In effervescent tablets, ASA degradation was more pronounced under thermal and photolytic conditions, likely due to moisture-driven hydrolysis. PB, CPM, and DMH showed greater stability, with assay values generally remaining within 90–110% under most stress conditions. Mass balance, evaluated for ASA only, was within 90–110% for most conditions, except for thermal stress in effervescent tablets (76.43%), where substantial degradation occurred.

**Table 7.** Summary of forced degradation study

Degradation Condition	PB %	CPM %	DMH %	ASA %	SA %	ASA + SA, %	% Mass Balance for (ASA + SA)
Initial (0 h)	96.49	98.98	97.37	102.25	2.76	105.01	–
Acidic hydrolysis (HCl, 60°C, 3 h)	89.92	90.92	93.33	83.54	13.74	97.28	92.64
Basic hydrolysis (NaOH, 60°C, 30 min)	87.16	90.75	94.99	82.72	16.99	99.71	94.95
Oxidative (H_2_O_2_, 60°C, 30 min)	81.17	77.12	91.04	76.76	21.54	98.30	93.61
Daylight (7 days)	31.93	94.99	98.18	78.76	26.53	105.29	100.27
UV light (254 nm, 7 days)	47.10	92.79	96.04	82.00	21.33	103.33	98.40
Thermal (60°C, 3 days)	36.38	64.16	58.74	50.76	29.50	80.26	76.43

### Application of the Method

The proposed stability-indicating RP-HPLC method was used for the analysis of Alka-Seltzer Plus Cold & Cough PowerFast Fizz effervescent tablets (citrus flavor), manufactured by Bayer, containing ASA (325 mg/tablet), dextromethorphan hydrobromide (DMH, 10 mg/tablet), PB (7.8 mg/tablet), and CPM (2 mg/tablet). Six replicate determinations were performed, and the assay and impurity results were calculated according to the equations described in the *Calculations* section. The results are summarized in Table 8.

**Table 8.** Analysis results for Alka-Seltzer Plus Cold & Cough PowerFast Fizz effervescent tablets

Component	Label claim/specification	Mean result found	Recovery/Compliance, %	RSD, %
ASA	325 mg/tablet	317.90 mg/tablet	97.82%	1.51
PB	7.8 mg/tablet	7.78 mg/tablet	99.74%	1.08
DMH	10 mg/tablet	10.25 mg/tablet	102.50%	1.53
CPM	2 mg/tablet	2.02 mg/tablet	101.00%	1.22
Salicylic acid	NMT 8%	4.28%	Complies	2.68
DMH Impurity B	NMT 0.5%	Not detected	Complies	–
DMH Impurity C	NMT 0.5%	Not detected	Complies	–

The mean assay results were found to be 317.90 mg/tablet for ASA, 7.78 mg/tablet for PB, 10.25 mg/tablet for DMH, and 2.02 mg/tablet for CPM, corresponding to 97.82, 99.74, 102.50, and 101% of the label claim, respectively. The RSD values for all assay analytes were below 2%.

For impurity analysis, SA was detected at a mean level of 4.28%, which was below the limit specified in the USP monograph for Aspirin Effervescent Tablets for Oral Solution (NMT 8%) (16). Dextromethorphan impurities B and C were not detected in the analyzed samples. The European Pharmacopoeia monograph for dextromethorphan hydrobromide specifies NMT 0.5% for the related impurities (9).

## Discussion

### Method Validation Outcomes

The validation results collectively demonstrate that the developed RP-HPLC method meets the requirements outlined in ICH Q2 (R2) for quantitative analysis of both active pharmaceutical ingredients and selected impurities. The method exhibited excellent linearity across the studied concentration ranges for all analytes, supporting reliable quantification over a broad working range. Accuracy and precision results confirmed that the method provides consistent and repeatable measurements, with variability remaining within acceptable limits for both assay and impurity determinations.

System suitability parameters, including theoretical plate numbers, tailing factors, and injection repeatability, indicated stable chromatographic performance under the optimized conditions. Minor deliberate changes in selected analytical conditions were evaluated using system suitability criteria, and acceptable chromatographic performance was maintained under the tested conditions. Adequate sensitivity was achieved for impurity determination, with LOQ levels sufficiently low to allow reliable detection and quantification of degradation products and selected impurities at trace levels. Collectively, these outcomes confirm that the method is fit for its intended purpose in the analysis of the evaluated effervescent tablet formulation.

### Stability-Indicating Capability

The stability-indicating capability of the developed RP-HPLC method was evaluated through forced degradation studies conducted under acidic, basic, oxidative, thermal, and photolytic stress conditions. Under all applied stress scenarios, the method provided clear separation between ASA and its primary degradation product, SA, as well as between the active pharmaceutical ingredients and selected related impurities evaluated in this study, including dextromethorphan impurities B and C. The absence of coeluting peaks and the peak purity results across stress conditions indicate that the method is capable of selectively monitoring chemical changes occurring during degradation.

ASA exhibited pronounced susceptibility to hydrolytic, oxidative, thermal, and photolytic stress, with degradation predominantly resulting in the formation of SA. This behavior is consistent with the known instability of ASA and was particularly evident in the effervescent tablet matrix, where moisture-related effects can accelerate hydrolysis. In contrast, dextromethorphan hydrobromide, PB, and CPM demonstrated comparatively higher stability under most stress conditions, with assay values generally remaining within acceptable limits. These differences in degradation behavior highlight the importance of a stability-indicating method capable of resolving both highly labile and relatively stable components within a single analytical program.

Mass balance considerations were evaluated specifically for ASA by monitoring the combined content of ASA and its major degradation product, SA, under stress conditions. For most degradation scenarios, the combined content of ASA and SA remained within acceptable limits, indicating that the principal degradation pathway of ASA was adequately captured by the analytical procedure. Deviations observed under strong alkaline and thermal stress conditions in the evaluated effervescent formulation can be attributed to extensive degradation and matrix-driven effects, rather than limitations of chromatographic resolution. Overall, the forced degradation results confirm that the developed method can be applied for stability testing and can effectively support the assessment of degradation behavior in the evaluated moisture-sensitive effervescent tablet formulation.

## Conclusions

A stability-indicating RP-HPLC method was developed and validated for the determination of ASA, dextromethorphan hydrobromide, PB, CPM, and their selected impurities in a multicomponent effervescent tablet formulation. The method demonstrated compliance with ICH Q2 (R2) validation requirements and proved suitable for the quantitative analysis of both active pharmaceutical ingredients and the evaluated impurities.

Forced degradation studies confirmed the stability-indicating nature of the method, enabling effective separation of active components from degradation products under a range of stress conditions. The method can be applied for routine quality control and stability testing for the evaluated effervescent tablet formulation.

Overall, this analytical approach meets regulatory expectations for the evaluated effervescent tablet formulation and can be readily implemented in pharmaceutical quality control laboratories to support product quality assurance throughout the product life cycle.

## Supplementary Material

qsag070_Supplementary_Data

## CRediT Author Statement

Selin Atlı: Conceptualization, Methodology, Investigation, Data Curation, Formal Analysis, Validation, Visualization, Writing—Original Draft. Özlem Aksu Dönmez: Supervision, Writing—Review & Editing, Project Administration, Funding Acquisition.
