# Supplementary material for: Stability-Indicating RP-HPLC Determination of Acetylsalicylic Acid, Dextromethorphan Hydrobromide, Phenylephrine Bitartrate, and Chlorpheniramine Maleate in an Effervescent Tablet Formulation
Source: J AOAC Int. 2026 Aug 7;109(6):887–96. doi: 10.1093/jaoacint/qsag070 (PMC13732704; doi:10.1093/jaoacint/qsag070)
Supplement: Supplementary file 1 [file qsag070_supplementary_data.zip › qsag070_Supplementary_Data/aoac-25-0299-File007.pdf]

Divi's Laboratories Limited

ANALYTICAL REPORT

Product: Ent-17-methylmorphinan-3-ol				Rev. No.	SGI/04/02
Grade	IN-HOUSE	Q.C.R No.	DIS/YC/2601	Sl. No.	SAM-484AB
Batch Number	IPS004SGC	Mfg. date	20th MAR 2019	Total pages	1
Quantity	50 mg	Valid till	17th DEC 2022	Page No.	1

Sl. No.	Test	Observation
01	Description	Off-white powder
02	Identification by<br>i) IR Spectrum<br>ii) Mass Spectrum	 Concordant with that of standard spectrum<br>Conforms
03	Purity by HPLC (%area)	99.86%
04	Loss on drying	0.13%w/w
05	Residue on ignition	0.02%w/w
06	Potency	99.71%w/w

	Analyst	Person in-charge of testing
Signature	16/11/22	16/11/22

FACTORY : UNIT-1, Lingojigudem Village, Choutuppal Mandal, Yadadri Bhuvanagiri District, Telangana - 508 252, INDIA.
Tel. : +91-8694 257001, Fax : +91-8694 257006, CIN : L24110TG1990PLC011854
CORPORATE OFFICE : DIVI Towers, 1-72/23(P)/DIVIS/303, Cyber Hills, Gachibowli, Hyderabad - 500 032, Telangana, India.
Tel. : +91-40-2378 6300 / 400, Fax : +91-40-2378 6460
E-mail : mail@divislabs.com, Website : www.divislabs.com.

Divi's Laboratories Limited

ANALYTICAL REPORT

Product: Ent-17-methylmorphinan-3-ol				Rev. No.	SGI/04/02
Grade	IN-HOUSE	Q.C.R No.	DIS/YC/2601	Sl. No.	SAM-484AB
Batch Number	IPS004SGC	Mfg. date	20th MAR 2019	Total pages	1
Quantity	50 mg	Valid till	17th DEC 2022	Page No.	1

Sl. No.	Test	Observation
01	Description	Off-white powder
02	Identification by<br>i) IR Spectrum<br>ii) Mass Spectrum	 Concordant with that of standard spectrum<br>Conforms
03	Purity by HPLC (%area)	99.86%
04	Loss on drying	0.13%w/w
05	Residue on ignition	0.02%w/w
06	Potency	99.71%w/w

	Analyst	Person in-charge of testing
Signature	16/11/22	16/11/22
