# Supplementary material for: Stability-Indicating RP-HPLC Determination of Acetylsalicylic Acid, Dextromethorphan Hydrobromide, Phenylephrine Bitartrate, and Chlorpheniramine Maleate in an Effervescent Tablet Formulation
Source: J AOAC Int. 2026 Aug 7;109(6):887–96. doi: 10.1093/jaoacint/qsag070 (PMC13732704; doi:10.1093/jaoacint/qsag070)
Supplement: Supplementary file 1 [file qsag070_supplementary_data.zip › qsag070_Supplementary_Data/aoac-25-0299-File008.pdf]

Divi's Laboratories Limited

ANALYTICAL REPORT

Product: Ent-3-methoxy-17-methylmorphinan-10-one				Rev. No.	SGI/02/02
Grade	IN-HOUSE	Q.C.R No.	DU1/WS/2204/000124	Sl. No.	SAM-485AB
Batch Number	IPS006SGA	Mfg. date	03rd MAR 2022	Total pages	1
Quantity	50 mg	Valid till	01st MAR 2025	Page No.	1

Sl. No.	Test	Observation
01	Description	Yellow powder
02	Identification by IR Spectrum	Concordant with standard spectrum
03	Identification by Mass Spectrum	Conforms
04	Purity by HPLC	95.55%

	Analyst	Person in-charge of testing
Signature	[signature] 16/11/22	[signature] 16/11/22

Divi's Laboratories Limited

ANALYTICAL REPORT

Product: Ent-3-methoxy-17-methylmorphinan-10-one				Rev. No.	SGI/02/02
Grade	IN-HOUSE	Q.C.R No.	DU1/WS/2204/000124	Sl. No.	SAM-485AB
Batch Number	IPS006SGA	Mfg. date	03rd MAR 2022	Total pages	1
Quantity	50 mg	Valid till	01st MAR 2025	Page No.	1

Sl. No.	Test	Observation
01	Description	Yellow powder
02	Identification by IR Spectrum	Concordant with standard spectrum
03	Identification by Mass Spectrum	Conforms
04	Purity by HPLC	95.55%

	Analyst	Person in-charge of testing
Signature	[signature] 16/11/22	[signature] 16/11/22
