# Supplementary material for: Stability-Indicating RP-HPLC Determination of Acetylsalicylic Acid, Dextromethorphan Hydrobromide, Phenylephrine Bitartrate, and Chlorpheniramine Maleate in an Effervescent Tablet Formulation
Source: J AOAC Int. 2026 Aug 7;109(6):887–96. doi: 10.1093/jaoacint/qsag070 (PMC13732704; doi:10.1093/jaoacint/qsag070)
Supplement: Supplementary file 1 [file qsag070_supplementary_data.zip › qsag070_Supplementary_Data/aoac-25-0299-File009.pdf]

DEXTROMETHORPHAN HYDROBROMIDE MONOHYDRATE

Dextromethorphani hydrobromidum monohydricum

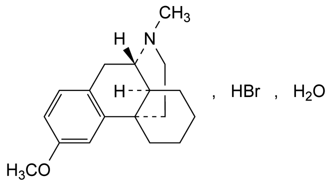

$C_{18}H_{26}BrNO,H_2O$ M_r 370.3
[6700-34-1]

DEFINITION

ent-3-Methoxy-17-methylmorphinan hydrobromide monohydrate.

Content: 99.0 per cent to 101.0 per cent (anhydrous substance).

CHARACTERS

Appearance: almost white, crystalline powder.

Solubility: sparingly soluble in water, freely soluble in ethanol (96 per cent).

mp: about 125 °C, with decomposition.

IDENTIFICATION

First identification: carry out either tests A, B, E or tests B, C, E.

Second identification: A, D, E.

A. Specific optical rotation (*2.2.7*): + 28 to + 30 (anhydrous substance).
Dissolve 0.200 g in a 10.3 g/L solution *hydrochloric acid R* and dilute to 10.0 mL with the same solution.

B. Infrared absorption spectrophotometry (*2.2.24*).
Comparison: *dextromethorphan hydrobromide CRS.*

C. Enantiomeric purity (see Tests).

D. Thin-layer chromatography (*2.2.27*).
Test solution. Dissolve 25 mg of the substance to be examined in *methanol R* and dilute to 10 mL with the same solvent.
Reference solution. Dissolve 25 mg of *dextromethorphan hydrobromide CRS* in *methanol R* and dilute to 10 mL with the same solvent.
Plate: *TLC silica gel G plate R.*
Mobile phase: *concentrated ammonia R*, *methylene chloride R*, *methanol R*, *ethyl acetate R*, *toluene R* (2:10:13:20:55 *V/V/V/V/V*).
Application: 5 µL.
Development: over 2/3 of the plate.
Drying: in air.
Detection: spray with *potassium iodobismuthate solution R2.*
Results: the principal spot in the chromatogram obtained with the test solution is similar in position and size to the principal spot in the chromatogram obtained with the reference solution.

E. It gives reaction (a) of bromides (*2.3.1*).

TESTS

Appearance of solution. The solution is clear (*2.2.1*) and colourless (*2.2.2, Method II*).

Dissolve 1.0 g in *ethanol (96 per cent) R* and dilute to 20 mL with the same solvent.

Acidity or alkalinity. Dissolve 0.4 g in *carbon dioxide-free water R* with gentle heating, cool and dilute to 20 mL with the same solvent. Add 0.1 mL of *methyl red solution R* and 0.2 mL of *0.01 M sodium hydroxide.* The solution is yellow. Not more than 0.4 mL of *0.01 M hydrochloric acid* is required to change the colour of the indicator to red.

Enantiomeric purity. Liquid chromatography (*2.2.29*).

Solvent mixture: *water R*, *methanol R* (10:90 *V/V*).

Test solution. Dissolve 0.100 g of the substance to be examined in the solvent mixture and dilute to 10.0 mL with the solvent mixture.

Reference solution (a). Dissolve 5 mg of *dextromethorphan for system suitability CRS* (containing impurity E) in the solvent mixture and dilute to 0.5 mL with the solvent mixture.

Reference solution (b). Dilute 1.0 mL of the test solution to 100.0 mL with the solvent mixture. Dilute 1.0 mL of this solution to 10.0 mL with the solvent mixture.

Column:

– *size*: l = 0.25 m, Ø = 4.6 mm;
– *stationary phase*: *vancomycin-bonded silica gel for chiral separation R* (5 µm) with a pore size of 20 nm.

Mobile phase: mix 10 volumes of a 1.54 g/L solution of *ammonium acetate R* previously adjusted to pH 4.1 with *phosphoric acid R* and 90 volumes of *methanol R1*.

Flow rate: 1.0 mL/min.

Detection: spectrophotometer at 225 nm.

Injection: 4 µL.

Run time: 1.5 times the retention time of dextromethorphan.

Identification of impurities: use the chromatogram supplied with *dextromethorphan for system suitability CRS* and the chromatogram obtained with reference solution (a) to identify the peak due to impurity E.

Relative retention with reference to dextromethorphan (retention time = about 11 min): impurity E = about 1.2.

System suitability: reference solution (a):

– *resolution*: minimum 2.0 between the peaks due to dextromethorphan and impurity E.

Calculation of percentage content:

– for impurity E, use the concentration of dextromethorphan hydrobromide monohydrate in reference solution (b).

Limit:

– *impurity E*: maximum 0.10 per cent.

Related substances. Liquid chromatography (*2.2.29*).

Test solution. Dissolve 10.0 mg of the substance to be examined in the mobile phase and dilute to 10.0 mL with the mobile phase.

Reference solution (a). Dissolve 2 mg of *dextromethorphan impurity A CRS* in 2 mL of the test solution and dilute to 25 mL with the mobile phase.

Reference solution (b). Dilute 1.0 mL of the test solution to 100.0 mL with the mobile phase. Dilute 1.0 mL of this solution to 10.0 mL with the mobile phase.

Column:

– *size*: l = 0.25 m, Ø = 4.6 mm;
– *stationary phase*: *end-capped octadecylsilyl silica gel for chromatography R* (5 µm).

Mobile phase: dissolve 3.11 g of *docusate sodium R* in a mixture of 400 mL of *water for chromatography R* and 600 mL of *acetonitrile R*, add 0.56 g of *sodium nitrate R* and adjust to apparent pH 2.0 with *glacial acetic acid R.*

Flow rate: 1.0 mL/min.

Detection: spectrophotometer at 280 nm.

Injection: 20 µL.

Run time: twice the retention time of dextromethorphan.

Identification of impurities: use the chromatogram obtained with reference solution (a) to identify the peak due to impurity A.

Relative retention with reference to dextromethorphan (retention time = about 19 min): impurity A = about 1.1.

System suitability: reference solution (a):

– *resolution*: minimum 1.5 between the peaks due to dextromethorphan and impurity A.

Calculation of percentage contents:

– for each impurity, use the concentration of dextromethorphan hydrobromide monohydrate in reference solution (b).

Limits:
- *unspecified impurities*: for each impurity, maximum 0.10 per cent;
- *total*: maximum 0.5 per cent;
- *reporting threshold*: 0.05 per cent.

***N,N*-Dimethylaniline**: maximum 10 ppm.

Dissolve 0.5 g with heating in 20 mL of *water R*. Allow to cool, add 2 mL of *dilute acetic acid R* and 1 mL of a 10 g/L solution of *sodium nitrite R* and dilute to 25 mL with *water R*. The solution is not more intensely coloured than a reference solution prepared at the same time and in the same manner using 20 mL of a 0.25 mg/L solution of *N,N-dimethylaniline R*.

Water (*2.5.12*): 4.0 per cent to 5.5 per cent, determined on 0.200 g.

Sulfated ash (*2.4.14*): maximum 0.1 per cent, determined on 1.0 g.

ASSAY

Dissolve 0.300 g in a mixture of 5.0 mL of *0.01 M hydrochloric acid* and 20 mL of *ethanol (96 per cent) R*. Titrate with *0.1 M sodium hydroxide*, determining the end-point potentiometrically (*2.2.20*). Read the volume added between the 2 points of inflexion.

1 mL of *0.1 M sodium hydroxide* is equivalent to 35.23 mg of $C_{18}H_{26}BrNO$.

STORAGE

Protected from light.

IMPURITIES

Specified impurities: E.

Other detectable impurities (the following substances would, if present at a sufficient level, be detected by one or other of the tests in the monograph. They are limited by the general acceptance criterion for other/unspecified impurities and/or by the general monograph *Substances for pharmaceutical use (2034)*. It is therefore not necessary to identify these impurities for demonstration of compliance. See also *5.10. Control of impurities in substances for pharmaceutical use*)*: A, B, C, D.*

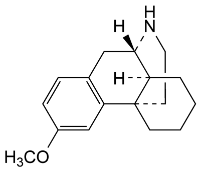

A. *ent*-3-methoxymorphinan,

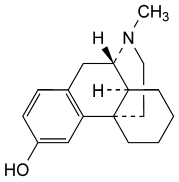

B. *ent*-17-methylmorphinan-3-ol,

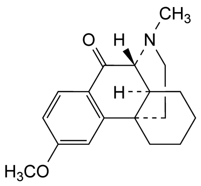

C. *ent*-3-methoxy-17-methylmorphinan-10-one,

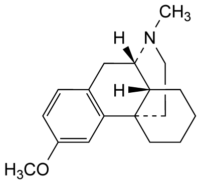

D. 3-methoxy-17-methyl-9α,13α-morphinan,

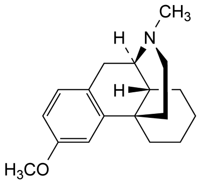

E. 3-methoxy-17-methylmorphinan (levomethorphan).
