# Supplementary material for: Stability-Indicating RP-HPLC Determination of Acetylsalicylic Acid, Dextromethorphan Hydrobromide, Phenylephrine Bitartrate, and Chlorpheniramine Maleate in an Effervescent Tablet Formulation
Source: J AOAC Int. 2026 Aug 7;109(6):887–96. doi: 10.1093/jaoacint/qsag070 (PMC13732704; doi:10.1093/jaoacint/qsag070)
Supplement: Supplementary file 1 [file qsag070_supplementary_data.zip › qsag070_Supplementary_Data/aoac-25-0299-File010.pdf]

Journal of the Association of Public Analysts (Online) 2009 **37** 40-60
D Thorburn Burns *et al*

A Tutorial Discussion of the use of the terms "Robust" and "Rugged" and the Associated Characteristics of "Robustness" and "Ruggedness" as used in Descriptions of Analytical Procedures

Duncan Thorburn Burns[1], Klaus Danzer[2] and Alan Townshend[3]

[1]School of Chemistry,The Queen's University of Belfast, Belfast BT9 5EB, N. Ireland, UK
[2]Institute of Inorganic and Analytical Chemistry, Friedrich Schiller University of Jena, Lessingstrasse 8, Germany
[3]Department of Chemistry, The University of Hull, Hull, HU6 7RX, UK

Summary

*The terms **robust** and **rugged** are clearly defined and their uses distinguished along with the associated characteristics of **robustness** and **ruggedness.** It is shown that the characteristics of **robustness** and **ruggedness** which express resistance against conditions and influences which decrease both precision and accuracy of analytical results, obtained by a particular procedure in a given laboratory or in different laboratories, can be treated quantitatively by the introduction of two new concepts, namely, **relative robustness** and **relative ruggedness**.*

Introduction

There is considerable confusion in the scientific journal and monograph literature with regard to the use of the terms **robust** and **rugged** and of the associated characteristics **robustness** and **ruggedness** as applied to the description of analytical methods. Many authors use the two terms and their associated characteristics as if they were synonymous [1-22] . Others use only one term and/or characteristic, namely **robust/robustness** [23-38] or **rugged/ruggedness** [39-57]. A few authors distinguish their use as between the areas of intra- and inter-laboratory studies [58-68] or restrict the use of **robust/ robustness** to the statistical interpretation of data and of **rugged/ruggedness** to experimental design of intra-laboratory studies prior to a collaborative trial [69]. The use of the term **robust** in connection with statistical tests on data is supported by many chemists and statisticians [70-77].

Prior to the present study these terms and the associated characteristics of these terms have not been defined by IUPAC, ISO or similar bodies. Indeed, within IUPAC documents confusion exists with the use of ruggedness, in one case [45] the explanation of ruggedness implies an inter-laboratory use, by giving laboratories in the list of variables, whereas in a later document [53], ruggedness is used in a single-laboratory situation.

The International Conference on Harmonization of Technical Requirements for Registration of Pharmaceuticals for Human Use (ICH) defines **Robustness** as follows: "The robustness of an analytical procedure is a measure of its capacity to remain unaffected by small, but deliberate variations in method parameters and provides an indication of its reliability during normal usage." [23, 24]. They state this should be evaluated at the development stage i.e.

from intra-laboratory experimentation and list examples of typical variations, those for liquid chromatography and for gas chromatography to be examined. The British Pharmacopoeia (BP) [38] additionally gives a list of challenges to the normal operating procedure for robustness testing a procedure which uses infrared spectrophotometry. The US Pharmacopeia National Formulary (USP.NF) [59] has adopted the ICH definition of **robustness** and defines **Ruggedness** as: "The ruggedness of an analytical method is the degree to of reproducibility of test results obtained by the analysis of the same samples under a variety of conditions such as different laboratories, different analysts, different instruments, different lots of reagents, different elapsed assay times, different assay temperatures, different days, etc. Ruggedness is normally expressed as the lack of influence of operational and environmental factors of the analytical method. Ruggedness is a measure of reproducibility of test results under the variation in conditions normally expected from laboratory to laboratory and analyst to analyst." This definition clearly refers to inter-laboratory studies. The ICH and the USP.NF give procedures for the qualitative evaluation of **robustness** and **ruggedness,** respectively, but not for their numerical determination.

Wahlich and Carr [58], whilst discussing chromatographic system suitability tests, appear to be the first to use **ruggedness** (defined as the effect of operational parameters on the methods suitability) and **robustness** (referred to as the method´s suitability to be transferred to another laboratory) in a hierarchical sense. The areas of application of these two characteristics were reversed by Zeaiter et al. [63] in a discussion of the **robustness** of models developed by multivariate calibration for infrared spectroscopic data. They noted the confusion in the literature with the use of the characteristics **ruggedness** and **robustness** and that **ruggedness** was a property hierarchically above **robustness** as put forward by the Canadian Drugs Directorate in their three level testing system. In this system Level I refers to the ICH (intra-laboratory) definition of **robustness** [23] and should include verification of reproducibility by using a second analyst. In Level II testing, the effects of more severe changes in conditions are examined when the method is intended to be applied in a different laboratory with different equipment. Level III considers "a full collaborative testing", which is rarely done.

The only official document to distinguish the use of **robustness** and **ruggedness** is the USP.NF [59]. The definition for **robustness** in the USP.NF and in the British Pharmacopoeia have their origin in the ICH documents CPMP/ICH/381/95 [23] and CPMP/ICH/281 [24]. The material in CPMH/ICH/381/95 also appears in the official Federal Register of the Food and Drug Administration [27]. The European Commission only refer to one characteristic for analytical methods, namely **ruggedness** [52]**,** for the outcome from what is clearly an intra-laboratory test. Thus the European Commission and others [39–57] are out of line with what has been said to be traditional usage [61], namely **robustness** for the outcome from an intra-laboratory test, as recommended herein. This usage is consistent with the earlier IUPAC description of **ruggedness** which implied an inter-laboratory experiment [46].

To produce a robust or a rugged analytical procedure it is essential that the instruments used

in the analytical procedure produce acceptable data. The process of ensuring the precision and accuracy of an analytical instrument used in an analytical procedure and its descriptive terminology has been recorded by Bansal *et al.* following an American Association of Pharmaceutical Scientists (AAPS) workshop, “A Scientific Approach to Analytical Instrument Validation” [78]. The participants agreed that the term **validation** should be used to refer to the overall analytical process, and that the term **qualification** be used for the procedure for ensuring that an instrument was producing data of the required precision and accuracy.

Although the literature cited above refers almost exclusively to the analysis of pharmaceutical products, the clear distinction between the descrpitors **robust** and **rugged** is of wider application in other fields of analysis for regulatory purposes such those of human foods, animal feedstuffs, environmental samples and of artricles subject to tariff/customs control.

Definitions

The following definitions are now recommended:

Robust / Robustness / Relative Robustness:

A ***robust*** *analytical method* is one which exhibits a high degree of robustness as determined in an intra-laboratory study.

Robustness of an analytical method is the property that indicates insensitivity against changes of known operational parameters on the results of the method and hence its suitability for its defined purpose. This is ascertained during the method development processes and determines the allowable (acceptable) limits for all critical parameters that affect measured values for analytes and provides information on a method’s practability. It may be defined by the value of a method’s ***Relative Robustness.***

The ***Relative Robustness*** of an analytical method is defined as the ratio of the ideal signal for an uninfluenced method compared to the signal for a method subject to known operational parameters as determined in an intra-laboratory experiment [see equation (4) below].

Rugged / Ruggedness / Relative Ruggedness:

A ***rugged*** *analytical method* is one that exhibits a high degree of ruggedness after an inter-laboratory experiment**.**

Ruggedness of an analytical method is the property that indicates insensitivity against inadvertent changes of known operational variables and in addition any variations (not discovered in intra-laboratory experiments) which may be revealed by inter-laboratory studies. Such experiments are normally conducted on well defined procedures following robustness experiment (or experiments) and provide information on a procedure’s

interlaboratory transferability. It may be defined by the value of the methods' ***Relative Ruggedness***.

The ***Relative Ruggedness*** of an analytical method is defined by the ratio of the ideal signal for uninfluenced method compared to the signal for a method subject to known and unknown operational parameters as determined in an inter-laboratory experiment [see equation (6) below].

The Quantitative Evaluation of Robustness and Ruggedness

It is important to obtain a measure of the robustness and ruggedness of an analytical method based on experimental data. The means of making such calculations are described below, and examples are given in Appendix I. It follows that if full use of these evaluations is to be made, criteria must be established by interested parties to define the limits for these parameters beyond which the analytical procedure is unacceptable. If a method is found to be insufficiently robust or rugged, modifications to the method must be investigated to mitigate the situation. Knowledge of which parameters contribute most to the lack of sufficient robustness or ruggedness, information that is normally obtained when making an experimental evaluation of these parameters, is crucial in this respect.

The conditions and influences which decrease both precision and accuracy of analytical results obtained in a given laboratory or in different laboratories are now considered in detail and their effects on **robustness** and on **ruggedness** described quantitatively.

In ideal circumstances, a measured signal of an analyte A (y_A) is caused only by this analyte and nothing else. The measured signal is proportional to the (ideal) ***analyte sensitivity***,

$$S_{AA} = \frac{\partial y_A}{\partial x_A} \approx \frac{\Delta y_A}{\Delta \mathrm{x}_\mathrm{A}},$$

and the ***analyte amount***, x_A (content, concentration) plus the experimental error (analytical error) e_A,

$$y_A = S_{AA} x_A + e_A . \qquad (1)$$

In analytical practice, under real circumstances, a measured ***gross signal***, y_A, of an analyte A is made up of the following five component parts:

(1) the major part, as a rule[1], is caused by the **analyte** and characterized by the ***analyte sensitivity***, S_{AA}, and the ***analyte amount***, x_A, see above. Additionally

[1] In exceptional cases, e.g. in NIR spectrometry, the analyte may cause a minor part of the measured gross signal

the signal is caused, in variable degrees, by

(2) a possible **blank**, y_{A0}, that can be recognized and minimized as well as considered arithmetically

(3) **known interferences** of known species *i* = *B*, *C*, ..., *N* which are characterized by their ***cross sensitivities*** (partial sensitivities), $S_{Ai} = \frac{\partial y_A}{\partial x_i} \approx \frac{\Delta y_A}{\Delta x_i}$, and their ***amounts***, x_i

(4) **known influences of known factors** f_j (j = 1, ..., m) such as temperature, pressure, pH, characterized by their *(specific) influencing strength*, $I_{Aj} = \frac{\partial y_A}{\partial x_j} \approx \frac{\Delta y_A}{\Delta x_j}$, and their *actual value*, x_j

(5) **unknown inferferences and influences of unknown factors** u_k (k = 1, ..., p) their type and number *z* is not known a priori. Because neither their *cross sensitivities* or *influencing strengths* nor their *amounts* or *actual values* are known, these unknowns become apparent by error contributions e_k and are typical causes of inter-laboratory effects.

The gross signal may be expressed mathematically as follows:

$$y_A = y_{A0} + S_{AA}x_A + \sum_{i=B}^{N} S_{Ai}x_i + \sum_{j=1}^{m} I_{Aj}x_j + \sum_{k=1}^{p} u_k + e_A \qquad (2)$$

Known interferences and known influencing factors are the effects that can be studied ***within each individual laboratory***, appropriately by experimental variation of the related quantities according to multifactorial design. Variations caused by unknown interferents and unknown influence factors become apparent ***between various laboratories*** and can be revealed by inter-laboratory studies.

In intra- and inter-laboratory experiments it is axiomatic that the equipment is functioning within specification. The process of validation of instruments has been called “qualification” of analytical instruments and procedures described by Bansal et al [63].

Mathematical models of robustness and ruggedness

The **robustness** of the determination of an analyte *A* in the presence of some accompanying species, *i* = *B*, ..., *N* and under influence of various factors f_j (j = 1, ..., m) is in reciprocal proportion to the sum of all their cross sensitivities, $|S_{Ai}|$, multiplied by the actual amounts, x_i, and the specific influencing strengths, $|I_{Aj}|$, of the factors multiplied by their actual values, x_j [60]

$$rob(A/B,...,N;\, f_1,...,\, f_m) = \frac{1}{\sum_{i=B}^{N} |S_{Ai}| x_i + \sum_{j=1}^{m} |I_{Aj}| x_j} \tag{3}$$

The **robustness** of a method is the better the higher the value of $rob(A/B,...,N;\, f_1,...,\, f_m)$; in the ideal case it would be infinite. In analytical practice it should be more helpful to have values in a more meaningful range. This can be achieved by calculating the **relative robustness** which is related to the ideal signal $S_{AA}x_A$. Specifically, the relative robustness is defined as follows

$$rob_{rel}(A/B,...,N;\, f_1,...,\, f_m) = \frac{S_{AA}x_A}{S_{AA}x_A + \sum_{i=B}^{N} |S_{Ai}| x_i + \sum_{j=1}^{m} |I_{Aj}| x_j} \tag{4}$$

The relative robustness can have values between **0** (no robustness) and **1** (ideal robustness).

For **ruggedness** effects of the unknown interferents and influencing factors must, additionally, be considered,

$$rug(A/B,...,N;\, f_1,...,\, f_m; u_1,...,u_p) = \frac{1}{\sum_{i=B}^{N} |S_{Ai}| x_i + \sum_{j=1}^{m} |I_{Aj}| x_j + \sum_{k=1}^{p} |u_k|}\ . \tag{5}$$

Relative ruggedness can be expressed by:

$$rug_{rel}(A/B,...,N;\, f_1,...,\, f_m; u_1,...,u_p) = \frac{S_{AA}x_A}{S_{AA}x_A + \sum_{i=B}^{N} |S_{Ai}| x_i + \sum_{j=1}^{m} |I_{Aj}| x_j + \sum_{k=1}^{p} |u_k|} \tag{6}$$

which can have values between **0** (no ruggedness) and **1** (ideal ruggedness), in a similar manner to relative robustness.

Testing robustness and ruggedness

All the variations to the measured signal, apart from that of the analyte, can be considered in the form of error terms:

$$\sum_{i=B}^{N} S_{Ai} x_i = e_i \tag{7a}$$

$$\sum_{j=1}^{m} I_{Aj} x_j = e_j \tag{7b}$$

$$\sum_{k=1}^{p} u_k = e_k \tag{7c}$$

Where e_i + e_j = e_{ij} (the intra-laboratory variations) and e_k (the inter-laboratory variations), Eqn. (2) can be written

$$y_A = y_{A0} + S_{AA}x_A + e_{ij} + e_k + e_A \qquad (8)$$

- **Test of robustness**

Robustness, as defined herein, is an **intra-**laboratory property. In this case, Eqn. (8) reduces to:

$$y_A = y_{A0} + S_{AA}x_A + e_{ij} + e_A \qquad (9)$$

because inter-laboratory effects e_k are not relevant. **Robustness** can be tested in three ways:

(i) in overall terms: as usual by an *F*-test (null hypothesis $H_0 : \sigma_{total} = \sigma_A$ and therefore $H_0^{'} : \sigma_{ij} = 0$):

$$\hat{F} = \frac{s_{total}^2}{s_A^2} = \frac{s_{ijA}^2}{s_A^2} = \frac{s_{ij}^2 + s_A^2}{s_A^2} \qquad (10)$$

If $\hat{F} \le F(\alpha, \nu_1, \nu_2)$, then the null hypothesis cannot be rejected and the procedure can be considered to be robust[2].

(ii) also in general, by means of a Student´s *t*-test (null hypothesis $H_0 : S_{AA}^{real} = S_{AA}^{ideal}$):

$$\hat{t} = \frac{\left|S_{AA}^{real} - S_{AA}^{ideal}\right|}{s_{S_{AA}}\, t_{\alpha,\nu}} \qquad (11)$$

S_{AA}^{real} is the real sensitivity influenced by the sum of cross sensitivities S_{Ai} and the influence strengths I_{Aj}, namely $S_{AA}^{real} = S_{AA}^{ideal} + \sum S_{Ai} + \sum I_{Aj}$. If $\hat{t} > t(\alpha, \nu)$ then a nonlinear error is proved (the real sensitivity differs significantly from the ideal sensitivity) and, therefore, the procedure is not robust. Linear errors are checked as shown in (i) according to Eqn. (10).

(iii) In more detail and individually for each factor (interferent i and influence factor j) the influences can be tested by means of multifactorial experiments where each factor is usually varied at 2 levels. The evaluation of such a multifactorial design is done according to the literature [14, 60-62] where each coefficient of cross sensitivity can be tested separately:

---

[2] α is the risk of error and characterizes the significance level of the test, ν-values stand for the statistical degrees of freedom

$$\hat{t} = \frac{|S_{Ai}|}{s_A \ t_{\alpha,\nu}} \qquad (12)$$

If $|S_{Ai}|$ exceeds the confidence interval $s_A \ t_{\alpha,\nu}$ of the experimental error (analytical error) e_A then the influence of the factor concerned is significant and robustness against this factor is missing. On the other hand, $|S_{Ai}| < s_A t_{\alpha,\nu}$ shows robustness against the particular interferent or factor, respectively. The same discussion applies to the $|I_{Aj}|$-coefficients.

- **Test of ruggedness**

Ruggedness is regarded as an **inter-**laboratory property. In this case, all the terms in Eqn. (8) are relevant ($y_A = y_{A0} + S_{AA}x_A + e_{ij} + e_k + e_A$) and ruggedness can be tested similar to robustness in the same three ways

(iv) in overall terms: as usual by *F*-test (null hypothesis $H_0 : \sigma_{total} = \sigma_A$ and therefore $H_0' : \sigma_{ijkl} = 0$):

$$\hat{F} = \frac{s_{total}^2}{s_A^2} = \frac{s_{ijkA}^2}{s_A^2} = \frac{s_{ij}^2 + s_k^2 + s_A^2}{s_A^2} \qquad (13)$$

The total error s_{ijkA}^2 has to be calculated in different way compared with robustness. Whereas in (i) σ_{ijA}^2 is the variance within a laboratory, s_{ijkA}^2 is the variance between laboratories plus that within the labs, $s_{ijkA}^2 = s_{ijA}^2 + s_k^2 = s_A^2 + s_{ij}^2 + s_k^2$. The interpretation is similar: if $\hat{F} \le F(\alpha, \nu_1, \nu_2)$, then the null hypothesis can not be rejected and the procedure can be considered as to be rugged.

(v) As for robustness in (ii), ruggedness can be tested for nonlinear errors by means of Student´s *t*-test using the same null hypothesis according to Eqn. (11). The interpretation with regard to nonlinear errors is the same as in (ii). Linear errors are checked as shown in (iv) according to Eqn. (13). Only the intra-laboratory effects, and therefore robustness can be studied according to (iii) and tested by Eqn. (12).

Examples of the numerical calculation of **robustness, relative robustness, ruggedness** and **relative ruggedness** are shown in Appendix I.

Conclusions

From a review of the literature the need to distinguish between the use of the terms **robust** and **rugged** and their associated characteristics of **robustness** and **ruggedness** became apparent. Unambiguous definitions have been developed for each term and its associated characteristic, stated as follows:

Robust and Robustness:

A **robust analytical method** is one which exhibits a high degree of robustness following an intra-laboratory study.

The **robustness** of an analytical method is the property that indicates insensitivity against changes of known operational parameters on the results of the method and hence it suitability for a defined purpose.

Rugged and Ruggedness:

A **rugged analytical method** is one that exhibits a high degree of ruggedness after inter-laboratory experiment**.**

The **ruggedness** of an analytical method is the property that indicates insensitivity against changes of known operational variables and in addition any variables (not discovered in intra-laboratory experiments) which may be revealed by inter-laboratory studies.

Furthermore it has been found possible to give quantitative expression to charateristics of **robustness** and **ruggedness** by the introduction of the new concepts of **relative robustness** and **relative ruggedness**, defined as follows:

The **relative robustness** of an analytical method is defined as the ratio of the ideal signal for an uninfluenced method compared to the signal for a method subject to known and unknown operational parameters as studied in an intra-laboratory experiment.

The **relative ruggedness** of an analytical method is defined as the ratio of the ideal signal for an uninfluenced method compared to the signal for a method subject to known and unkown operational parameters as studied in an inter-laboratory experiment.

Acknowledgement

The authors wish to thank IUPAC for their support during the preparation of this paper.

References

Publications in which robust/robustness and rugged/ruggedness are not clearly distinguished

1. M. Sargent and G. MacKay (eds.), "Guidelines for Achieving Quality in Trace Analysis", RSC/LGC/VAM, Cambridge, 1995.
2. M. M. W. B. Hendricks, J. H. de Boer an. K. Smilde, "Robustness of analytical chemical methods and pharmaceutical technological products", Elsevier, Amsterdam, 1996.
3. E. Pritchard, G. M. MacKay and J. Points, "Trace Analysis: A structured approach to obtaining reliable results", RSC/LGC/VAM, Cambridge, 1996.
4. C. M. Riley and T. W. Rosanske, "Development and Validation of Analytical Methods", Pergamon, Oxford, 1996.
5. 5. R. Kellner, J.–M. Mermet, M. Otto and H. M. Widmer (eds.), "Analytical Chemistry", Wiley-VCH, Weinheim, 1998. Note the terms were distinguished in the 2nd. Edition, [49] below.
6. CITAC/EURACHEM Guide, "The Fitness for Purpose of Analytical Methods", 1998.
7. P. Quevauviller, E. A. Maier, "Interlaboratory Studies and Certified Reference Materials for Environmental Analysis. The BCR Approach", Elsevier, Amsterdam, 1999.
8. C. Burgess, "Valid Analytical Measurements and Procedures", RSC, Cambridge, 2000.
9. G. Currell, "Analytical Instrumentation, Performance Characteristics and Quality", J. Wiley, Chichester, 2000.
10. A. Fajgeli and Ā. Ambrus (eds.), "Principles and Practices of Method Validation", RSC, Cambridge, 2000.
11. D. C. Harris, "Quantitative Chemical Analysis", 6th. Edn., Freeman, New York, 2002.
12. CITAC/Eurachem, "Guide to Quality in Analytical Chemistry. An Aid to Accreditation", Edition 2002.
13. P. Meier and R. E. Zünd, "Statistical Methods in Analytical Chemistry", 2nd. Edn., Wiley, New York, 2000.
14. United States Department of Agriculture, "A2LA Food Chemistry Program Requirements", 2001.
15. E. Pritchard, "Analytical Measurement Terminology", RSC/LGC/VAM, Cambridge, 2001.
16. Y. Van der Heyden, A. Nijhuis, J. Smeyers-Verbeke, B.G.M. Vandeginste, D.L. Massart, J. Pharmac. Biomed. Anal. 24 (2001) 723.
17. P. Roper, S. Burke, R. Lawn, V. Barwick and R. Walker, "Applications of Reference Materials in Analytical Chemistry", RSC, Cambridge, 2001.
18. HSE Advisory Committee on Pesticides, "Guidelines for Validation of Analytical Methods for Non-agricultural Pesticide Active Ingredients and Products", 2002.
19. S. Cathum, C.E. Brown and W. Wong, Anal. Bioanal. Chem., 373 (2002) 103.
20. E. Mullins, "Statistics for the Quality Control Chemistry Laboratory", RSC, Cambridge, 2003.
21. J. W. Robinson, E. M. S. Frame and G. M. Frame III, "Undergraduate Instrumental Analysis", 6th. Edn., Marcel Dekker, New York, 2005.
22. AOAC, "Terms and Definitions", AOAC International, Gaithersberg, 2008.

Publications which use robust and robustness only

23. International Conference on Harmonization, ICH, "ICH topic Q 2 A. Validation of Analytical Methods: Definitions and Terminology", (CPMP/ICH/381/95), 1994.
24. International Conference on Harmonisation, ICH, "ICH Topic Q 2B. Validation of Analytical Procedures: Methodology", (CPMP/ICH/281), 1996. This note was incorporated into the parent document , renamed as "Validation of Analytical Procedures: Text and Methodology", Q2 (R1), November 2005.
25. G. Wünsch, J. Prakt. Chemie., 336 (1994) 319.
26. Center for Drug Evaluation and Research, "Reviewer Guidance. Validation of Chromatographic Methods", 1994.

27. FDA, Federal Register, 60, No. 40, 11260, (1995).[Docket No. 94D-0016].
28. E. J. Newman (ed.), "Quality in the Analytical Laboratory", Wiley, 1995. Reprinted 1997.
29. J. M. Green, Anal. Chem., 68 (1996) 305A.
30. H. Fabre, J. Pharm. Biomed. Anal., 14 (1996) 1125.
31. H. Wilder and G. Wünch, J. Prakt. Chem., 339 (1997) 107.
32. M. Valcárcel, "Principles of Analytical Chemistry", Springer, Berlin, 2000.
33. U. S. Department of Health and Human Services, "Guidance for Industry. Analytical Procedures and Method Validation", 2000.
34. M. B. Sanz, L. A. Sarabia, A. Herrero and M. C. Ortiz, Talanta, 56 (2002) 1039.
35. I. García, L. Sarabia, M. C. Ortiz and J. M. Aldama, Analyst, 129 (2004) 766.
36. C.C. Chan, H. Lam, Y. C. Lee and X-M. Zhang, "Analytical Method Validation and Instrument Performance Verification", Wiley-Intercience, Hoboken, 2004.
37. BS EN ISO/IEC 17025:2005, "General requirements for the competence of testing and calibration laboratories", BSI, London, 2005.
38. "British Pharmacopeia 2008", Stationary Office, London, 2008.

Publications which use rugged/ruggedness only

39. W. J. Youden and E. H. Steiner, "Statistical Manual of the Association of Official Analytical Chemists", AOAC, Arlington, 1975.
40. G. Wernimont, ASTM Standarization News, (March) (1977) 13.
41. G. T. Wernimont (W. Spendley ed.), "Use of Statistics to develop and Evaluate Analytical Methods", AOAC, Arlington, 1985. 5th. Reprint 1996.
42. J. P. Dux, "Handbook of Quality Assurance for the Analytical Chemistry Laboratory", Van Nostrand Reinhold, New York, 1986.
43. M. Mulholland, Trends Anal. Chem., 7, (1988) 383.
44. J. A. Leeuwen, L. M. C. Buydens, B. G. M. Vandeginste, G. Kateman, A. Cleland, M. Mulholland, C. Jansen, F. A. Maris, P. H. Hoogkamer and J. H. M. van den Berg, Chemo. Intell. Lab. Syst., 11 (1991) 161.
45. G. Kateman and L. Buydens, "Quality Control in Analytical Chemistry", 2nd. ed., Wiley, New York, 1993.
46. L. A. Currie, Pure Appl. Chem., 67 (1995) 1699.
47. W. Wegscheider, "Validation of Analytical Methods" in: H. Gunzler (ed.), G. Lapitajs (trans.), "Accreditation and Quality Assurance in Analytical Chemistry", Springer, Berlin, 1996.
48. A. Fajgelj ad A. Ambrus, "Principles and Practices of Method Validation", RSC, Cambridge,(2000)
49. ASTM, D 3670-91(Reapproved 2007), "Standard Guide for Determination of Precision and Bias of Methods of Committee D-22", ASTM, West Conshocken, 2007.
50. ASTM, E 1169-89(Reappoved 2007), "Standard Guide for Conducting Ruggedness Tests", ASTM, West Conshocken, 2007.
51. P. Roper, S. Burke, R. Lawn, V. Barwick and R. Walker, "Applications of Reference Materials in Analytical Chemistry", RSC/LGC/VAM, Cambridge, 2001).
52. CEC, "Implementing Council Directive 96/23/EC concerning the performance of analytical methods and the interpretation of results", 2002.
53. M. Thompson, S. L. R. Ellison and R. Wood, Pure Appl. Chem., 74 (2002) 835.
54. 54. LGC, "In-House Method Validation: A Guide for Chemical Laboratories", LGC Ltd., Teddington, 2003.
55. K. Danzer, Anal. Bioanal. Chem., 380 (2004) 376.
56. E. Pritchard and V. Barwick, "Quality Assurance in Analytical Chemistry", J. Wiley, Chichester, 2007.
57. J. Inczédy, "Kémiai Folyamatok Műszeres Ellenőrzése", Veszprémi Egyetemi Kiadó, Veszprém, (2001).

Publications which distinguish between robust/robustness and rugged/ruggedness

58. J. C. Wahlich and G. P.Carr, J. Pharm. Biomed. Anal., 8 (1990) 619.
59. "The United States Pharmacopoeia, The National Formulary", United States Pharmacopeial Convention Inc., Rockville, 1995 and 2000.

60. C. M. Riley and T. W. Rosanske, “Development and Validation of Analytical Methods”, Pergamon, Oxford, 1996.
61. L. C. Rodŕiguez, R. B. García, A. M. García Campaña and J. M. B. Sendra, Chemo. Intell. Lab. Syst., 41 (1998) 57.
62. I. S. Krull and M. Swartz, Anal. Lett., 32 (1999) 1067.
63. M. Zeaiter, J. -M. Roger, V. Bellon-Maurel and D. N. Rutledge, Trends Anal. Chem., 23 (2004) 157.
64. J. –M. Mermet , M. Otto, M. Valcárcel (Eds.), R. Kellner and H. M. Widmer (Founding Eds.), “Analytical Chemistry: A Modern Approach to Analytical Science”, Wiley-VCH, Weinheim, 2004.
65. J. Ermer and J. H. McB. Miller, “Method Validation in Pharmaceutical Practice: A Guide to Best Practice”, Wiley-VCH, Weinheim, 2005.
66. L. Cuadros-Rodriguez, R. Romero and J. M. Bosque-Sendra, Crit. Rev. Anal. Chem., 35 (2005) 57.
67. K. Danzer, “Analytical Chemistry: Theoretical and Methodological Fundamentals”, Springer,Heidelberg, 2007.
68. S. Chandran and R. S. P. Singh, Pharmazie, 62 (2007) 1.

Use of robust statistics and rugged experimental design

69. J. C. Miller and J. N. Miller, "Statistics and Chemometrics for Analytical Chemistry", 5th. Edn., Pearson Prentice Hall, Harlow, 2005.

Publications which use the term robust/robustness in connection with statistics

70. W. J. J. Rey, "Robust Statistical Methods", Springer-Verlag, Berlin, 1978.
71. R. L. Launer and G. N. Wilkinson, "Robustness in Statistics", Academic Press, New York, 1979.
72. P. J. Huber, "Robust Statistics", Wiley, Chichester, 1981.
73. G. E. P. Box, T. Leonard and C. Wu (eds.), "Scientific inference, data analysis and robustness", Academic Press, New York, 1983.
74. D. C. Hoaglin, F. Mosteller and J. W. Tukey, "Understanding Robust and Exploratory Data Analysis", Wiley, New York, 1983.
75. Analytical Methods Committee, Analyst, 114 (1989) 1693, 1699.
76. R. E. Lawn, M. Thompson and R. F. Walker, "Proficiency Testing in Analytical Chemistry", RSC/LGC/VAM, 1997.
77. Analytical Methods Committee, AMC Technical Brief No. 6, "Robust statistics: a method of coping with outliers", RSC, 2001.

Qualification of Analytical Instruments

78. S. K. Bansal, T. Layloff, E. D. Bush, M. Hamilton, E. A. Hankinson, J. S. Landy, S. Lowes, M. N. Nasr, P. A. St. Jean and V. P. Shah, AAPS PharmSciTech., 5(1) 2004 Article 22, [http://www.aapspharmscitech.org]

Appendix I
Examples of the Numerical Evaluation of Robustness and Ruggedness

Robustness Example

Determination of analyte *A* in presence of *B* and *C* under influence of the factors f_1 , f_2 , f_3.

Input data:	Sensitivity of *A*	S_{AA} = 10		
	Content of *A*	x_A = 1		
	Cross sensitivities:	S_{AB} = 1	S_{AC} = 1	
	Influence strengths:	I_{A1} = 2	I_{A2} = –1	I_{A3} = 1
	Experimental error:	s_A = 1		
	Degrees of freedom:	ν = 10		

Robustness will be computed for various amounts of *B* and *C* as well as for various values of f_1 , f_2 , f_3.

a)

Strong matrix interferences	Strong factor influences
$x_B = x_C = 1$	$x_1 = x_2 = x_3 = 1$

$$rob(A/B,C;\, f_1, f_2, f_3) = \frac{1}{\sum_{i=A}^{B} |S_{Ai}| x_i + \sum_{j=1}^{3} |I_{Aj}| x_j} = \frac{1}{1+1+2+1+1} = \frac{1}{6} = 0.167$$

$$rob_{rel}(A/B,C;\, f_1, f_2, f_3) = \frac{S_{AA} x_A}{S_{AA} x_A + \sum_{i=A}^{B} |S_{Ai}| x_i + \sum_{j=1}^{3} |I_{Aj}| x_j} = \frac{10}{10+6} = \frac{10}{16} = 0.625$$

Thus the relative robustness is only 62.5 %.

- Test for robustness by *F*-test (i)

$$\hat{F} = \frac{variance\ of\ total\ variations}{variance\ of\ experimental\ error} = \frac{\sigma_{ijA}^2}{\sigma_A^2} = \frac{1+1+4+1+1+1}{1} = 9 \quad > \quad F(95;10;10) = 2.79$$

Hence the procedure is **not robust** under the given conditions.

- Test for nonlinear errors by Student’s t test (ii)

$$\hat{t} = \frac{\left|S_{AA}^{real} - S_{AA}^{ideal}\right|}{s_{S_{AA}} t_{\alpha,\nu}}$$

where

$$S_{AA}^{real} = S_{AA} + \sum_{i=B}^{C} S_{Ai} \frac{x_i}{x_A} + \sum_{j=1}^{3} I_{Aj} \frac{x_j}{x_A}$$ is the **real sensitivity**

$$S_{AA}^{real} = 10+1+1+2-1+1 = 14$$

$$\hat{t} = \frac{14-10}{1+2.23} = \frac{4}{2.23} = 1.79 \quad < \quad t(95;10) = 2.23$$

Hence there is **no significant nonlinear error** under the given conditions.

- Test (iii) for each individual factor only makes sense if multi-factorial experiments are planned and evaluated. It is too difficult to construct such a design and evaluate it herein.

b)

Low matrix interferences	Strong factor influences
$x_B = x_C = 0.01$	$x_1 = x_2 = x_3 = 1$

$$rob(A/B,C; f_1, f_2, f_3) = \frac{1}{0.01+0.01+2+1+1} = \frac{1}{4.02} = 0.249$$

$$rob_{rel}(A/B,C; f_1, f_2, f_3) = \frac{10}{10+4.02} = \frac{10}{14.02} = 0.713$$

Thus the relative robustness is only 71.3 %.

- Test for robustness by *F*-test (i)

$$\hat{F} = \frac{2 \cdot 0.0001+4+1+1+1}{1} = 7.0002 \quad > \quad F(95;10;10) = 2.79$$

Hence the procedure is **not robust** under the given conditions.

- Test for nonlinear errors by Student's *t*-test (ii)

$$S_{AA}^{real} = 10+0.01+0.01+2-1+1 = 12.02$$

$$\hat{t} = \frac{12.02 - 10}{2.23} = \frac{2.02}{2.23} = 0.906 \quad < \quad t(95;10) = 2.23$$ ([3])

Hence there is **no significant nonlinear error** under the given conditions.

c)

Strong matrix interferences	Low factor influences
$x_B = x_C = 1$	$x_1 = x_2 = x_3 = 0.01$

$$rob(A/B,C; f_1, f_2, f_3) = \frac{1}{1+1+0.02+0.01+0.01} = \frac{1}{2.04} = 0.490$$

$$rob_{rel}(A/B,C; f_1, f_2, f_3) = \frac{10}{10+2.04} = \frac{10}{12.04} = 0.831$$

Thus the relative robustness is 83.1 %.

- Test for robustness by the *F*-test (i)

$$\hat{F} = \frac{1+1+0.0004+2\cdot 0.0001+1}{1} = 3.0006 \quad > \quad F(95;10;10) = 2.79$$

Hence the procedure is **not robust** under the given conditions

- Test for nonlinear errors by Student's *t*-test (ii)

$$S_{AA}^{real} = 10+1+1+0.02-0.01+0.01 = 12.02$$

$$\hat{t} = \frac{12.02 - 10}{2.23} = \frac{2.02}{2.23} = 0.906 \quad < \quad t(95;10) = 2.23$$

Hence there is **no significant nonlinear error** under the given conditions.

d)

Low matrix interferences	Low factor influences
$x_B = x_C = 0.01$	$x_1 = x_2 = x_3 = 0.01$

[3] Different from the usual procedure, the lower variance has to be in numerator for factual reasons

$$rob(A/B,C;f_1,f_2,f_3)=\frac{1}{0.01+0.01+0.02+0.01+0.01}=\frac{1}{0.06}=16.667$$

$$rob_{rel}(A/B,C;f_1,f_2,f_3)=\frac{10}{10+0.06}=\frac{10}{10.06}=0.994$$

Thus the relative robustness is 99.4 %.

- Test for robustness by the F-test (i)

$$\hat{F}=\frac{2\cdot 0.0001+0.0004+2\cdot 0.0001+1}{1}=1.0008 \quad < \quad F(95;10;10)=2.79$$

Hence the procedure is **robust** under the given conditions.

- Test for nonlinear errors by the Student's t-test (ii)

$$S_{AA}^{real}=10+0.01+0.01+0.02-0.01+0.01=12.04$$

$$\hat{t}=\frac{10.04-10}{2.23}=\frac{0.04}{2.23}=0.018 \quad < \quad t(95;10)=2.23 \text{ (}^4\text{)}.$$

Hence there is **no significant nonlinear error** under the given conditions.

e)

No matrix interferences	Low factor influences
$x_B = x_C = 0$	$x_1 = x_2 = x_3 = 0.01$

$$rob(A/B,C;f_1,f_2,f_3)=\frac{1}{0.02+0.01+0.01}=\frac{1}{0.04}=25.0$$

$$rob_{rel}(A/B,C;f_1,f_2,f_3)=\frac{10}{10+0.04}=\frac{10}{10.04}=0.996$$

Thus the relative robustness is 99.6 %.

Test for robustness (is unnecessary to carry out in view of the relative robustness of 99.6%, but is given here for completeness)

---

[4] Of course, in case of such a small difference t-test is unnecessary

- Test for robustness by the *F*-test (i)

$$\hat{F} = \frac{0.0004 + 2 \cdot 0.0001 + 1}{1} = 1.0006 \quad < \quad F(95;10;10) = 2.79$$

that means the method is **robust**.

- Test for nonlinear errors by Student's *t*-test (ii)

$$S_{AA}^{real} = 10 + 0.02 - 0.01 + 0.01 = 10.02 \ ([5])$$

There is **no significant nonlinear error** under the given conditions.

[5] *t*-test is unnecessary

Ruggedness Example

Determination of analyte *A* in three laboratories U, V, W in the context of an inter-laboratory study. Each laboratory should have approximately the same head data (no significant differences) as listed above.

If the experimental errors estimated from the inter-laboratory test were the following:

Lab U: $s_{A_U} = 1.1$	Lab V: $s_{A_V} = 0.9$	Lab W: $s_{A_W} = 1.0$
Error between the labs: $s_{total} = s_{ijkA} = 5.2$		

The degrees of freedom are $\nu_U = \nu_V = \nu_W = 10$, $\nu_{total} = 30$

Robustness computed for every laboratory with the data of case (1.4) yields the same result for each lab:

$$rob(A/B,C;\, f_1,\, f_2,\, f_3) = \frac{1}{0.01+0.01+0.02+0.01+0.01} = \frac{1}{0.06} = 16.667$$

$$rob_{rel}(A/B,C;\, f_1,\, f_2,\, f_3) = \frac{10}{10+0.06} = \frac{10}{10.06} = 0.994$$

Hence the relative robustness is 99.4 % for each lab.

Ruggedness:

$$rug(A/B,C;\, f_1,\, f_2,\, f_3;u) = \frac{1}{0.01+0.01+0.02+0.01+0.01+5.2} = \frac{1}{5.26} = 0.190$$

$$rug_{rel}(A/B,C;\, f_1,\, f_2,\, f_3;u) = \frac{10}{10+0.06+5.2} = \frac{10}{15.26} = 0.655$$

Thus the relative ruggedness is only 65.5 %.

- Test for ruggedness by the F-test (i):

$$\hat{F} = \frac{2\cdot 0.001+0.0004+2\cdot 0.0001+1+5.2}{1} = 6.2006 \quad > \quad F(95;27;10) = 2.73$$

Hence the inter-laboratory test shows that the procedure is **not rugged** although for each laboratory **robustness** has been achieved.

Robustness and Ruggedness Example

Three laboratories U, V, and W each used the same procedure to determine the analyte *A* in the presence of *B* and *C* under influence of the factors f_1 , f_2 , f_3.

Input data:

		U	V	W
Sensitivity of *A*	S_{AA}	10	8	11
Cross sensitivities	S_{AB}	1.2	1.4	0.9
	S_{AC}	1.5	1.9	1.1
Influence strengths	I_{A1}	0.25	0.22	0.24
	I_{A2}	– 0.13	– 0.14	– 0.12
	I_{A3}	0.08	0.10	0.11
Experimental error	s_A	1.2	1.5	0.8
Degrees of freedom	ν	10	10	10

- Computation of the robustness of the procedure in lab U where the following conditions exist:

x_B	x_C	x_1	x_2	x_3
0.11	0.08	1.5	0.7	1.3

$$rob(A/B,C;f_1,f_2,f_3)=\frac{1}{\sum_{i=A}^{B}|S_{Ai}|x_i+\sum_{j=1}^{3}|I_{Aj}|x_j}=\frac{1}{1.2\cdot 0.11+1.5\cdot 0.08+0.25\cdot 1.5+0.13\cdot 0.7+0.08\cdot 1.3}=\frac{1}{0.822}=1.22$$

$$rob_{rel}(A/B,C;f_1,f_2,f_3)=\frac{S_{AA}x_A}{S_{AA}x_A+\sum_{i=A}^{B}|S_{Ai}|x_i+\sum_{j=1}^{3}|I_{Aj}|x_j}=\frac{10}{10+0.822}=\frac{10}{10.822}=0.924$$

Thus the relative robustness of the lab U is 92.4 %.

Using the *F*-test,

$$\hat{F}=\frac{s_{ijA}^2}{s_A^2}=\frac{0.132^2+0.12^2+0.375^2+0.091^2+0.104^2+1.2^2}{1.2^2}=1.123<F(95;10;10)=2.79$$

Thus the procedure is **robust** in lab U.

- Computation of the robustness of the procedure in lab V where the following conditions existed:

x_B	x_C	x_1	x_2	x_3
0.14	0.13	1.8	1.7	1.6

$$rob(A/B,C; f_1, f_2, f_3) = \frac{1}{1.4\cdot 0.14 + 1.9\cdot 0.13 + 0.22\cdot 1.8 + 0.14\cdot 1.7 + 0.10\cdot 1.6} = \frac{1}{1.237} = 0.808$$

$$rob_{rel}(A/B,C; f_1, f_2, f_3) = \frac{10}{10+1.237} = \frac{10}{11.237} = 0.890$$

Thus the relative robustness of the lab V is 89.0 %.

Using the *F*-test,

$$\hat{F} = \frac{s_{ijA}^2}{s_A^2} = \frac{0.196^2 + 0.247^2 + 0.396^2 + 0.238^2 + 0.160^2 + 1.5^2}{1.5^2} = 1.15 < F(95;10;10) = 2.79$$

Hence the method is **robust** in lab V

- Computation of the robustness of the procedure in lab W where the following conditions existed:

x_B	x_C	x_1	x_2	x_3
0.19	0.23	0.8	1.1	1.4

$$rob(A/B,C; f_1, f_2, f_3) = \frac{1}{0.9\cdot 0.19 + 1.1\cdot 0.23 + 0.24\cdot 0.8 + 0.12\cdot 1.1 + 0.11\cdot 1.4} = \frac{1}{0.902} = 1.109$$

$$rob_{rel}(A/B,C; f_1, f_2, f_3) = \frac{10}{10+0.902} = \frac{10}{10{,}902} = 0.917$$

Thus the relative robustness of the lab. W is 91.7 %.

Using the *F*-test,

$$\hat{F} = \frac{s_{ijA}^2}{s_A^2} = \frac{0.171^2 + 0.253^2 + 0.192^2 + 0.132^2 + 0.154^2 + 0.8^2}{0.8^2} = 1.27 < F(95;10;10) = 2.79$$

Hence the method is **robust** in labW

- Ruggedness

For the calculation of $rug(A/B,C; f_1,f_2,f_3;u)$ the individual error terms have been averaged[6]:

$$s_{ij}^2 = \tfrac{1}{3}(s_{ij_U}^2 + s_{ij_V}^2 + s_{ij_W}^2) = \tfrac{1}{3}(0.183 + 0.338 + 0.172) = 0.231$$

[6] This is possible because they do not differ significantly from each other (Hartley-test)

$$s_A^2 = \tfrac{1}{3}(s_{A_U}^2 + s_{A_V}^2 + s_{A_W}^2) = \tfrac{1}{3}(1.44 + 2.25 + 0.64) = 1.44$$

This yields $s_{ijA} = \sqrt{s_{ij}^2 + s_A^2} = 1.293$

$$s_{total} = s_{ijkA} = 5.2$$

$$rug(A/B,C;f_1,f_2,f_3;u) = \frac{1}{\sum_{i=B}^{C}|S_{Ai}|x_i + \sum_{j=1}^{3}|I_{Aj}|x_j + u} = \frac{1}{\sqrt{s_{total}^2}} = \frac{1}{\sqrt{s_{ij}^2 + s_A^2 + s_{between}^2}} = \frac{1}{\sqrt{s_{ij}^2 + s_A^2 + s_u^2}}$$

$rug(A/B,C; f_1,f_2,f_3;u)$ = 1/5.2 = 0.192

$rug_{rel}(A/B,C; f_1,f_2,f_3;u) = \dfrac{\bar{S}_{AA}}{\bar{S}_{AA} + s_{total}} = \dfrac{9.667}{14.867} = 0.650$ ($\bar{S}_{AA} = 9.667$)

Thus the relative ruggedness is 65.0 %.

Using the F-test,

$$\hat{F} = \frac{s_{total}^2}{s_A^2} = \frac{5.2^2}{1.2^2} = 18.778 > F(95;27;27) = 1.86$$

Hence **no ruggedness** existed

Although for each lab robustness has been found, ruggedness cannot be stated to exist in this given case. To establish ruggedness it would be essential that the causes for the large interlaboratory deviations ("the unknowns") be found and eliminated.
