# Supplementary material for: Stability-Indicating RP-HPLC Determination of Acetylsalicylic Acid, Dextromethorphan Hydrobromide, Phenylephrine Bitartrate, and Chlorpheniramine Maleate in an Effervescent Tablet Formulation
Source: J AOAC Int. 2026 Aug 7;109(6):887–96. doi: 10.1093/jaoacint/qsag070 (PMC13732704; doi:10.1093/jaoacint/qsag070)
Supplement: Supplementary file 1 [file qsag070_supplementary_data.zip › qsag070_Supplementary_Data/aoac-25-0299-File011.pdf]

INTERNATIONAL COUNCIL FOR HARMONISATION OF TECHNICAL REQUIREMENTS FOR PHARMACEUTICALS FOR HUMAN USE

ICH HARMONISED GUIDELINE

VALIDATION OF ANALYTICAL PROCEDURES
Q2(R2)

Draft version

Endorsed on 24 March 2022

Currently under public consultation

At Step 2 of the ICH Process, a consensus draft text or guideline, agreed by the appropriate ICH Expert Working Group, is transmitted by the ICH Assembly to the regulatory authorities of the ICH regions for internal and external consultation, according to national or regional procedures.

Q2(R2)
Document History

Code	History	Date
Q2	Approval by the Steering Committee under Step 2 and release for public consultation.	26 October 1993
Q2A	Approval by the Steering Committee under Step 4 and recommendation for adoption to the three ICH regulatory bodies.	27 October 1994
Q2B	Approval by the Steering Committee under Step 2 and release for public consultation.	29 November 1995
Q2B	Approval by the Steering Committee under Step 4 and recommendation for adoption to the three ICH regulatory bodies.	6 November 1996
Q2(R1)	The parent guideline is now renamed Q2(R1) as the guideline Q2B on methodology has been incorporated to the parent guideline. The new title is "Validation of Analytical Procedures: Text and Methodology".	November 2005
Q2(R2)	Complete revision of guideline to include more recent application of analytical procedures and to align content with *Q14*.<br>Endorsement by the Members of the ICH Assembly under *Step 2* and release for public consultation.	24 March 2022

Legal notice: *This document is protected by copyright and may, with the exception of the ICH logo, be used, reproduced, incorporated into other works, adapted, modified, translated or distributed under a public license provided that ICH's copyright in the document is acknowledged at all times. In case of any adaption, modification or translation of the document, reasonable steps must be taken to clearly label, demarcate or otherwise identify that changes were made to or based on the original document. Any impression that the adaption, modification or translation of the original document is endorsed or sponsored by the ICH must be avoided.*
The document is provided "as is" without warranty of any kind. In no event shall the ICH or the authors of the original document be liable for any claim, damages or other liability arising from the use of the document.
The above-mentioned permissions do not apply to content supplied by third parties. Therefore, for documents where the copyright vests in a third party, permission for reproduction must be obtained from this copyright holder.

ICH HARMONISED GUIDELINE

VALIDATION OF ANALYTICAL PROCEDURES

Q2(R2)

ICH Consensus Guideline

TABLE OF CONTENTS

1 INTRODUCTION 1

2 SCOPE 1

3 ANALYTICAL PROCEDURE VALIDATION STUDY 2

3.1 Validation during the lifecycle of an analytical procedure 4

3.2 Reportable Range 5

3.3 Demonstration of stability indicating properties 5

3.4 Considerations for multivariate analytical procedures 6

3.4.1 Reference analytical procedure(s) 6

4 VALIDATION TESTS, METHODOLOGY AND EVALUATION 7

4.1 Specificity / Selectivity 7

4.1.1 Absence of interference 7

4.1.2 Orthogonal procedure comparison 7

4.1.3 Technology inherent justification 7

4.1.4 Recommended Data 7

4.1.4.1 Identification 7

4.1.4.2 Assay, purity- and impurity test(s) 8

4.2 Working Range 9

4.2.1 Response 9

4.2.1.1 Linear Response 9

4.2.1.2 Non-linear Response 9

4.2.1.3 Multivariate calibration 10

4.2.2 Validation of lower range limits 10

4.2.2.1 Based on signal-to-noise 10

4.2.2.2 Based on the Standard Deviation of a Linear Response and a Slope 11

4.2.2.3 Based on Accuracy and Precision at lower range limits 11

4.2.2.4 Recommended Data 11

4.3 Accuracy and Precision 12

4.3.1 Accuracy 12

4.3.1.1 Reference material comparison 12

4.3.1.2 Spiking Study 12
4.3.1.3 Orthogonal Procedure comparison 12
4.3.1.4 Recommended Data 13
4.3.2 Precision 13
4.3.2.1 Repeatability 13
4.3.2.2 Intermediate Precision 14
4.3.2.3 Reproducibility 14
4.3.2.4 Recommended Data 14
4.3.3 Combined approaches for accuracy and precision 14
4.3.3.1 Recommended Data 15
4.4 Robustness 15
5 GLOSSARY 16
6 REFERENCES 22
7 ANNEX 1 SELECTION OF VALIDATION TESTS 23
8 ANNEX 2 ILLUSTRATIVE EXAMPLES FOR ANALYTICAL TECHNIQUES 24

1 INTRODUCTION

This guideline presents a discussion of elements for consideration during the validation of *analytical procedures* included as part of registration applications submitted within the ICH member regulatory authorities. Q2(R2) provides guidance and recommendations on how to derive and evaluate the various *validation tests* for each analytical procedure**.** This guideline serves as a collection of terms, and their definitions. These terms and definitions are meant to bridge the differences that often exist between various compendia and documents of the ICH member regulatory agencies.

The objective of validation of an analytical procedure is to demonstrate that the analytical procedure is suitable for the intended purpose. A tabular summary of the characteristics applicable to common types of uses of analytical procedures is included (Table 1). Further general guidance is provided on how to perform *validation studies* for analytical procedures.

The document provides an indication of the data which should be presented in a regulatory submission. Analytical procedure validation data should be submitted in the corresponding sections of the application in the ICH M4Q THE COMMON TECHNICAL DOCUMENT FOR THE REGISTRATION OF PHARMACEUTICALS FOR HUMAN USE. All relevant data collected during validation (and any methodology used for calculating validation results) should be submitted to establish the suitability of the procedure for the intended use. Of note, suitable data derived from development studies (see ICH Q14) can be used in lieu of validation data. When an established *platform analytical procedure* is used for a new purpose, validation testing can be abbreviated, if scientifically justified.

Approaches other than those set forth in this guideline may be applicable and acceptable with appropriate science-based justification. The applicant is responsible for designing the validation studies and protocol most suitable for their product.

Suitably characterized reference materials, with documented identity and purity or any other characteristics as necessary, should be used throughout the validation study. The degree of purity necessary for the reference material depends on the intended use.

In practice, the experimental work can be designed so that the appropriate validation tests can be performed to provide sound, overall knowledge of the performance of the analytical procedure, for instance: *specificity/selectivity*, *accuracy,* and *precision over the reportable range*.

As described in *ICH Q14*, the *system suitability test (SST)* is an integral part of analytical procedures and is generally established during development as a regular check of performance. *Robustness* typically should be evaluated as part of development prior to the execution of the analytical procedure validation study (ICH Q14).

2 SCOPE

This guideline applies to new or revised analytical procedures used for release and stability

testing of commercial drug substances and products (chemical and biological/biotechnological). The guideline can also be applied to other analytical procedures used as part of the control strategy (*ICH Q8-Q10*) following a risk-based approach. The scientific principles described in this guideline can be applied in a phase-appropriate manner during clinical development. This guideline may also be applicable to other types of products, with appropriate regulatory authority consultation as needed.

The guideline is directed to the most common purposes of analytical procedures, such as assay/potency, purity, impurity (quantitative or limit test), identity or other quantitative or qualitative measurements.

3 ANALYTICAL PROCEDURE VALIDATION STUDY

A validation study is designed to provide sufficient evidence that the analytical procedure meets its objectives. These objectives are described with a suitable set of *performance characteristics* and related *performance criteria*, which can vary depending on the intended use of the analytical procedure and the specific technology selected. The section "VALIDATION TESTS, METHODOLOGY AND EVALUATION" summarizes the typical methodology and validation tests that can be used (see flowchart in Annex 1). Specific non-binding examples for common techniques are given in Annex 2. Based on Annex 1 and the measured product attributes, typical performance characteristics and related validation tests are provided in Table 1.

Table 1: Typical performance characteristics and related validation tests for measured product attributes

Type of measured product attribute / Analytical Procedure Performance Characteristics to be demonstrated (2)	IDENTITY	IMPURITY (PURITY) Other quantitative measurements (1)		ASSAY content/potency Other quantitative measurements (1)
		Quantitative	Limit	
Specificity (3)				
Specificity Test	+	+	+	+
Working Range				
Suitability of Calibration model	-	+	-	+
Lower Range Limit verification	-	QL (DL)	DL	-
Accuracy (4)				
Accuracy Test	-	+	-	+
Precision (4)				
Repeatability Test	-	+	-	+
Intermediate Precision Test	-	+ (5)	-	+ (5)

- signifies that this test is not normally evaluated

+ signifies that this test is normally evaluated

( ) signifies that this test is normally not evaluated, but in some complex cases recommended

QL, DL: Quantitation Limit, Detection Limit

(1) other quantitative measurements can follow the scheme of impurity testing, if the working range is close to the detection or quantitation limits of the technology, otherwise following the assay scheme is recommended.

(2) some performance characteristics can be substituted with technology inherent justification or qualification in the case of certain analytical procedures for physicochemical properties.

(3) a combined approach can be used alternatively to evaluating accuracy and precision separately

(4) lack of specificity of one analytical procedure could be compensated by one or more other supporting analytical procedures.

(5) Reproducibility and intermediate precision can be performed as a single set of experiments.

The objective of the analytical procedure, appropriate performance characteristics and associated criteria and appropriate validation tests (including those excluded from the validation protocol) should be documented and justified.

Prior to the validation study, a validation protocol should be generated. The protocol should contain information about the intended purpose of the analytical procedure, and performance characteristics and associated criteria to be validated. In cases where pre-existing knowledge (*e.g.,* from development or previous validation) is used appropriate justification should be provided. The results of the validation study should be summarized in a validation report.

Figure 1 shows how knowledge can be generated during analytical procedure development as described in ICH Q14 and aid the design of a validation study.

Figure 1: Validation study design and evaluation

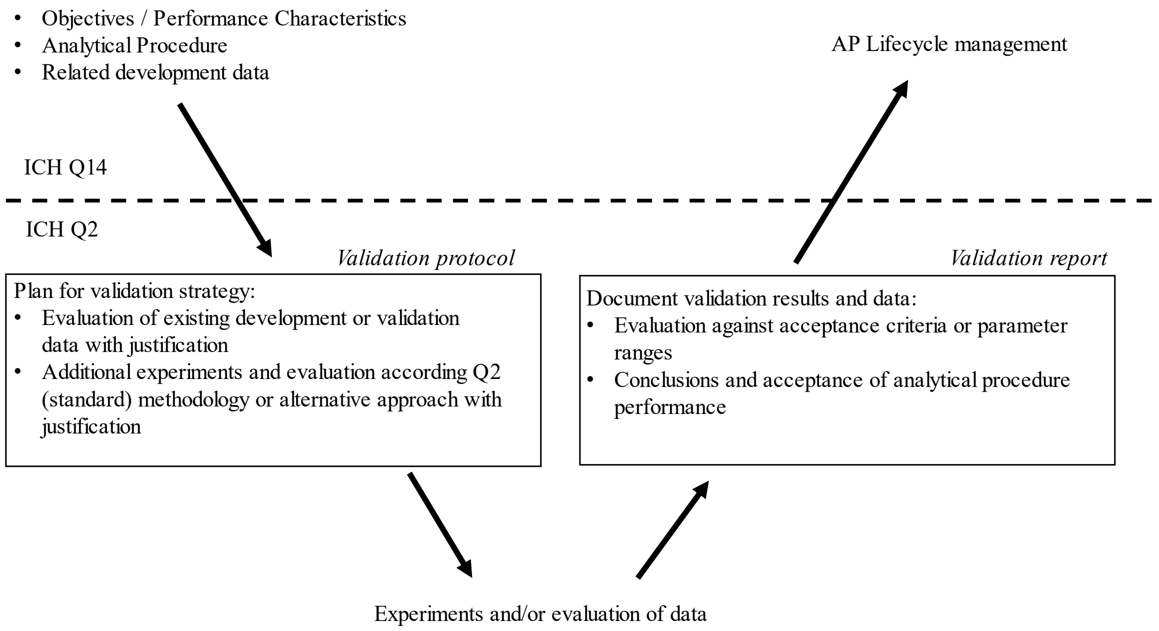

3.1 Validation during the lifecycle of an analytical procedure

Changes may be required during the lifecycle of an analytical procedure. In such cases, partial or full *revalidation* may be required. Science and risk-based principles can be used to justify whether or not a given performance characteristic needs revalidation. The extent of revalidation depends on the analytical performance characteristics impacted by the change.

Co-validation can be used to demonstrate that the analytical procedure meets predefined performance criteria by using data from multiple sites. When transferring analytical procedures to a different laboratory, a subset of validation experiments is often performed.

Cross-validation is an approach which can be used to show that two or more analytical

procedures can be used for the same intended purpose. Cross-validation should demonstrate that the same predefined performance criteria are met for these procedures.

3.2 Reportable Range

The *reportable range* is typically derived from the product specifications and depends on the intended use of the procedure. The reportable range is confirmed by demonstrating that the analytical procedure provides results with acceptable accuracy, precision and specificity. The reportable range should be inclusive of the upper and lower specification or reporting limits, as applicable.

The table below exemplifies recommended reportable ranges for some uses of analytical procedures; other ranges may be acceptable if justified. In some cases, *e.g.,* at low amounts, wider upper ranges may be more practical.

Table 2: Reportable ranges for common uses of analytical procedures

**Use of analytical procedure**	**Low end of reportable range**	**High end of reportable range**
Assay of a drug substance or a finished (drug) product	80% of declared content or 80% of lower specification limit	120% of declared content or 120% of the upper specification limit
Potency	Lowest specification acceptance criterion -20%	Highest specification acceptance criterion +20%
Content uniformity	70% of declared content	130% of declared content
Dissolution testing	Q-45% (immediate release) of the dosage form strength first measurement timepoint or QL (modified release)	130% of declared content of the dosage form
Impurity testing	Reporting threshold	120% of specification limit
Purity testing (as area %)	80% of specification limit	100% of specification limit

3.3 Demonstration of stability indicating properties

If a procedure is a validated quantitative analytical procedure that can detect changes in relevant

quality attributes of a drug substance or drug product during storage, the procedure is considered a stability-indicating test. To demonstrate specificity/selectivity of a stability-indicating test, a combination of challenges should be performed with appropriate justification from development studies. These can include: the use of samples spiked with target analytes and all known interferences; samples that have been exposed to various physical and chemical stress conditions; and actual product samples that are either aged or have been stored at higher temperature and/or humidity.

3.4 Considerations for multivariate analytical procedures

For *multivariate analytical procedures,* results are determined through a multivariate calibration model utilizing more than one input variable (*e.g.,* a spectrum with many wavelength variables). The multivariate calibration model relate the input data to a value for the property of interest (i.e.*,* the model output).

Successful validation of a multivariate procedure should consider calibration, *internal testing* and validation.

Typically, calibration and validation are performed in two phases.

- In the first phase, model development consists of calibration and internal testing. Calibration data are used to create the calibration model. Test data are used for internal testing and optimisation of the model. The test data could be a separate set of data or part of the *calibration data set* used in a rotational manner. This internal test step is used to obtain an estimate of the model performance and to fine-tune an algorithm's parameters (*e.g.,* the number of *latent variables* for partial least squares (PLS)) to select the most suitable model within a given set of data and prerequisites.

- In the second phase, *model validation,* an independent validation data set with *independent samples* is used for validation of the model.

3.4.1 Reference analytical procedure(s)

Samples used for the validation of quantitative or qualitative multivariate procedures require should have values or categories assigned to each sample, typically obtained by a validated procedure or pharmacopeial reference procedure.

When a reference analytical procedure is used, its performance should match the expected performance of the multivariate analytical procedure. Analysis by the *reference procedure* and multivariate data collection should be performed on the same samples (whenever possible), within a reasonable period of time to assure sample and measurement stability. In some cases, a correlation or conversion may be needed to provide the same unit of measure. Any assumptions or calculations should be described.

4 VALIDATION TESTS, METHODOLOGY AND EVALUATION

In the following chapters, experimental methodologies to evaluate the performance of an analytical procedure are described. The methodology described is grouped by the main performance characteristic the analytical procedure was designed for. However, it is acknowledged that information about other performance characteristics may be derived from the same dataset. Other approaches may be used to demonstrate that the analytical procedure meets the objectives and related performance criteria, if justified.

4.1 Specificity / Selectivity

The *specificity* or *selectivity* of an analytical procedure can be demonstrated through absence of interference, comparison of results to an orthogonal procedure or may be inherently given by the underlying scientific principles of the analytical procedure. Some experiments can be combined with accuracy studies.

Selectivity could be demonstrated when the analytical procedure is not specific. However, the test for an analyte to be identified or quantified in the presence of potential interference should minimize that interference and prove that the test is fit for purpose.

Where one analytical procedure does not provide sufficient discrimination, a combination of two or more procedures is recommended to achieve the necessary level of selectivity.

4.1.1 Absence of interference

Specificity/selectivity can be shown by demonstrating that the identification and/or quantitation of an analyte is not impacted by the presence of other substances (*e.g.,* impurities, degradation products, related substances, matrix, or other components present in the operating environment).

4.1.2 Orthogonal procedure comparison

Specificity/selectivity can be verified by demonstrating that the measured result of an analyte is comparable to the measured result of a second, well characterized analytical procedure (*e.g.,* an orthogonal procedure).

4.1.3 Technology inherent justification

In some cases where the specificity of the analytical technology can be ensured and predicted by technical parameters (*e.g.,* resolution of isotopes in mass spectrometry, chemical shifts of NMR signals), no experimental study may be required, if justified.

4.1.4 Recommended Data

4.1.4.1 Identification

For identification tests, a critical aspect is to demonstrate the capability to identify the analyte of interest based on unique aspects of its molecular structure and/or other specific properties.

The capability of an analytical procedure to identify an analyte can be confirmed by obtaining positive results comparable to a known reference material with samples containing the analyte, along with negative results from samples which do not contain the analyte. In addition, the identification test can be applied to materials structurally similar to or closely related to the analyte to confirm that an undesired positive *response* is not obtained. The choice of such potentially interfering materials should be based on scientific judgement with a consideration of any interference that could occur.

4.1.4.2 Assay, purity- and impurity test(s)

The specificity/selectivity of an analytical procedure should be demonstrated to fulfil the accuracy requirements for the content or potency of an analyte in the sample.

Representative data (*e.g.,* chromatograms, electropherograms or spectra) should be used to demonstrate specificity and individual components should be appropriately labelled.

Suitable discrimination should be investigated at an appropriate level (*e.g.,* for critical separations in chromatography, specificity can be demonstrated by the resolution of the two components which elute closest to each other). Alternately, spectra of different components could be compared to assess the possibility of interference.

In case a single procedure is not considered sufficiently selective, an additional procedure should be used to ensure adequate specificity. For example, where a titration is used to assay a drug substance for release, the combination of the assay and a suitable test for impurities can be used.

The approach is similar for both assay and impurity tests:

Impurities or related substances are available:

For assay, discrimination of the analyte in the presence of impurities and/or excipients should be demonstrated. Practically, this can be performed by spiking drug substance or drug product with appropriate levels of impurities and/or excipients and demonstrating that the assay result is unaffected by the presence of these materials (*e.g.,* by comparison with the assay result obtained on unspiked samples).

For an impurity test, discrimination can be established by spiking drug substance or drug product with appropriate levels of impurities and demonstrating the unbiased measurement of these impurities individually and/or from other components in the sample matrix.

Impurities or related substances are not available:

If impurity, related substances or degradation product materials are unavailable, specificity can be demonstrated by comparing the test results of samples containing typical impurities, related substances or degradation products with a second well-characterized procedure (*e.g.,* pharmacopeial procedure or other validated orthogonal analytical procedure**).**

4.2 Working Range

Depending on the sample preparation (*e.g.,* dilutions) and the analytical procedure selected, the reportable range will lead to a specific working range. Typically, a corresponding set of sample concentrations or purity levels is presented to the analytical instrument and the respective signal responses are evaluated.

4.2.1 Response

4.2.1.1 Linear Response

A linear relationship between analyte concentration and response should be evaluated across the working range of the analytical procedure to confirm the suitability of the procedure for the intended use. The response can be demonstrated directly on the drug substance (*e.g.,* by dilution of a standard stock solution) or separate weighings of synthetic mixtures of the drug product components, using the proposed procedure.

Initially, linearity can be evaluated with a plot of signals as a function of analyte concentration or content. Test results should be evaluated by appropriate statistical methods (*e.g.,* by calculation of a regression line by the method of least squares).

Data derived from the regression line may help to provide mathematical estimates of the linearity. A plot of the data, the correlation coefficient or coefficient of determination, y-intercept and slope of the regression line should be provided. An analysis of the deviation of the actual data points from the regression line is helpful for evaluating linearity (*e.g.,* for a linear response, the impact of any non-random pattern in the residuals plot from the regression analysis should be assessed).

For the establishment of linearity, a minimum of five concentrations appropriately distributed across the range is recommended; however, additional concentrations may be required for more complex models. Other approaches should be justified.

To obtain linearity, the measurements can be transformed, and a weighting factor applied to the regression analysis (i.e., in case of populations of data points with different variability (heteroscedasticity), including log or square root).

Other approaches should be justified.

4.2.1.2 Non-linear Response

Some analytical procedures may show non-linear responses. In these cases, a model or function which can describe the relationship between response of the analytical procedure and the concentration is necessary. The suitability of the model should be assessed by means of non-linear regression analysis (*e.g.,* coefficient of determination).

For example, immunoassays or cell-based assays may show an S-shaped response. S-shaped test curves occur when the range of concentrations is wide enough that responses are

constrained by upper and lower asymptotes. Common models used in this case are four-parameter or five-parameter logistical functions, though other acceptable models exist.

For these analytical procedures, the evaluation of linearity is separate from consideration of the shape of the concentration-response curve. Thus, linearity of the concentration-response relationship is not required. Instead, analytical procedure capability should be evaluated across a given working range to obtain values that are proportional to the true (known or theoretical) sample values.

4.2.1.3 Multivariate calibration

Algorithms used for construction of multivariate calibration models can be linear or non-linear, as long as the model is appropriate for establishing the relationship between the signal and the quality attribute of interest. The accuracy of a multivariate procedure is dependent on multiple factors, such as the distribution of calibration samples across the calibration range and the reference procedure error.

Linearity assessment, apart from comparison of reference and predicted results, should include information on how the analytical procedure error (residuals) changes across the calibration range. Graphical plots can be used to assess the residuals of the model prediction across the working range.

4.2.2 Validation of lower range limits

The lower range limits, *detection limit* (DL) and *quantitation limit* (QL), can be estimated using different approaches.

4.2.2.1 Based on signal-to-noise

This approach can only be applied to analytical procedures which exhibit baseline noise. *Determination* of the signal-to-noise ratio is performed by comparing measured signals from samples with known low concentrations of analyte with those of blank samples. Signals in an appropriate baseline region can be used instead of blank samples. The DL or QL are the minimum concentrations at which the analyte can be reliably detected or quantified, respectively. A signal-to-noise ratio of 3:1 is generally considered acceptable for estimating the detection limit. For quantitation limit, a ratio of at least 10:1 is considered acceptable.

For chromatographic procedures, the signal-to-noise ratio should be determined within a defined region and, if possible, situated equally around the place where the peak of interest would be found.

4.2.2.2 Based on the Standard Deviation of a Linear Response and a Slope

The detection limit (DL) can be expressed as:

$$DL = \frac{3.3\sigma}{S}$$

while the quantitation limit (QL) can be expressed as:

$$QL = \frac{10\sigma}{S}$$

where σ = the standard deviation of the response

S = the slope of the calibration curve

The slope S can be estimated from the regression line of the analyte. The estimate of σ can be carried out in a variety of ways, for example:

<u>Based on the Standard Deviation of the Blank</u>

Measurement of the magnitude of background response is performed by analysing an appropriate number of blank samples and calculating the standard deviation of the responses.

<u>Based on the Calibration Curve</u>

A specific calibration curve should be evaluated using samples containing an analyte in the range of the DL and QL. The residual standard deviation of a regression line (i.e., root mean square error/deviation) or the standard deviation of y-intercepts of the regression lines can be used as the standard deviation.

<u>Based on visual evaluation</u>

Visual evaluation can be used for both non-instrumental and instrumental procedures.

The limit is determined by the analysis of samples with known concentrations and by establishing the minimum level at which the analyte can be reliably resolved and detected or quantified.

4.2.2.3 Based on Accuracy and Precision at lower range limits

Instead of using estimated values as described in the previous approaches, the QL can be directly validated by accuracy and precision measurements.

4.2.2.4 Recommended Data

The DL and the approach used for its determination should be presented. If the DL is determined based on visual evaluation or based on signal to noise ratio, the presentation of the relevant data is considered an acceptable justification.

In cases where an estimated value for the DL is obtained by calculation or extrapolation, this estimate can subsequently be validated by the independent analysis of a suitable number of samples known to be near or prepared at the DL.

Also, the QL and the approach used for its determination should be presented.

If the QL was estimated, the limit should be subsequently validated by the analysis of a suitable number of samples known to be near or at the QL. In cases where the QL is well below (*e.g.,* approximately 10 times lower than) the reporting limit, this confirmatory validation can be omitted with justification.

For impurity tests, the quantitation limit for the analytical procedure should be equal to or below the reporting threshold.

4.3 Accuracy and Precision

Accuracy and precision can be evaluated independently, each with a predefined acceptance criterion. Combining these performance characteristics is an alternative approach for evaluation of analytical procedure suitability described in this chapter.

4.3.1 Accuracy

Accuracy should be established across the reportable range of an analytical procedure and is typically demonstrated through comparison of the measured results with an expected value. Accuracy should be demonstrated under regular test conditions of the analytical procedure (*e.g.,* in the presence of sample matrix and using described sample preparation steps).

Accuracy is typically verified through one of the studies described below. In certain cases (*e.g.,* small molecule drug substance assay), accuracy can be inferred once precision, response within the working range and specificity have been established.

4.3.1.1 Reference material comparison

The analytical procedure is applied to an analyte of known purity (*e.g.,* a reference material, a well characterized impurity or a related substance) and the measured *versus* theoretically expected result is evaluated.

4.3.1.2 Spiking Study

The analytical procedure is applied to a matrix of all components except the analyte where a known amount of the analyte of interest has been added. In cases where all the expected components are impossible to reproduce, known quantities of the analyte can be added to the test sample. The results from measurements on unspiked and spiked samples are evaluated.

4.3.1.3 Orthogonal Procedure comparison

The results of the proposed analytical procedure are compared with those of a second well-characterized procedure that ideally applies a different measurement principle (independent

procedure, see 1.2.). The accuracy of this second procedure should be reported. Orthogonal procedures can be used with quantitative impurity measurements to verify primary measurement values in cases where obtaining samples of all relevant components needed to mimic the matrix for spike recovery studies is not possible.

4.3.1.4 Recommended Data

Accuracy should be assessed using an appropriate number of determinations and concentration levels covering the reportable range (*e.g.,* 3 concentrations/3 replicates each of the full analytical procedure).

Accuracy should be reported as the mean percent recovery by the assay of a known added amount of analyte in the sample or as the difference between the mean and the accepted true value together with the confidence intervals.

An appropriate confidence interval (*e.g.,* 95%) for the mean percent recovery or the difference between the mean and accepted true value (as appropriate) should be compared to the acceptance criterion to evaluate analytical procedure bias. The appropriateness of the confidence interval should be justified.

For assay, the confidence intervals found should be compatible with the corresponding assay specification.

For impurity tests, the approach for the determination of individual or total impurities should be described (*e.g.,* weight/weight or area percent with respect to the major analyte).

For quantitative applications of multivariate analytical procedures, appropriate metrics, *e.g.,* root mean-squared error of prediction (RMSEP), should be used. If RMSEP is found to be comparable to acceptable root mean-squared error of calibration (RMSEC) then this indicates that the model is accurate enough when tested with an independent test set. Qualitative applications such as classification, misclassification rate or positive prediction rate can be used to characterize accuracy.

4.3.2 Precision

Validation of tests for assay and for quantitative determination of impurities or purity includes an investigation of precision.

Precision should be investigated using homogeneous, authentic samples or artificially prepared samples (e.g., matrix mixtures spiked with relevant amounts of the analyte in question). If a homogeneous sample is not available, then artificially prepared samples or a sample solution can be used.

4.3.2.1 Repeatability

Repeatability should be assessed using:

a) a minimum of 9 determinations covering the reportable range for the procedure (e.g., 3 concentrations/3 replicates each);

or

b) a minimum of 6 determinations at 100% of the test concentration.

4.3.2.2 Intermediate Precision

The extent to which *intermediate precision* should be established depends on the circumstances under which the procedure is intended to be used. The applicant should establish the effects of random events on the precision of the analytical procedure. Typical variations to be studied include different days, environmental conditions, analysts and equipment, as relevant. Ideally, the variations tested should be based on and justified by using analytical procedure understanding from development and risk assessment (*ICH Q14*). Studying these effects individually is not necessary. The use of design of experiments studies is encouraged.

4.3.2.3 Reproducibility

Reproducibility is assessed by means of an inter-laboratory trial. Investigation of reproducibility is usually not required for regulatory submission but should be considered in cases of standardization of an analytical procedure, for instance, for inclusion of procedures in pharmacopoeias.

4.3.2.4 Recommended Data

The standard deviation, relative standard deviation (coefficient of variation) and confidence interval should be reported for each type of precision investigated and be compatible with the specification limits.

Additionally, for multivariate analytical procedures, the routine metrics of RMSEP encompass accuracy and precision.

4.3.3 Combined approaches for accuracy and precision

An alternative to separate evaluation of accuracy and precision is to consider their total impact by assessing against a combined performance criterion. The approach should be reflective of the individual criteria that would have been established for accuracy and precision.

Data generated during development may help determine the best approach and refine appropriate performance criteria to which combined accuracy and precision are compared.

Combined accuracy and precision can be evaluated by use of a prediction interval (to assess the probability that the next reportable value falls within the acceptable range) or a tolerance interval (to assess the proportion of all future reportable values that will fall within the acceptable range). Other approaches may be acceptable if justified.

4.3.3.1 Recommended Data

If a combined performance criterion is chosen, results should be reported as combined value to provide appropriate overall knowledge of the suitability of the analytical procedure. If relevant, the individual results for accuracy and precision should be provided as supplemental information. The approach used should be described.

4.4 Robustness

The evaluation of the analytical procedure's suitability within the intended operational environment should be considered during the development phase and depends on the type of procedure under study. Robustness testing should show the reliability of an analytical procedure with respect to deliberate variations in parameters. The robustness evaluation can be submitted as part of development data for an analytical procedure on a case-by-case basis or should be made available upon request.

For further details, see ICH Q14.

5 GLOSSARY

ACCURACY

The accuracy of an analytical procedure expresses the closeness of agreement between the value which is accepted either as a conventional true value or as an accepted reference value and the value measured. (ICH Q2)

ANALYTICAL PROCEDURE

The analytical procedure refers to the way of performing the analysis. The analytical procedure description should include in detail the steps necessary to perform each analytical test. (ICH Q2)

ANALYTICAL PROCEDURE ATTRIBUTE

A technology specific property that should be within an appropriate limit, range or distribution to ensure the desired quality of the measured result. For example, attributes for chromatography measurements may include peak symmetry factor and resolution. (ICH Q14)

ANALYTICAL PROCEDURE CONTROL STRATEGY

A planned set of controls derived from current analytical procedure understanding that ensures the analytical procedure performance and the quality of the measured result. (ICH Q14)

ANALYTICAL PROCEDURE PARAMETER

Any factor (including reagent quality) or analytical procedure operational step that can be varied continuously (e.g., flow rate) or specified at controllable, unique levels. (ICH Q14)

ANALYTICAL PROCEDURE VALIDATION STRATEGY

An analytical procedure validation strategy describes how to select the analytical procedure performance characteristics for validation. In the strategy, data gathered during development studies (e.g., using *MODR* or *PAR*) and system suitability tests (SSTs) can be applied to validation and an experimental scheme for future movements of parameters within an MODR/PAR can be predefined. (ICH Q14)

ANALYTICAL TARGET PROFILE (ATP)

A prospective summary of the performance characteristics describing the intended purpose and the anticipated performance criteria of an analytical measurement. (ICH Q14)

CALIBRATION MODEL

A model based on analytical measurements of known samples that relates the input data to a value for the property of interest (i.e., the model output). (ICH Q2)

CONTROL STRATEGY

A planned set of controls, derived from current product and process understanding, that assures process performance and product quality. The controls can include parameters and attributes related to drug substance and drug product materials and components, facility and equipment operating conditions, in-process controls, finished product specifications, and the associated methods and frequency of monitoring and control. (ICH Q10)

CO-VALIDATION

Demonstration that the analytical procedure meets its predefined performance criteria when used at different laboratories for the same intended purpose. Co-validation can involve all (full revalidation) or a subset (partial revalidation) of performance characteristics potentially impacted by the change in laboratories. (ICH Q2)

CRITICAL QUALITY ATTRIBUTE (CQA)

A physical, chemical, biological or microbiological property or characteristic that should be within an appropriate limit, range, or distribution to ensure the desired product quality. (ICH Q8)

CROSS-VALIDATION

Demonstration that two or more analytical procedures meet the same predefined performance criteria and can therefore be used for the same intended purpose. (ICH Q2)

DETECTION LIMIT

The detection limit is the lowest amount of an analyte in a sample which can be detected but not necessarily quantitated as an exact value. (ICH Q2)

DETERMINATION

The reported value(s) from single or replicate measurements of a single sample preparation as per the validation protocol. (ICH Q2)

ESTABLISHED CONDITIONS (ECs)

ECs are legally binding information considered necessary to assure product quality. As a consequence, any change to ECs necessitates a submission to the regulatory authority. (ICH Q12)

INTERMEDIATE PRECISION

Intermediate precision expresses within-laboratories variations. Factors to be considered should include potential sources of variability, for example, different days, different environmental conditions, different analysts and different equipment. (ICH Q2)

KNOWLEDGE MANAGEMENT

A systematic approach to acquiring, analysing, storing and disseminating information related to products, manufacturing processes and components. (ICH Q10)

METHOD OPERABLE DESIGN REGION (MODR)

A combination of analytical procedure parameter ranges within which the analytical procedure performance criteria are fulfilled and the quality of the measured result is assured. (ICH Q14)

ONGOING MONITORING

The collection and evaluation of analytical procedure performance data to ensure the quality of measured results throughout the analytical procedure lifecycle. (ICH Q14)

PERFORMANCE CHARACTERISTIC

A technology independent description of a characteristic to ensure the quality of the measured result. Typically, accuracy, precision, specificity/selectivity and range may be considered. The term was previously called VALIDATION CHARACTERISTIC. (ICH Q2)

PERFORMANCE CRITERION

An acceptance criterion describing a numerical range, limit or desired state to ensure the quality of the measured result. (ICH Q14)

PLATFORM ANALYTICAL PROCEDURE

A platform analytical procedure can be defined as a multi-product method suitable to test quality attributes of different products without significant change to its operational conditions, system suitability and reporting structure. This type of method would apply to molecules that are sufficiently alike with respect to the attributes that the platform method is intended to measure. (ICH Q2)

PRECISION

The precision of an analytical procedure expresses the closeness of agreement (degree of scatter) between a series of measurements obtained from multiple samplings of the same homogeneous sample under the prescribed conditions. Precision can be considered at three levels: repeatability, intermediate precision and reproducibility.

The precision of an analytical procedure is usually expressed as the variance, standard deviation or coefficient of variation of a series of measurements. (ICH Q2)

PROVEN ACCEPTABLE RANGE FOR ANALYTICAL PROCEDURES (PAR)

A characterised range of an analytical procedure parameter for which operation within this range, while keeping other parameters constant, will result in an analytical measurement meeting relevant performance criteria. (ICH Q14)

QUALITY RISK MANAGEMENT

A systematic process for the assessment, control, communication and review of risks to the quality of the drug (medicinal) product across the product lifecycle. (ICH Q9)

QUANTITATION LIMIT

The quantitation limit is the lowest amount of analyte in a sample which can be quantitatively determined with suitable precision and accuracy. The quantitation limit for an analytical procedure should not be more than the reporting threshold. The quantitation limit is a parameter used for quantitative assays for low levels of compounds in sample matrices, and, particularly, is used for the determination of impurities and/or degradation products. (ICH Q2)

RANGE

The range of an analytical procedure is the interval between the lowest and the highest reportable results in which the analytical procedure has a suitable level of precision, accuracy and response. (ICH Q2)

REPORTABLE RANGE

The reportable range of an analytical procedure includes all values from the lowest to the highest reportable result for which there is a suitable level of precision and accuracy. Typically, the reportable range is given in the same unit as the specification. (ICH Q2)

WORKING RANGE

The working range of an analytical procedure is the lowest and the highest concentration that the analytical procedure provides meaningful results. Working ranges may be different before sample preparation (sample working range) and when presented to the analytical instrument (instrument working range). (ICH Q2)

REAL TIME RELEASE TESTING (RTRT)

The ability to evaluate and ensure the quality of the in-process and/or final product based on process data, which typically include a valid combination of measured material attributes and process controls. (ICH Q8)

REPEATABILITY

Repeatability expresses the precision under the same operating conditions over a short interval of time. Repeatability is also termed intra-assay precision. (ICH Q2)

REPORTABLE RESULT

The result as generated by the analytical procedure after calculation or processing and applying the described sample replication. (ICH Q2)

REPRODUCIBILITY

Reproducibility expresses the precision between laboratories (e.g., inter-laboratory studies, usually applied to standardization of methodology). (ICH Q2)

RESPONSE

The response of an analytical procedure is its ability (within a given range) to obtain a signal which is effectively related to the concentration (amount) of analyte in the sample by some known mathematical function. (ICH Q2)

REVALIDATION

Demonstration that an analytical procedure is still fit for its intended purpose after a change to the product, process or the analytical procedure itself. Revalidation can involve all (full revalidation) or a subset (partial revalidation) of performance characteristics. (ICH Q2)

ROBUSTNESS

The robustness of an analytical procedure is a measure of its capacity to meet the expected performance requirements during normal use. Robustness is tested by deliberate variations of analytical procedure parameters. (ICH Q14)

SAMPLE SUITABILITY ASSESSMENT

A sample or sample preparation is considered suitable if the measurement response on the sample satisfies pre-defined acceptance criteria for the analytical procedure attributes that have been developed for the validated analytical procedure. Sample suitability is a pre-requisite for the validity of the result along with a satisfactory outcome of the system suitability test. Sample suitability generally consists of the assessment of the similarity of the response between a standard and the test sample and may include a requirement of no interfering signals arising from the sample matrix. (ICH Q14)

SPECIFICITY/SELECTIVTY

Specificity and selectivity are both terms to describe the extent to which other substances interfere with the determination of a substance according to a given analytical procedure. Such other substances might include impurities, degradation products, related substances, matrix or other components present in the operating environment. Specificity is typically used to describe the ultimate state, measuring unequivocally a desired analyte. Selectivity is a relative term to describe to which extent particular analytes in mixtures or matrices can be measured without interferences from other components of similar behaviour. (ICH Q2)

SYSTEM SUITABILITY TEST (SST)

These tests are developed and used to verify that the measurement system and the analytical operations associated with the analytical procedure are adequate for the intended analysis and increase the detectability of potential failures (ICH Q14)

TOTAL ANALYTICAL ERROR

Total analytical error (TAE) represents the overall error in a test result that is attributed to imprecision and inaccuracy. TAE is the combination of both, systematic error of the procedure and random measurement error. (ICH Q14)

VALIDATION STUDY

An evaluation of prior knowledge, data or deliberate experiments to determine the suitability of an analytical procedure for its intended purpose. (ICH Q2)

VALIDATION TEST

Validation tests are deliberate experiments designed to authenticate the suitability of an analytical procedure for its intended purpose. (ICH Q2)

MULTIVARIATE GLOSSARY

CALIBRATION DATA SET

A set of data with matched known characteristics and measured analytical results, that spans the desired operational range. (ICH Q2)

DATA TRANSFORMATION

Mathematical operation on model input data to assume better correlation with the output data and simplify the model structure. (ICH Q14)

INDEPENDENT SAMPLE

Independent samples are samples not included in the calibration set of a multivariate model. Independent samples can come from the same batch from which calibration samples are selected. (ICH Q2)

INTERNAL TESTING

Internal testing is a process of checking if unique samples processed by the model yield the correct predictions (qualitative or quantitative).

Internal testing serves as means to establish the optimal number of latent variables, estimate the standard error and detect potential outliers. Internal testing is preferably done by using samples not included in the calibration set. Alternatively, internal testing can be done using a subset of calibration samples, while temporarily excluding them from the model calculation. (ICH Q2)

INTERNAL TEST SET

A set of data obtained from samples that have physical and chemical characteristics that span a range of variabilities similar to the samples used to construct the calibration set. (ICH Q14)

LATENT VARIABLES

Mathematically derived variables that are directly related to measured variables and are used in further processing. (ICH Q2)

MODEL VALIDATION

The process of determining the suitability of a model by challenging it with independent test data and comparing the results against prespecified criteria. For quantitative models, validation involves confirming the calibration model's performance with an independent dataset. For identification libraries, validation involves analysing samples (a.k.a., challenge samples) not represented in the library to demonstrate the discriminative ability of the library model. (ICH Q2)

MODEL MAINTENANCE

Safeguards over the lifecycle of a multivariate model to ensure continued model performance, often including outlier diagnostics and resulting actions for model redevelopment or change in the maintenance plans. (ICH Q14)

MULTIVARIATE ANALYTICAL PROCEDURE

An analytical procedure where a result is determined through a multivariate calibration model utilizing more than one input variable. (ICH Q2)

OUTLIER DIAGNOSTIC

Tests that can identify unusual or atypical data in a multivariate analytical procedure. (ICH Q14)

REFERENCE PROCEDURE

A separate analytical procedure used to obtain the reference values of the calibration and validation samples for a multivariate analytical procedure. (ICH Q2)

REFERENCE SAMPLE

A sample representative of the test sample with a known value for the property of interest, used for calibration. (ICH Q14)

VALIDATION SET

A set of data used to give an independent assessment of the performance of the calibration model, ideally over a similar operating range. (ICH Q14)

6 References

ICH Q14 Analytical Procedure Development

7 ANNEX 1 SELECTION OF VALIDATION TESTS

Figure 2: Selection of validation tests based on the objective of the analytical procedure

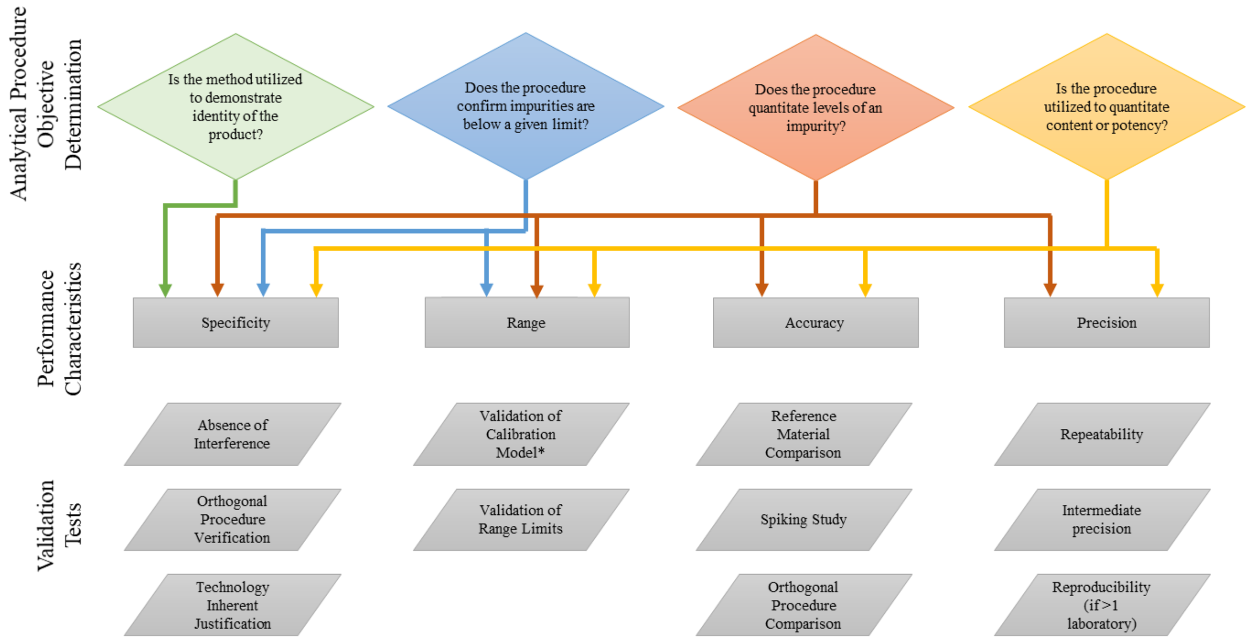

* May not be needed for limit test

8 ANNEX 2 ILLUSTRATIVE EXAMPLES FOR ANALYTICAL TECHNIQUES

Table 3: Examples for Quantitative separation techniques

**Technique**	**Separation techniques (HPLC, GC, CE) for impurities or assay**	**Separation techniques with Relative Area Quantitation, e.g., product-related substances such as charge variants**
**Performance characteristic**	**Validation study methodology**	
Specificity / Selectivity	<u>Absence of relevant interference:</u><br>With DS, DP, buffer, or appropriate matrix, and between individual peaks of interest<br><br>Spiking with known impurities / excipients<br><br>or<br><br>By comparison of impurity profiles by a secondary method<br><br>Demonstration of stability-indicating properties through appropriate forced degradation samples, if necessary.	<u>Absence of relevant interference:</u><br>With DS, DP, buffer, or appropriate matrix, and between individual peaks of interest<br><br>Demonstration of stability-indicating properties through appropriate forced degradation samples if necessary.
Precision	<u>Repeatability:</u><br>Replicate measurements with 3 times 3 levels across the reportable range or 6 times at 100% level, considering peak(s) of interest<br><br><u>Intermediate precision</u>:<br>Across e.g., days, environmental conditions, analysts, equipment	
Accuracy	For Assay:<br>Comparison with suitably characterized material (e.g., standard)<br><br>or<br><br>Comparison with well-defined secondary procedure<br><br>For impurities or related substances:<br><br>Spiking/Recovery experiments with impurities<br><br>Comparison of impurity profiles with well-defined secondary procedure	Comparison with well-defined secondary procedure and/or well-defined material (e.g., reference materials)<br><br>and/or, accuracy can be inferred once precision, linearity and specificity have been established.<br><br>and/or if needed,<br>Spike/Recovery experiments with forced degradation samples and/or well-defined material

**Technique**	**Separation techniques (HPLC, GC, CE) for impurities or assay**	**Separation techniques with Relative Area Quantitation, e.g., product-related substances such as charge variants**
**Performance characteristic**	**Validation study methodology**	
Reportable Range	Validation of calibration model across the range:<br><br>Linearity: Dilution of the analytes of interest over the expected procedure range, at least 5 points<br><br>Validation of lower range limits (for purity only): QL, DL through one selected methodology, *e.g.,* signal-to-noise determination	Validation of calibration model across the range:<br>Linearity: between measured (observed) relative result *versus* theoretically expected relative result across specification range(s); e.g., by spiking or degrading material<br><br>Validation of lower range limits: QL (and DL) through selected methodology from Section 5.2 (e.g., signal-to-noise determination).
Robustness (performed as part of analytical procedure development as per Q14)	Deliberate variation of parameters and stability of test conditions, e.g.,<br>Deliberate variations of test and sample preparation conditions, for example mobile phase, separation buffer, carrier gas composition and pH, columns, capillaries, temperature, extraction time,<br>Stability of SST, test and reference solutions	

Table 4: Example for Elemental Impurities by ICP-OES or ICP-MS as purity test

**Technique**	**Elemental Impurities by ICP-OES or ICP-MS as purity test**
**Performance characteristic**	**Validation study methodology**
Specificity / Selectivity	Spiking experiments of elements into matrix and demonstration of sufficient non-interference and verification of accuracy/recovery:<br><br>with the presence of components (*e.g.,* carrier gas, impurities, matrix)<br><br>or justification through technology/prior knowledge (*e.g.,* specificity of technology for certain isotopes)
Precision	Repeatability:<br>Replicate measurements with 3 times 3 levels across the reportable range or 6 times at 100% level, considering signals of interest<br><br>Intermediate precision:<br>*e.g.,* across days, environmental conditions, analysts, equipment
Accuracy	Spiking/Recovery experiments with impurities<br><br>or<br><br>Comparison of impurity profiles with well-defined secondary procedure
Reportable Range	Validation of working range:<br><br>Linearity: Dilution of the analytes of interest over the expected procedure range, at least 5 points, can be combined with multi-level accuracy experiment<br><br>Validation of lower range (for impurities only): QL, DL through one selected methodology
Robustness (performed as part of analytical procedure development as per Q14)	Deliberate variation of parameters and stability of test conditions:<br>Sample digestion technique and preparation, nebulizer and sheath flow settings, plasma settings

Table 5: Example for Dissolution with HPLC as product performance test for an immediate release dosage form

**Technique**	**Dissolution with HPLC as product performance test for an immediate release dosage form**	
**Performance characteristic**	**Demonstration of performance of dissolution step**<br>*Typically demonstrated with development data*	**Validation testing methodology**<br>*Typically demonstrated with final procedure*
Specificity/Selectivity	Discriminatory power:<br>Demonstration of sufficiently different dissolution of acceptable versus non-acceptable batches	Absence of interference<br>Demonstration of non-interference with excipients and dissolution media likely to impact the quantification of the main analyte
Precision	Precision and intermediate precision:<br>Repeated dissolution experiments of a well-characterized product batch representative for the manufacturing process.<br>*Note: The study will allow a combined assessment of product and analytical variations*	Precision and Intermediate Precision:<br>Demonstration with a homogeneous sample from one dissolved tablet, *e.g.,* several samples drawn from the same vessel, after analyte in sample has been fully solubilized
Accuracy	(Not applicable for dissolution step)	Spiking Study:<br>Add known amounts of the drug reference substance to the dissolution vessel containing excipient mixture in dissolution media and calculate recovery within defined working range.
Reportable Range	(Not applicable for dissolution step)	Validation of calibration model across the range<br>Linearity:<br>Demonstrate linearity from sample concentrations (as presented to quantitative measurement) in the range of Q-45% up to 120% of the content stated on the label, for immediate-release solid dosage forms.<br><br>*If lower concentration ranges are close to QL*:<br>Validation of lower range limits, see separation techniques
Robustness (done as part of analytical procedure development as per Q14)	Justification of the selection of the dissolution procedure parameters, e.g., medium composition buffer or surfactant concentration, use of sinkers, pH, deaeration, volume, agitation rate, sampling time	Deliberate variation of parameters of the quantitative procedure, see separation technique

Table 6: Example for Quantitative ^{1}H-NMR for the Assay of an API

**Technique**	**Quantitative ^{1}H-NMR (internal standard method) for the Assay of an API**
**Performance characteristic**	**Validation testing methodology**
Specificity / Selectivity	Absence of interference:<br>Identify a signal which is representative for the analyte and does not show interference with potential baseline artefacts, residual water or solvent signals, related structure impurities or other impurities, internal standards, non-target major component or potential isomers/forms.
Precision	Repeatability:<br>Replicate measurements of at least 6 independent preparations at 100% level<br><br>Intermediate Precision:<br>Not necessary to be conducted on target analyte (justified by technology principle, as typically verified through instrument calibration with a standard sample)
Accuracy	Reference material comparison<br>verify with sample of known purity
Reportable Range	Technology inherent justification:<br>Not necessary as the integral areas are directly proportional to the amount (mole) of reference standard and analyte.
Robustness (performed as part of analytical procedure development as per Q14)	Deliberate variation of parameters, e.g.,<br>Temperature,<br>Concentration,<br>Field (shim),<br>Tuning and Matching of the NMR probe<br><br>Stability over the use period of the test, *e.g.,* solution stability

Table 7: Example for Biological Assays

**Technique**	**Binding assay (e.g., ELISA, SPR) or Cell-based assay for determination of potency relative to a reference**
**Performance characteristic**	**Validation testing methodology**
Specificity / Selectivity	Absence of interference:<br>Dose-response curve fulfils the response criteria demonstrating the similarity of the analyte and reference material, as well as non-interfering signal from the matrix, no dose-response from the cell line alone<br><br>Demonstration of stability-indicating properties through appropriate forced degradation samples if necessary.
Precision	Repeatability:<br>Repeated sample analysis on a single day or within a short interval of time covering the response range of the method (NLT 3 replicates at NLT 5 levels)<br><br>Intermediate Precision: Different analysts, Multiple independent preparations over multiple days at multiple potency levels through the method's range, inclusive of normal laboratory variation
Accuracy	Reference material comparison:<br>Assess recovery versus theoretical activity for multiple (NLT 3) independent preparations at multiple (NLT 5) levels through the method's range
Reportable Range	Validation of lower and higher range limits:<br>The lowest to highest relative potency levels that meet accuracy, precision, and response criteria, determined as NLT 5 mean potency levels
Robustness (performed as part of analytical procedure development as per Q14)	Deliberate variation of parameters, e.g.,<br>Reagent lots (*e.g.,* Capture/detection antibody, coating proteins, controls)<br>Cell density, effector/target cell ratio, cell generation number<br>Plate type<br>Buffer components<br>Incubation times<br>Incubation conditions<br>Instruments<br>Reaction times<br>Impact of sample degradation

Table 8: Example for quantitative PCR

**Technique**	**Quantitative PCR (quantitative analysis of impurities in drug substances or products)**
**Performance characteristic**	**Validation testing methodology**
Specificity / Selectivity	<u>Orthogonal Procedure Comparison:</u><br>Test reaction specificity by electrophoresis gel, melting profile or DNA sequencing<br><u>Absence of interference:</u><br>- Positive template, no-reverse transcription control for RT-qPCR and no template control<br>- Test probe target specificity against gene bank (nucleotide blast).<br>- Evaluate the slope of standard curve for efficiency
Precision	<u>Repeatability:</u><br>With n=6 replicates and calculation of inter-run variance: slopes, coefficient of variation (CV) and y-intercepts are compared using the criteria of 2 standard deviations for the set of curves, if justified.<br><br><u>Intermediate precision</u><br>Comparison of measurements using the same procedure performed by another analyst on a different day.
Accuracy	<u>Spiking Study:</u><br>Test (*e.g.,* n=6) replicates at 3 to 5 template spike levels from the standard curve concentrations.<br>Efficiency/consistency of RNA/DNA extraction method should be accounted for
Reportable Range	<u>Linearity:</u><br>Linear working range should cover at least 5 to 6 log to the base 10 concentration values. Correlation coefficients or standard deviations should be calculated through the entire linear dynamic range.<br><br><u>Validation of lower working range limits based on the calibration Curve:</u><br><br>DL defined by template spiking in samples or from standard curves<br>DL is lowest point meeting the selected curve parameters, *e.g.,* coefficient of determination (R^2), efficiency, 1st order polynomial fit and a standard deviation of the kurtosis distribution<br><br>QL demonstrated through demonstrating sufficient recovery and acceptable coefficient of variations from the accuracy experiment
Robustness (performed as part of analytical procedure development as per Q14)	<u>Deliberate variation of parameters, e.g.,</u><br>Equipment<br>Master mix composition (concentrations of salts, dNTPs, adjuvants)<br>Master mix lots<br>Reaction volume<br>Probe and primer concentrations<br>Thermal cycling parameters

Table 9: Example for particle size measurement

**Technique**	**Particle size measurement (Dynamic light scattering; Laser diffraction measurement) as property test**
**Performance characteristic**	**Validation testing methodology**
Specificity / Selectivity	Absence of interference:<br><br>If needed, evaluate blank and sample to determine the appropriateness of the equipment settings and sample preparation
Precision	Repeatability:<br>test at least n=6 replicates at established analytical procedure parameters at target range.<br><br>Intermediate precision: analysis performed on different days, environmental conditions, analysts, equipment setup
Accuracy	Technology inherent justification:<br>confirmed by an appropriate instrument qualification<br><br>Or<br><br>Alternative option: Orthogonal Procedure comparison:<br>If needed, qualitative comparison using a different technique, like optical microscopy, to confirm results
Reportable Range	Technology specific justification, *e.g.,* particle size range covered
Robustness (performed as part of analytical procedure development as per Q14)	Deliberate variation of parameters, e.g.,<br>Evaluation of expected size ranges of the intended use of the analytical procedure.<br>Dispersion stability for liquid dispersions (stability over potential analysis time, stir rate, dispersion energy equilibration or stir time before measurement)<br>Dispersion Stability for dry dispersions (sample amount, measurement time, air pressure and feed rate)<br>Obscuration range (establish optimum percentage of laser obscuration);<br>Ultrasound time, if applicable<br>Ultrasound percentage, if applicable.

Table 10: NIR

**Technique**	**NIR method validation example for core tablet assay**
**Performance characteristic**	**Validation testing methodology**
Specificity / Selectivity	<u>Absence of interference</u>:<br><br>Comparison of API spectrum and the loadings plots of the model<br><br>Rejection of outliers (e.g., excipient, analogues) not covered by the multivariate procedure
Precision	<u>Repeatability:</u><br>Repeated analysis with removal of sample from the holder between measurements.
Accuracy	<u>Comparison with well-defined secondary procedure:</u><br>Demonstration across the range through comparison of the predicted and reference values using an appropriate number of determinations and concentration levels (e.g., 5 concentrations, 3 replicates). Accuracy is typically reported as the standard error of prediction (SEP or RMSEP).
Reportable Range	<u>Linearity:</u><br>Demonstration of the linear relationship between predicted and reference values.<br><br><u>Error (accuracy) across the range:</u><br>Information on how the method error (accuracy) changes across the calibration range, e.g., by plotting the residuals of the model prediction vs. the actual data.
Robustness (performed as part of analytical procedure development as per Q14)	<u>Robustness</u><br>Chemical and physical factors that can impact NIR spectrum and model prediction should be represented in data sets. Examples include various sources of API and excipients, water content, tablet hardness, and orientation in the holder.<br><br>*Note: NIR measurements are sensitive to changes in tablets composition and properties outside variation present in the calibration set.*

686 **Table 11: Example for Quantitative LC/MS**

**Technique**	**Quantitative LC/MS (quantitative analysis of impurities (e.g., genotoxic impurities) in drug substances or products)**
**Performance characteristic**	**Validation testing methodology**
Specificity / Selectivity	Technology inherent justification:<br>Inferred through use of specific and selective MS detection *(*e.g*.,* MRM transition with specified quantitative to qualitative ion ratio, accurate m/z value) in combination with retention time, consider potential for isotopes<br><br>Absence of interference:<br>from other components in sample matrix.<br><br>Orthogonal procedure comparison:<br>By comparison of impurity profiles determined by an alternative validated method
Precision	Repeatability<br>Measurement of at least three replicates at each of at least three spiking levels<br><br>Intermediate precision<br>Comparison of measurements of the same samples performed in the same laboratory but under varying conditions (e.g., different LC/MS systems, different analysts, different days). Comparison of measurements of the same samples made in different laboratories
Accuracy	Spiking Study<br>Acceptable recovery of spiked impurity standards in sample matrix at multiple spiking levels<br><br>Or:<br><br>Comparison with well-defined secondary procedure:<br><br>Comparison of the measurement results to the 'true' values obtained from alternative validated procedures
Reportable Range	Validation of calibration model across the range:<br><br>Linearity: Experimental demonstration of the linear relationship between analyte concentrations and peak responses (or the ratio of peak response if an internal standard was used) with reference materials at 5 or more concentration levels<br><br>Validation of lower range limits:<br>DL:<br>Use the measured signal to noise of the spiking level with coefficient of variation (CV) or calculated relative standard deviation (RSD or %RSD)

**Technique**	**Quantitative LC/MS (quantitative analysis of impurities (e.g., genotoxic impurities) in drug substances or products)**
**Performance characteristic**	**Validation testing methodology**
	of responses (with 6 or more repeated injections) less than pre-defined acceptable value.<br><br>QL:<br>The lowest spiking level with acceptable accuracy and precision.<br><br>The analytical procedure range extends from and inclusive of the LOQ to the highest spiking level with acceptable accuracy, precision, and linearity
Robustness (performed as part of analytical procedure development as per Q14)	Deliberate variation of parameters and stability of test conditions:<br>The following factors should be considered during assessment of analytical procedure performance: LC flow rate, LC injection volume, MS drying/ desolvation temperature, MS gas flow, mass accuracy and MS collision energy.
