# Supplementary material for: Stability-Indicating RP-HPLC Determination of Acetylsalicylic Acid, Dextromethorphan Hydrobromide, Phenylephrine Bitartrate, and Chlorpheniramine Maleate in an Effervescent Tablet Formulation
Source: J AOAC Int. 2026 Aug 7;109(6):887–96. doi: 10.1093/jaoacint/qsag070 (PMC13732704; doi:10.1093/jaoacint/qsag070)
Supplement: Supplementary file 1 [file qsag070_supplementary_data.zip › qsag070_Supplementary_Data/aoac-25-0299-File012.pdf]

14 December 2023
EMA/CHMP/ICH/195040/2022
Committee for Medicinal Products for Human Use

ICH Q14 Guideline on analytical procedure development

Step 5

Transmission to CHMP	8 March 2022
Adoption by CHMP	24 March 2022
Release for public consultation	31 March 2022
Deadline for comments	31 July 2022
Final adoption by CHMP	14 December 2023
Date for coming into effect*	14 June 2024*

* For ICH Q12 concepts within ICH Q14, refer to the Note for implementation of ICH Q12 (EMA/CHMP/ICH/78332/2020) for further details.

Official address Domenico Scarlattilaan 6 • 1083 HS Amsterdam • The Netherlands
Address for visits and deliveries Refer to www.ema.europa.eu/how-to-find-us
Send us a question Go to www.ema.europa.eu/contact **Telephone** +31 (0)88 781 6000
An agency of the European Union

© European Medicines Agency, 2026. Reproduction is authorised provided the source is acknowledged.

Document History

Code	History	Date
Q14	Endorsement by the Members of the ICH Assembly under Step 2 and release for public consultation	24 March 2022
Q14	Adoption by the Regulatory Members of the ICH Assembly under Step 4	1 November 2023
Q14	Error Corrections: Alignment of glossary terms (Intermediate precision, Performance Characteristic, Range, Reportable Range and Working Range) with ICH Q2(R2), Minor Format corrections to Table 1 and Table 6 and Figure 3 alignment to Table 6.	4 April 2025

ICH Q14 Guideline on analytical procedure development

Table of contents

1. Introduction ... 4

1.1 Objective ... 4

1.2 Scope ... 4

2. General considerations for analytical procedures ... 4

2.1 Minimal versus enhanced approaches to analytical procedure development ... 5

2.2 The analytical procedure lifecycle ... 5

3. Analytical target profile ... 6

4. Knowledge and risk management in analytical procedure development and continual improvement ... 7

4.1 Knowledge management ... 7

4.2 Risk management ... 7

5. Evaluation of robustness and parameter ranges of analytical procedures ... 8

5.1 Robustness ... 8

5.2 Analytical procedure parameter ranges ... 8

6. Analytical procedure control strategy ... 9

6.1 Established conditions for analytical procedures ... 10

7. Lifecycle management and post-approval changes of analytical procedures ... 10

8. Development of multivariate analytical procedures: additional considerations ... 14

9. Development of analytical procedures for real time release testing: additional considerations ... 17

10. Submission of analytical procedure related information ... 18

10.1 General regulatory considerations and documentation ... 18

10.2 Documentation for the enhanced approach ... 18

10.3 Documentation for multivariate analytical procedures ... 18

11. Glossary ... 19

12. References ... 25

13. Annex ... 26

13.1 Annex A: Examples of application of ICH Q14 principles ... 26

13.1.1 Measurement of stereoisomers as specific process related impurities in a small molecule drug substance (DS) ... 28

13.1.2 Measurement of potency for an anti-TNF-alpha monoclonal antibody ... 34

13.2. Annex B: Example of multivariate model lifecycle components ... 41

1. Introduction

1.1 Objective

This guideline describes science and risk-based approaches for developing and maintaining analytical procedures suitable for the evaluation of the quality of drug substances and drug products. The systematic approach suggested in ICH Q8 Pharmaceutical Development together with principles of ICH Q9 Quality Risk Management can also be applied to the development and lifecycle management of analytical procedures. When developing an analytical procedure, a minimal (also known as traditional) approach or elements of an enhanced approach can be applied. Furthermore, the guideline describes additional considerations for the development of multivariate analytical procedures and for real time release testing (RTRT).

This guideline complements ICH Q2 Validation of Analytical Procedures.

Using the tools described in ICH Q12 Technical and Regulatory Considerations for Pharmaceutical Product Lifecycle Management, the guideline describes principles to support change management of analytical procedures based on risk management, comprehensive understanding of the analytical procedure and adherence to predefined criteria for performance characteristics. Knowledge gained from application of an enhanced approach to analytical procedure development can provide better assurance of the performance of the procedure, can serve as a basis for the analytical procedure control strategy and can provide an opportunity for more efficient regulatory approaches to related post approval changes.

The guideline also describes submission of analytical procedure development and related lifecycle information in the Common Technical Document (CTD) format (ICH M4Q, The Common Technical Document for the Registration of Pharmaceuticals for Human Use). Information related to analytical procedure development and knowledge may be submitted to regulatory authorities to provide additional evidence that the analytical procedure is fit for the intended purpose. While the minimal approach remains a valid approach, an applicant can decide to submit additional development data and knowledge which may facilitate regulatory communication for post approval change management.

1.2 Scope

This guideline applies to analytical procedures used for release and stability testing of commercial drug substances and products, hereafter referred to as ‘products’. The guideline can also be applied to other analytical procedures used as part of the control strategy (ICH Q10 Pharmaceutical Quality System) following a risk-based approach. The scientific principles described in this guideline can be applied in a phase-appropriate manner to analytical procedures used during clinical development.

2. General considerations for analytical procedures

The goal of development is to obtain an analytical procedure fit for the intended purpose: to measure an attribute or attributes of the material with the needed specificity/selectivity, accuracy, precision over the reportable range. Details of the performance characteristics considered for analytical procedure validation are described in ICH Q2.

In this section the minimal and enhanced approaches to analytical procedure development are described. While the minimal approach remains an acceptable approach to development of a robust analytical procedure that is fit for the intended purpose, some or all elements of the enhanced approach might be used to support development and lifecycle management of analytical procedures.

In certain cases, an analytical procedure can be applied to multiple products with little or no modification of measurement conditions. For a new application of such platform analytical procedures, the subsequent development can be abbreviated, and certain validation tests can be omitted based on a science- and risk-based justification.

Data gained during the development studies (e.g., robustness data from a design of experiments (DoE) study) could be used as part of the validation data for the related analytical procedure performance characteristics and studies do not necessarily need to be repeated.

2.1 Minimal versus enhanced approaches to analytical procedure development

Analytical procedure development should include the following elements as a minimum:

- Identifying the attributes of the product which need to be tested;
- Selecting an appropriate technology and related instruments or suitable apparatus;
- Conducting studies to evaluate analytical procedure performance characteristics such as specificity, accuracy and precision over the reportable range (including the calibration model, lower and/or higher range limits) and robustness;
- Documenting the analytical procedure including the analytical procedure control strategy.

The enhanced approach offers a systematic way of developing and refining knowledge of an analytical procedure and demonstrating procedure understanding. Product and process understanding informs the quality attributes to be tested. The anticipated performance criteria for relevant performance characteristics should be documented in an analytical target profile (ATP). In addition to the elements of the minimal approach, an enhanced approach may include the following elements as appropriate:

- Conducting risk assessment and evaluating prior knowledge to identify the analytical procedure parameters that can impact performance of the procedure;
- Conducting uni- or multi-variate experiments and/or modelling to explore ranges and interactions between identified analytical procedure parameters;
- Defining an analytical procedure control strategy including set-points and/or ranges for relevant analytical procedure parameters. These could include proven acceptable ranges for analytical procedures (PARs) and/or method operable design regions (MODRs).

Applying elements of the enhanced approach to development can lead to a better understanding of the impact of analytical procedure parameters on the analytical procedure performance and more flexibility for lifecycle management, such as wider operating ranges and a more appropriate set of established conditions (ECs) with associated reporting categories.

2.2 The analytical procedure lifecycle

Figure 1 depicts elements of the analytical procedure lifecycle as related to the product lifecycle. Analytical procedure development and change management approaches are described in this guideline whereas analytical procedure validation is described in ICH Q2. Depending on the intended purpose of the analytical procedure and the development approach taken, the order and extent of each element could vary, and several elements could occur simultaneously.

Figure 1: The analytical procedure lifecycle

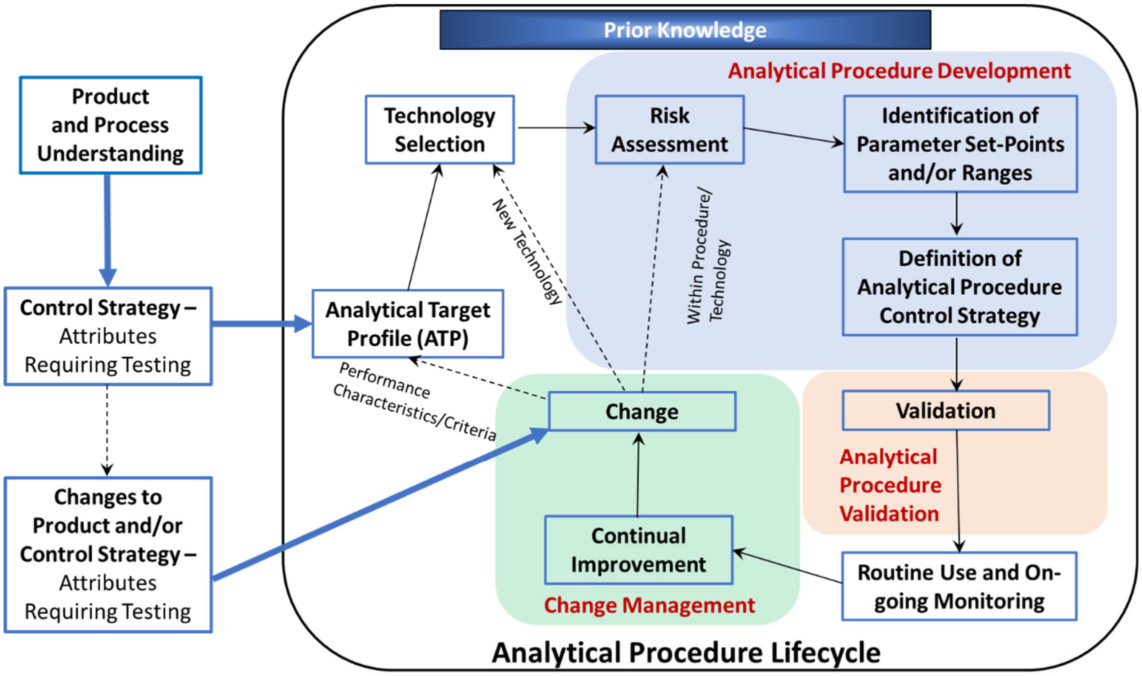

3. Analytical target profile

Product and process understanding (ICH Q8 and ICH Q11 Development and Manufacture of Drug Substances) leads to the identification of critical quality attributes (CQAs) requiring analytical measurement for control which may be included in the quality target product profile (QTPP). Measurement needs can be captured in an ATP which forms the basis for development of the analytical procedure. An ATP consists of a description of the intended purpose of the analytical procedure, appropriate details on the product attributes to be measured and relevant performance characteristics with associated performance criteria. The ATP includes measurement requirements for one or more quality attributes. The ATP drives the choice of analytical technology. Multiple available analytical techniques may meet the performance criteria. Consideration of the operating environment (e.g., at-line, in-line or off-line) should be included in the technology selection. Once a technology has been selected, the ATP serves as a foundation to derive the analytical procedure attributes and performance criteria for analytical procedure validation (ICH Q2). Formal documentation and submission of an ATP is optional but can facilitate regulatory communication irrespective of the chosen development approach.

The ATP also facilitates ongoing monitoring and continual improvement of the analytical procedure. The ATP is maintained over the lifecycle and can also be used as a basis for lifecycle management to ensure that the existing, revised or new analytical procedure remains fit for the intended purpose.

Illustrative examples of ATPs are provided in Annex A.

4. Knowledge and risk management in analytical procedure development and continual improvement

4.1 Knowledge management

As with product and manufacturing process development, knowledge management (ICH Q10) plays a critical role in analytical procedure development and during the lifecycle of the analytical procedure.

Prior knowledge is explicitly or implicitly used for informing decisions during analytical procedure development and lifecycle management. Prior knowledge can be internal knowledge from a company's proprietary development and analytical experience, external knowledge such as reference to scientific and technical publications or established scientific principles.

Prior product knowledge plays an important role in identifying suitable analytical techniques. Knowledge of best practices, state-of-the-art technologies and regulatory expectations contribute to the selection of the most suitable technology for a given purpose. Existing platform analytical procedures (e.g., protein content measurement by UV spectroscopy) can be leveraged to evaluate the attributes of a specific product without conducting additional procedure development.

As additional information is obtained, knowledge related to analytical procedures should be actively managed throughout the product lifecycle.

4.2 Risk management

The use of quality risk management (QRM) is encouraged to aid in the development of a robust analytical procedure to reduce risk of poor performance and reporting incorrect results. Risk assessment is typically performed early in analytical procedure development and is updated as more information becomes available. Risk assessment can be formal or informal and can be supported by prior knowledge.

Risk assessment tools as described in ICH Q9 Annex 1 can be used to:

- identify analytical procedure parameters (factors and operational steps) with potential impact on its performance, *e.g.,* Annex A Figure 2 (Ishikawa diagram);
- assess the potential impact of analytical procedure parameters on the analytical procedure performance;
- identify and prioritise analytical procedure parameters to be investigated experimentally;
- inform the need and the extent of ongoing monitoring as part of risk review.

Risk communication should be used to support continual improvement of the analytical procedure performance throughout its lifecycle. The outcome of quality risk management should be documented in the relevant parts of the applicant's pharmaceutical quality system (PQS) (ICH Q10).

5. Evaluation of robustness and parameter ranges of analytical procedures

5.1 Robustness

The robustness of an analytical procedure is a measure of its capacity to meet the expected performance criteria during normal use. Robustness is tested by deliberate variations of analytical procedure parameters and should consider the duration of the analysis (including stability of sample preparations and reagents). Prior knowledge and risk assessment can inform the selection of parameters to investigate during the robustness study. Those parameters likely to influence procedure performance over the intended period of use should be studied.

For most procedures, robustness evaluation is conducted during development. If the evaluation of robustness was already conducted during development, it does not need to be repeated during validation (as discussed in ICH Q2). Data from validation studies (e.g., intermediate precision) can complement robustness evaluation. For some analytical procedures with inherent high parameter variability (e.g., those requiring biological reagents) wider parameter ranges may need to be investigated during robustness studies. Robustness of multivariate procedures may require additional considerations (see Chapter 8). The outcome of the evaluation of robustness should be documented and also reflected in the analytical procedure control strategy.

5.2 Analytical procedure parameter ranges

Experiments to investigate parameter ranges can provide additional knowledge about the analytical procedure performance. The respective analytical procedure attributes and associated criteria could be derived from the ATP. Univariate examination of a single parameter can establish a PAR for the analytical procedure.

In an enhanced approach, the ranges for the relevant parameters and their interactions can be investigated in multivariate experiments (DoE). Risk assessment and prior knowledge should be used to identify analytical procedure parameters, attributes and associated ranges to be investigated experimentally. Categorical variables (e.g., different instruments) can also be considered as part of the experimental design.

The outcome of development studies should provide an understanding of the relationships between analytical procedure parameters (inputs) and the responses of the analytical procedure (outputs). Based on the results, fixed set-points may be defined for some parameters. For others, PARs could be defined while still others could be included into an MODR. An MODR consists of combined ranges for two or more analytical procedure parameters within which the analytical procedure is shown to be fit for the intended purpose.

Set-points, PARs and/or MODRs of an analytical procedure proposed by the applicant based on development and validation data are subject to regulatory approval. Moving within an approved PAR or MODR does not require regulatory notification.

Analytical procedure validation for a PAR and/or an MODR is required only for those performance characteristics not covered by data from analytical procedure development. For practical reasons and following a risk-based approach, it may not be necessary or possible to validate the entirety of an MODR. The part of a PAR or an MODR intended for routine use (typically the intended operational conditions or the set point) in the analytical procedure must be covered by validation data. The extent of validation tests should be justified on a case-by-case basis.

For future changes to operational conditions within a PAR or an MODR an assessment of the need for and extent of additional validation tests should be performed. An analytical procedure validation strategy, e.g., as part of the analytical procedure validation protocol, can define the necessary extent of additional validation.

6. Analytical procedure control strategy

An analytical procedure control strategy should ensure that the analytical procedure is fit for the intended purpose during routine use throughout its lifecycle. It consists of a set of controls, derived from current understanding of the analytical procedure including development data, risk assessment, robustness and prior knowledge. The analytical procedure control strategy should be defined before validation (ICH Q2) and should be confirmed after validation has been finalised.

The analytical procedure control strategy includes analytical procedure parameters needing control and the system suitability test (SST) which is part of the analytical procedure. The analytical procedure should describe the steps necessary to perform each analytical test. This can include (but is not limited to) the sample, the reference materials and the reagents, sample and control preparations, use of the apparatus, generation of the calibration curve, the number of replicates, use of the formulae for the calculation of the reportable results and other necessary steps. The level of detail should enable a skilled analyst to perform the analysis and interpret the results (such as the level of detail in a regional pharmacopoeia for a similar product).

The SST depends on the type and intent of the analytical procedure and is typically conducted with one or more predefined materials (including use of positive and/or negative controls). The SST is designed to verify selected analytical procedure attributes. The acceptance criteria should be based on analytical procedure performance criteria. The components of the SST should be selected using risk assessment as well as knowledge and understanding from development data. The test is used to verify that the measurement system and the analytical operations associated with the analytical procedure are fit for the intended purpose during the time period of analysis and enable the detection of unacceptable performance. Validity of the results of the analytical procedure depends on the outcome of the SST. In the enhanced approach, a well-designed set of SST parameters and criteria to ensure analytical procedure performance could represent an important aspect of risk mitigation. For analytical procedures relying on multivariate models, data quality should be verified using suitable software tools.

In addition to SST, sample suitability assessment may be required to ensure acceptable sample response. A sample and/or sample preparation is considered suitable if the measurement response of the sample satisfies pre-defined acceptance criteria for the analytical procedure attributes that have been developed for the validated analytical procedure (often used for biologics). In these cases, sample suitability is a prerequisite for the validity of the result along with a satisfactory outcome of the SST. Sample suitability assessment generally consists of the assessment of the similarity of the response between a reference material and the test sample and may include a requirement for acceptable levels of interfering signals arising from the sample matrix. For analytical procedures relying on multivariate models, sample suitability assessment can be verified using suitable software tools which check if the sample fits within the model space. This is commonly called data quality check.

Ongoing monitoring of selected analytical procedure outputs is recommended to look for any trends, in line with PQS expectations. Review of analytical procedure outputs facilitates the procedure lifecycle management and enables proactive intervention to avoid failures.

6.1 Established conditions for analytical procedures

In line with ICH Q12, applicants may propose ECs for an analytical procedure. ECs can be identified using tools highlighted in Chapter 2 including risk assessment, prior knowledge, and results from uni- and/or multi-variate experimentation. The nature and extent of ECs will depend on the development approach, the complexity of the analytical procedure and a demonstrated understanding of how parameters and other factors impact the analytical procedure performance.

With a minimal approach to analytical procedure development, the number of ECs may be extensive with fixed analytical procedure parameters and set points.

With an enhanced approach, there should be an increased understanding of the measurement requirements, the suitability of available technologies and the relationship between analytical procedure parameters and performance. This knowledge facilitates the identification of an appropriate set of ECs and related reporting categories (see Chapter 7). ECs can be reduced and focused on analytical procedure performance (e.g., acceptable ranges for analytical procedure parameters, performance characteristics with associated criteria) when justified by analytical procedure understanding (including prior knowledge and product/process knowledge) and risk management.

ECs could consist of:

- Performance characteristics and associated criteria (e.g., included in an ATP);
- Analytical procedure principle (i.e., the physicochemical basis or specific technology);
- SST and sample suitability assessment criteria;
- Set points and/or ranges for one or more analytical procedure parameters.

Analytical procedure parameters which need to be controlled to ensure the performance of the procedure as well as those where the need for control cannot be reasonably excluded should be identified as ECs. If the application of analytical procedure performance criteria and/or the SST demonstrate that a specific parameter is under control, that parameter or the parameter value may not necessarily need to be defined as an EC or may be assigned a lower reporting category as appropriate. ECs and related reporting categories are proposed by the applicant and assessed by the regulatory authorities for approval based on the scientific justification provided.

Use of the enhanced approach should not lead to providing a less detailed description of analytical procedures in a regulatory submission. Suitably detailed descriptions of the analytical procedures in Module 3 of the CTD are expected to provide a clear understanding regardless of the approach used to identify ECs for analytical procedures. A description of an analytical procedure includes supportive information as well as identified ECs.

Identification of reporting categories for ECs and the utilisation of ECs in change management are described in the next chapter.

7. Lifecycle management and post-approval changes of analytical procedures

Changes to analytical procedures can occur throughout the product lifecycle and could involve modification of existing procedures or a complete replacement including introduction of a new technology. Major changes in the performance characteristics or additional information on quality attributes could lead to reevaluation of the ATP and/or a new procedure. Typically, process knowledge, analytical procedure knowledge and continual improvement are drivers for change. If possible, changes should lead to improved analytical procedures in line with best practices and instrumentation. The tools

and enablers discussed in ICH Q12 are applicable to analytical procedures, irrespective of the development approach and consist of:

- Existing risk-based categorisation of changes to analytical procedures (in regional regulatory framework);
- ECs;
- Post-Approval Change Management Protocols (PACMPs);
- The Product Lifecycle Change Management (PLCM) document;
- The PQS (documentation of all changes including those not requiring regulatory submission);
- Structured approaches for frequent Chemistry, Manufacturing and Controls (CMC) post approval changes.

If ECs are not proposed in the dossier, any changes should be reported according to regional reporting requirements. The use of different elements of the enhanced approach can facilitate management and regulatory communication of post-approval changes as compared to the minimal approach.

Figure 2: Risk-based approach for identification of ECs and reporting categories for associated changes in the enhanced approach

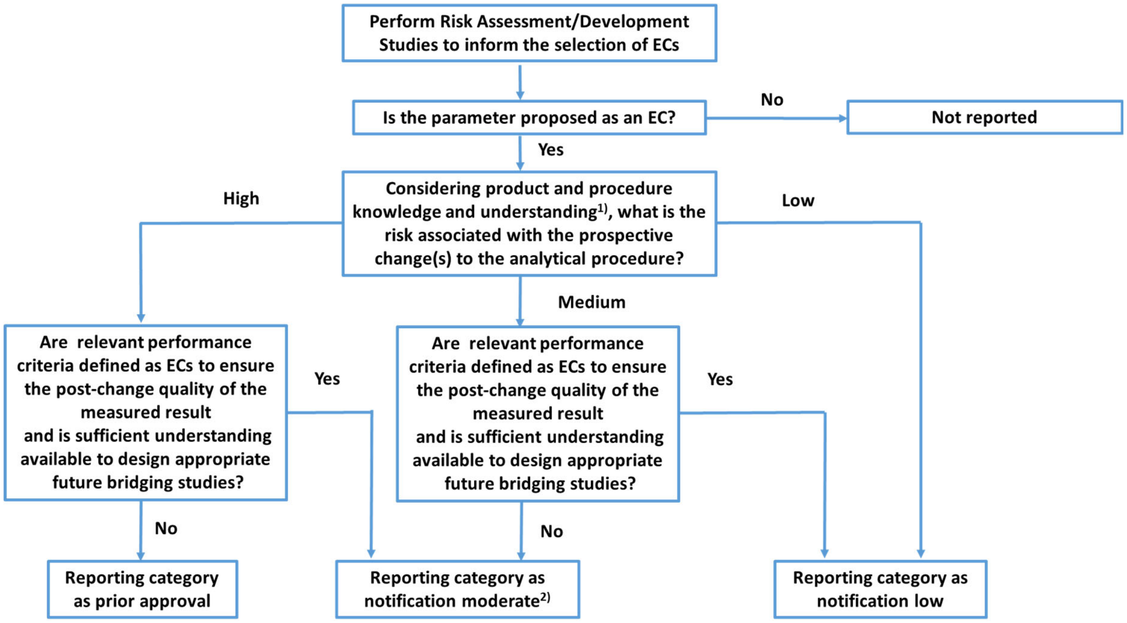

1) Including analytical procedure control strategy

2) In some cases, moderate risk changes proposed by the company may require prior approval based on health authority feedback

If justified and validated (see Chapter 5.2), a PAR or MODR allows movement within the approved range(s) to be managed within a company's PQS. Changes outside of the approved ranges or expansion of those ranges require regulatory communication.

In cases where ECs are proposed, the risk associated with prospective changes should be assessed up front to propose the appropriate reporting category. Factors to consider include the criticality of the quality attribute being measured, the complexity of the technology and the extent of the change (see

Annex A). Relevant risk reduction measures should be identified based on product and process knowledge as well as analytical procedure understanding and the proposed analytical procedure control strategy. Finally, the level of risk (high, medium or low) should be assigned.

In general, an understanding of the analytical procedure robustness and/or prior knowledge can be used to support risk mitigation associated with future changes. Submitting the outcomes of the risk assessments to regulatory authorities when ECs are proposed can help to justify reporting categories for future changes to analytical procedures.

Figure 2 summarises how risk assessment and risk reduction measures can help to identify appropriate reporting categories for ECs. The risk associated with changes can be reduced by defining relevant performance criteria which are identified as ECs. Risk reduction is possible when sufficient understanding is available (Table 1) to design future bridging studies (Table 2). Adherence to the ATP and an analytical procedure control strategy ensures that the analytical procedure remains fit for the intended purpose after changes. Changes to parameters that are not ECs do not require regulatory communication.

The ATP could also form the basis of a PACMP which would allow changes (e.g., a change between technologies) to be reported at a lower reporting category provided that the pre-defined requirements for the change are met.

Examples are provided in Annex A on how appropriate reporting categories can be proposed.

Table 1: Relationship between knowledge (understanding), risk and extent of studies for changes to analytical procedures

**Knowledge[1)]**	**Risk associated with the change**	
	Low	High
High	Prior knowledge or confirmatory study according to a study plan derived from prior knowledge	In depth study according to a study plan derived from prior knowledge
Low	Confirmatory study according to a study plan	In depth study according to a study plan

1) As described in ICH Q10

When initiating changes to analytical procedures, QRM can be used to evaluate the impact of the changes and reconfirm that the originally agreed reporting category is still appropriate. The outcome of this risk assessment informs the design and extent of the studies needed to support the change including a bridging strategy to demonstrate that the revised or new procedure remains fit for the intended purpose. When considering a bridging strategy, a greater understanding of the analytical procedure can enable a reduced study design whereas a higher risk change may need a more in-depth study (Table 1). The implementation of an already validated analytical procedure at a different location, including the concept of analytical procedure transfer, should follow a similar bridging strategy (Table 2).

For product and process changes, a reassessment and potential adaptation of the ATP, if used, and a reassessment of the suitability of the analytical procedure may be necessary.

If an applicant proposes a new analytical procedure, a comprehensive risk assessment and evaluation should be conducted to determine any impact on the performance. The analytical procedure control strategy for the new procedure should be established. ECs associated with the new procedure should be justified when communicating the change.

Table 2: Examples of analytical procedure change evaluation

**Risk Factor: Extent of change**	**Bridging strategy**	**Evidence of the suitability of a new procedure**
Change of analytical procedure principle (physicochemical/biochemical basis)	Full validation of new procedure<br>And<br>Comparative analysis of representative samples and reference materials.<br>And/or<br>Demonstration that the analytical procedure’s ability to discriminate between acceptable and non-acceptable results remains comparable	Analytical procedure performance characteristics are evaluated and criteria are met after the change<br>And<br>Results are comparable after change or differences are acceptable and potential impact on specification evaluated
Change within same analytical procedure principle	Partial or full revalidation of the analytical procedure performance characteristics affected by the change<br>And, as appropriate<br>Comparative analysis of representative samples and reference materials<br>And/or<br>Demonstration that the analytical procedure’s ability to discriminate between acceptable and non-acceptable results remains comparable	Analytical procedure attributes are evaluated and criteria are met after change<br>And, as appropriate<br>Results are comparable after change or differences are acceptable and potential impact on specification evaluated
Transfer of analytical procedure to a different site with no change in procedure itself	Partial or full revalidation of the analytical procedure performance characteristics<br>And/or<br>Comparative analysis of representative samples and reference materials<br>Or<br>Justification for not performing additional transfer experiments	Analytical procedure attributes are evaluated and criteria are met after change<br>And/or<br>Results are comparable

Table 2 provides examples of data recommended to support a change dependent on the extent of the change and the identified risk category.

To support the use of the tools described in this guideline, the company´s PQS change management process should be effective and in line with recommendations described in ICH Q12.

8. Development of multivariate analytical procedures: additional considerations

Multivariate analytical procedures are those where a result is determined through a multivariate calibration model utilising more than one input variable. The considerations provided here are for models using latent variables that are mathematically related to directly measured variables. Other approaches such as machine learning (e.g., neural networks) or optimisation techniques could use similar principles although the specific approach may vary and will not be discussed in detail.

Whereas this chapter includes aspects unique to multivariate analytical procedures, the principles described in other parts of this guideline are also applicable. Development of a robust multivariate analytical procedure includes scientifically justified sample selection and distribution over the range, sample size, model variable selection and data pre-processing.

Sample and sample population

Multivariate models link measured model variables with values obtained from a validated reference analytical procedure or from reference samples. Therefore, samples in multivariate analysis consist of input measurements and their corresponding reference values, which are numeric values for quantitative measurements (e.g., assay) and classification categories for qualitative analytical procedures (e.g., identity). In some cases, one set of input measurements could be used for multiple models provided that more than one reference value exists. The reference values are determined using reference analytical procedure(s) or prepared reference samples with known values. Care should be taken to ensure that uncertainty in the reference analytical procedure is sufficiently low in relation to the intended performance of the multivariate analytical procedure and that prepared reference samples are homogeneous. The approach to the reference analytical procedure(s) or prepared reference samples should be explained and justified.

The ranges of multivariate models are typically constructed by data from samples. Therefore, a careful strategy for sample selection is essential for obtaining the relevant information from the analytical data and contributes to the robustness of the resulting model. Based on the analytical procedure and measurement principle, the sample population should encompass the sources of variability likely to occur during manufacturing and analysis, such as raw material quality, manufacturing process variability, storage conditions, sample preparation and testing. Use of risk assessment tools can help to identify sources of variability with the potential to influence the measurements and resulting model outputs.

Obtaining samples with appropriate variability at commercial scale can be challenging. Therefore, development laboratory and pilot scale samples are often utilised to provide enough variability to improve accuracy and robustness of the model. Inclusion of commercial scale samples is recommended to capture variability related to specific equipment and/or processing conditions. Careful consideration should also be given to sample distribution in the calibration and validation sets, as this will influence the model's predictive capability.

The number of samples used to create a calibration model for quantitative analysis will depend on the complexity of the sample matrix and/or interference by the matrix in the analyte signal of interest (i.e., for more complex sample matrices generally more samples are needed).

Sufficient samples should be available for creation of calibration and validation sets of appropriate size and variability. Independent samples in the validation set are not incorporated in the calibration set or the internal test set. A validation sample set generated with samples from independent batches can be used to demonstrate model robustness.

Data transformation

The selection of the data transformation method(s) can be driven by the type of data, instrument or sample, the intended purpose of the model and/or prior knowledge. Caution should be exercised when performing any transformation because artefacts can be introduced, or essential information can be lost. Any transformation of data should be documented and justified.

Variable selection

Variable selection is performed during model development. For example, wavelength range selection is frequently applied in spectroscopic applications to select a region of a spectrum that gives the best estimation of the selected chemical or physical property to be evaluated (modelled). Variable selection depends on the measurement principle, application and other factors, and should be justified.

Robustness

Model development should minimise the prediction error and provide a robust model that consistently assures the long-term performance. The robustness should be built into the model by including relevant sources of variability related to materials, process, environment, instrumentation or other factors. Sources of variability can be identified from prior knowledge and risk assessments and evaluated using statistical tools. Robustness depends on multiple factors, e.g., composition of the calibration set, data transformation method, variable selection and the number of latent variables.

Optimisation of the multivariate model often requires a trade-off between accuracy and robustness. A critical factor is the number of latent variables to be used in the calibration model which ensures the model is optimised for the intended purpose. Selection of the number of latent variables occurs during model development and is confirmed during internal testing. Too many latent variables can result in model overfitting, potentially resulting in decreased robustness and a need for more frequent model updates. Justification for the final number of latent variables used should be provided. Diagnostic plots provided by software packages can be useful to support the justification.

Recalibration and model maintenance

Tracking the calibration model performance is an important part of ongoing monitoring for a multivariate analytical procedure. Various statistical tools can be employed as diagnostics to ensure that the model assumptions are upheld. For latent variable models, these diagnostic tools can include:

- examination of residuals to determine unmodeled features of the data (e.g., x-residuals or F-probability);
- outlier diagnostics to determine if the data is within the bounds of the model construction (e.g., Hotelling's T-squared or Mahalanobis distance).

Software packages allow for the application of diagnostic tools for model prediction.

Additionally, continued performance of the calibration model should be confirmed on a periodic and event-driven basis by comparison of the model predictions with the known values of the reference samples or reference analytical procedure results. This confirmatory testing helps to ensure that the calibration model continues to perform as expected. Examples of events that could trigger confirmatory testing include new known process variability, unexpected process events or scheduled instrument maintenance.

Monitoring of the model can be used to trigger model rebuilding (recalibration) as a part of continual improvement. In general, the same considerations hold as for the original model building and internal testing. Based on the cause of the model update (*e.g.,* a process shift), new data may need to be included and old non-relevant data may be taken out.

Once the new calibration model is established, the updated analytical procedure can be validated against the same performance criteria as the ones included in the original model. Aspects that are not expected to change from the model update may not need to be evaluated (e.g., specificity).

Figure 3: Multivariate (MV) model lifecycle

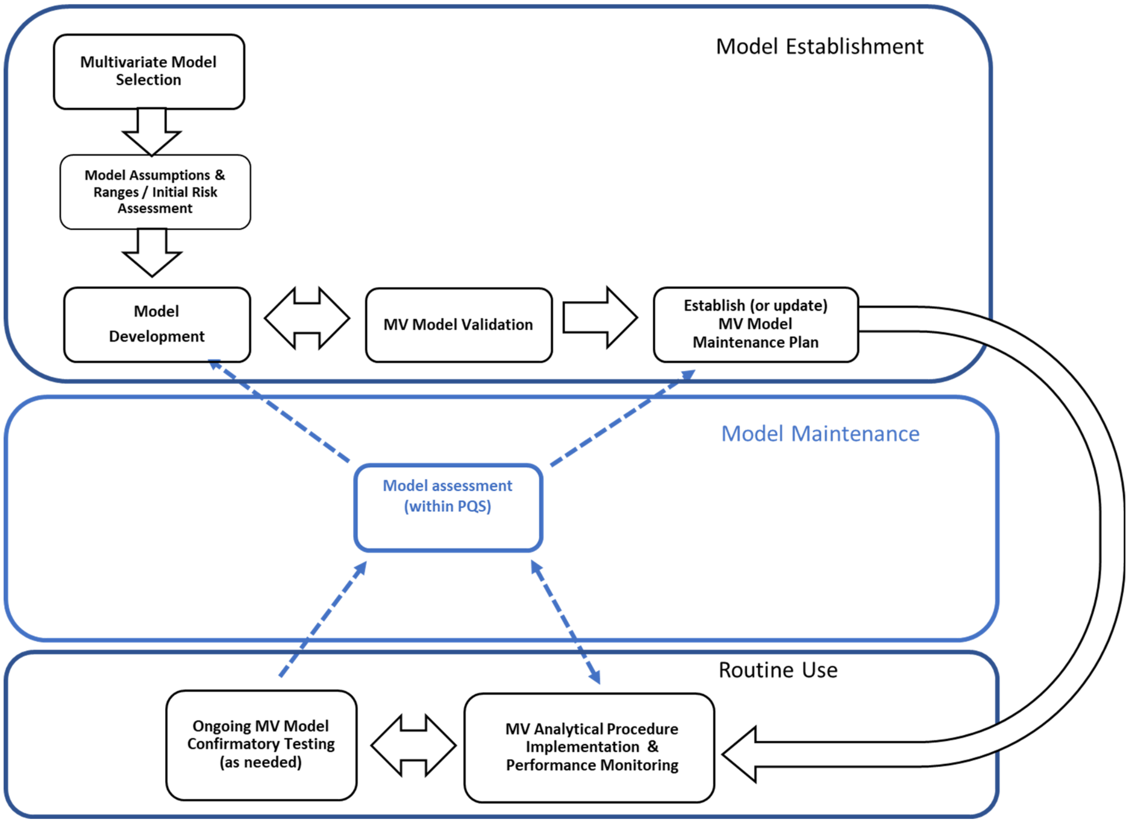

The multivariate model lifecycle (Figure 3) is iterative and can be broken down into 3 major components: model establishment, routine use and model maintenance.

The choice of a multivariate model is based on the analytical procedure requirements and the measurement technology selected. Prior to model development, the performance factors for the model are defined, including the underlying model assumptions and desired ranges for model applicability. An initial risk assessment can be valuable to understand potential sources of variability in the materials and process that could affect the model performance and therefore should be considered during the model calibration. Model development, including calibration and internal testing, follows the considerations outlined in this chapter. Once the model is developed, it is validated using independent data not previously used in the calibration set. The last step in model establishment is development of a multivariate model maintenance plan, which includes the procedures and limits for outlier diagnostics, and defines the frequency and circumstances for confirmatory testing, if needed.

Routine analysis of the multivariate analytical procedure typically includes monitoring the appropriateness of every measurement using outlier diagnostics. Model reassessment can be triggered by failure of confirmatory testing or outlier diagnostics to meet the predefined criteria, or from data trending indicating potential unacceptable performance of the model, the process or the materials being measured (examples of multivariate model lifecycle components are provided in Annex B).

Model reassessment is performed within the PQS and utilises knowledge management and risk assessment. If unacceptable performance is identified, model development and revalidation may be needed, for example, to add samples into the calibration set and remove those that are no longer relevant. In some cases, the model may be performing appropriately, but additional experience may identify the need to modify the model maintenance plan. In other cases, the identified unacceptable performance could be related to the measurement system (e.g., a misaligned sample interface) and no model update would be needed. The dashed arrows in Figure. 3 illustrate reintroduction into the lifecycle flow based on the potential outcomes of the model reassessment.

9. Development of analytical procedures for real time release testing: additional considerations

Real Time Release Testing (RTRT) is the ability to evaluate and ensure the quality of in-process and/or final product based on process data, which typically include a valid combination of measured material attributes and process controls (ICH Q8). RTRT measurements work in conjunction with all elements of the product control strategy (e.g., process monitoring or in-process controls) to ensure product quality. RTRT can be applied to drug substances, intermediates and drug products.

RTRT can be based on an appropriate combination of one or more process measurements and/or material attributes to provide a value for one or more CQAs and should be specific for those CQAs. The relationship between the RTRT approach and the CQAs, as well as acceptance criteria, should be fully justified. An RTRT analytical procedure should be validated as recommended in ICH Q2 and it should be demonstrated that the process measurements have appropriate specificity for the targeted quality attribute.

Sampling and the sample interface are important considerations when designing any on-line or in-line analytical procedure, including those used for RTRT. The measurement point(s) should be chosen to be representative of the entire material being processed with the sample duration or amount appropriately chosen (e.g., relative to a unit dose). Additionally, the sample interface should remain consistent over the duration of manufacturing and should be robust to expected processing and environmental variations.

The RTRT approach should be included in the product specification (ICH-Endorsed Guide for ICH Q8/Q9/Q10 Implementation). In accordance with ICH Q6A Specifications: Test Procedures and Acceptance Criteria For New Drug Substances and New Drug Products: Chemical Substances and ICH Q6B Specifications: Test Procedures and Acceptance Criteria For Biotechnological/Biological Products, this includes a reference to the analytical procedure(s) and the related acceptance criteria. Quantitative RTRT results should be expressed in the same units as those for traditional testing. The product specification will typically also include the analytical procedures to be used for off-line testing. If the dossier includes a registered alternative control strategy to RTRT (e.g., traditional end-product testing when process analytics are unavailable), the related analytical procedures and when they would be applied should also be included in the product specifications.

10. Submission of analytical procedure related information

10.1 General regulatory considerations and documentation

The analytical procedure description(s) should be included in the ICH M4Q CTD section 3.2.S.4.2 for drug substance or section 3.2.P.5.2 for drug product. Validation data and supportive information needed to justify the analytical procedure control strategy should be included in the CTD section 3.2.S.4.3 for drug substance or section 3.2.P.5.3 for drug product. Other analytical procedures used as part of the control strategy should be included in relevant CTD sections (e.g., 3.2.S.2, 3.2.P.3 and 3.2.P.4). The analytical procedure should describe the steps in sufficient detail for a skilled analyst to perform the analysis (including SST) as elaborated in Chapter 6. Submission of validation data should reflect the guidance provided in ICH Q2. The performance criteria used in the validation study should be included in the submission. In some cases, depending on the intended purpose (e.g., dissolution testing) and/or the selected technique, it may be appropriate to submit development data as justification.

Where ECs are proposed for analytical procedures as elaborated in Chapter 6, the ECs should be clearly differentiated from supportive information. Additional development and validation information can be included in sections 3.2.S.4.3 and 3.2.P.5.3 to justify ECs and their reporting categories. When other lifecycle management elements as described in ICH Q12 are included in the submission, the applicant should follow the principles described in ICH Q12 and Chapter 7 of this document.

10.2 Documentation for the enhanced approach

If the approach to development leads to the incorporation of enhanced elements into the analytical procedure control strategy, then these should be justified.

Performance characteristics and acceptance criteria (e.g., described in an ATP) and other elements of the enhanced approach (e.g., MODRs or PARs), should be described in the dossier sections for analytical procedure description (e.g., 3.2.S.4.2 and 3.2.P.5.2). If ECs are proposed, then these should also be included in the analytical procedure description, accompanied by supportive information. The use of the enhanced approach should not lead to providing a less detailed description of analytical procedures in a regulatory submission.

If ECs and related reporting categories are proposed, risk-based categorisation of changes and corresponding reporting categories should be included in the submission. Justification should be given for parameters that are ECs and those that are not ECs, as appropriate (see Chapter 6).

Appropriate information from analytical procedure risk assessment and development studies to support the proposed lifecycle management strategy should be summarised and submitted in the regulatory submission sections for analytical procedure validation (e.g., 3.2.S.4.3 and 3.2.P.5.3).

10.3 Documentation for multivariate analytical procedures

Development information related to multivariate analytical procedures should be provided commensurate with the level of impact of the model (ICH-Endorsed Guide for ICH Q8/Q9/Q10 Implementation). The process development section of the dossier (*e.g.,* 3.2.S.2.6 or 3.2.P.2) should include the model development information for multivariate models used as part of manufacturing development studies or for in-process controls or tests. Supportive development information for RTRT multivariate models can be included in either the appropriate analytical procedure validation or process development section.

Validation information for analytical procedures used for release of drug substance or drug product, including RTRT, should be included in the validation information section of the dossier (*e.g.,* 3.2.S.4.3 or 3.2.P.5.3). Additionally, these sections should include validation information on analytical procedures used as reference analytical procedures. The model development, calibration and validation information can be included directly in the CTD section or in an appended document.

For multivariate models used as part of drug substance or drug product specifications, including RTRT approaches, the description of the validation approach and results should include:

- Description of the validation set with independent samples;
- The performance criteria to be met during validation of the multivariate model;
- Evaluation of the model validation results against the performance criteria;
- Discussion of the relationship between the model performance criteria and the attribute specification limits;
- High level overview of the PQS elements for model monitoring and maintenance, such as diagnostic tools for determining the appropriateness of the sample data for the model and the approach taken when outliers are identified.

The description of the analytical procedure used for RTRT should be provided in the CTD section 3.2.S.4.2 for drug substance or section 3.2.P.5.2 for drug product and typically includes:

- The property or attribute of interest to be determined by the multivariate analytical procedure and the desired quantitative ranges or limits;
- A description of the measurement principle and pertinent instrument operating parameters (e.g., sample presentation, sample interrogation time and measurement frequency);
- An overview of how the multivariate model calibration data are obtained (e.g., sample preparation approach, reference analytical procedure);
- The type of multivariate model;
- A description of reference analytical procedure or high-level description of prepared reference samples;
- Any calculations needed to adjust the model output into the reported value.

Additionally, section 3.2.S.4.2 for drug substance or section 3.2.P.5.2 for drug product should include description of any analytical procedures that are part of a registered alternative control strategy to RTRT. Information on validation of these alternative analytical procedures should be included in the validation information section of the dossier (*e.g.,* 3.2.S.4.3 or 3.2.P.5.3).

11. Glossary

Accuracy

The accuracy of an analytical procedure expresses the closeness of agreement between the value which is accepted either as a conventional true value or as an accepted reference value and the value or set of values measured. (ICH Q2)

Analytical procedure

The analytical procedure refers to the way of performing the analysis. The analytical procedure should describe in sufficient detail the steps necessary to perform each analytical test. (ICH Q2)

Analytical procedure attribute

A technology specific property that should be within an appropriate limit, range, or distribution to ensure the desired quality of the measured result. For example, attributes for chromatography measurements may include peak symmetry factor and resolution. (ICH Q14)

Analytical procedure control strategy

A planned set of controls derived from current analytical procedure understanding that ensures the analytical procedure performance and the quality of the measured result. (ICH Q14)

Analytical procedure parameter

Any analytical factor (including reagent quality) or analytical procedure operational condition that can be varied continuously (e.g., flow rate) or specified at controllable, unique levels. (ICH Q14)

Analytical procedure validation strategy

An analytical procedure validation strategy describes the selection of analytical procedure performance characteristics for validation. In the strategy, data gathered during development studies and system suitability tests (SSTs) can be applied to validation and an appropriate set of validation tests can be predefined. (ICH Q14)

Analytical target profile (ATP)

A prospective summary of the performance characteristics describing the intended purpose and the anticipated performance criteria of an analytical measurement. (ICH Q14)

Calibration model

A model based on analytical measurements of known samples that relates the input data to a value for the property of interest (i.e., the model output). (ICH Q2)

Control strategy

A planned set of controls, derived from current product and process understanding, that assures process performance and product quality. The controls can include parameters and attributes related to drug substance and drug product materials and components, facility and equipment operating conditions, in-process controls, finished product specifications, and the associated methods and frequency of monitoring and control. (ICH Q10)

Critical quality attribute (CQA)

A physical, chemical, biological or microbiological property or characteristic that should be within an appropriate limit, range or distribution to ensure the desired product quality. (ICH Q8)

CTD
Common Technical Document. (ICH M4Q)

DoE
Design of Experiments.

Established conditions (ECs)

ECs are legally binding information considered necessary to assure product quality. As a consequence, any change to ECs necessitates a submission to the regulatory authority. (ICH Q12)

Intermediate precision

Intermediate precision expresses intra-laboratory variations. Factors to be considered should include potential sources of variability, for example, different days, different environmental conditions, different analysts and different equipment. (ICH Q2)

Knowledge management

A systematic approach to acquiring, analysing, storing and disseminating information related to products, manufacturing processes and components. (ICH Q10)

Method operable design region (MODR)

A combination of analytical procedure parameter ranges within which the analytical procedure performance criteria are fulfilled and the quality of the measured result is assured. (ICH Q14)

Ongoing monitoring

The collection and evaluation of analytical procedure performance data to ensure the quality of measured results throughout the analytical procedure lifecycle. (ICH Q14)

PACMP
Post-Approval Change Management Protocol. (ICH Q12)

Performance characteristic

A technology independent description of a characteristic that ensures the quality of the measured result. Typically, accuracy, precision, specificity/selectivity and range may be considered. Previous ICH Q2 versions referred to this as VALIDATION CHARACTERISTIC. (ICH Q2)

Performance criterion

An acceptance criterion describing a numerical range, limit or desired state to ensure the quality of the measured result for a given performance characteristic. (ICH Q14)

Platform analytical procedure

An analytical procedure that is suitable to test quality attributes of different products without significant change to its operational conditions, system suitability and reporting structure. This type of analytical procedure can be used to analyse molecules that are sufficiently alike with respect to the attributes that the platform analytical procedure is intended to measure. (ICH Q2)

Precision

The precision of an analytical procedure expresses the closeness of agreement (degree of scatter) between a series of measurements obtained from multiple samplings of the same homogeneous sample under the prescribed conditions. Precision can be considered at three levels: repeatability, intermediate precision and reproducibility.

The precision of an analytical procedure is usually expressed as the variance, standard deviation or coefficient of variation of a series of measurements. (ICH Q2)

Proven acceptable range for analytical procedures (PAR)

A characterised range of an analytical procedure parameter for which operation within this range, while keeping other parameters constant, will result in an analytical measurement meeting relevant performance criteria. (ICH Q14)

Quality risk management (QRM)

A systematic process for the assessment, control, communication and review of risks to the quality of the drug (medicinal) product across the product lifecycle. (ICH Q9)

Range

The range of an analytical procedure is the interval between the lowest and the highest results in which the analytical procedure has a suitable level of precision, accuracy and response. (ICH Q2)

Reportable range

The reportable range of an analytical procedure includes all values from the lowest to the highest reportable result for which there is a suitable level of precision and accuracy. Typically, the reportable range is given in the same unit as the specification acceptance criterion. (ICH Q2)

Working range

A working range corresponds to the lowest and the highest level of the quality attribute to be measured (e.g., content or purity) as presented to the analytical instrument and for which the analytical procedure provides reliable results. (ICH Q2)

Real time release testing (RTRT)

The ability to evaluate and ensure the quality of the in-process and/or final product based on process data, which typically include a valid combination of measured material attributes and process controls. (ICH Q8)

Reference material

A suitably characterised material, sufficiently homogeneous and stable with regard to one or more defined attributes, which has been established to be fit for the intended purpose. Reference materials may include national/international reference standards, pharmacopoeial reference standards, or in-house primary/secondary reference materials. (ICH Q2)

Repeatability

Repeatability expresses the precision under the same operating conditions over a short interval of time. Repeatability is also termed intra-assay precision. (ICH Q2)

Reportable result

The result as generated by the analytical procedure after calculation or processing and applying the described sample replication. (ICH Q2)

Reproducibility

Reproducibility expresses the precision between laboratories (e.g., inter-laboratory studies, usually applied to standardisation of methodology). (ICH Q2)

Response

The response of an analytical procedure is its ability (within a given range) to obtain a signal which is effectively related to the concentration (amount) or activity of analyte in the sample by some known mathematical function. (ICH Q2)

Revalidation

Demonstration that an analytical procedure is still fit for the intended purpose after a change to the product, process or the analytical procedure itself. Revalidation can involve all (full revalidation) or a subset (partial revalidation) of performance characteristics. (ICH Q2)

Robustness

The robustness of an analytical procedure is a measure of its capacity to meet the expected performance criteria during normal use. Robustness is tested by deliberate variations of analytical procedure parameters. (ICH Q14)

Sample suitability assessment

A sample or sample preparation is considered suitable if the measurement response on the sample satisfies pre-defined acceptance criteria for the analytical procedure attributes that have been developed for the validated analytical procedure. (ICH Q14)

Specificity/selectivity

Specificity and selectivity are both terms to describe the extent to which other substances interfere with the determination of an analyte according to a given analytical procedure. Specificity is typically used to describe the ultimate state, measuring unequivocally a desired analyte. Selectivity is a relative term to describe the extent to which particular analytes in mixtures or matrices can be measured without interferences from other components with similar behaviour. (ICH Q2)

System suitability test (SST)

System suitability tests are developed and used to verify that the measurement system and the analytical operations associated with the analytical procedure are fit for the intended purpose and increase the detectability of unacceptable performance. (ICH Q14)

Validation study

An evaluation of prior knowledge, data or deliberate experiments (i.e., validation tests) to determine the suitability of an analytical procedure for the intended purpose. (ICH Q2)

Validation test

Validation tests are deliberate experiments designed to authenticate the suitability of an analytical procedure for the intended purpose. (ICH Q2)

Multivariate glossary

Calibration set

A set of data with matched known characteristics and measured analytical results. (ICH Q14)

Data transformation

Mathematical operation on model input data to assume better correlation with the output data and to simplify the model structure. (ICH Q14)

Independent sample

Independent samples are samples not included in the calibration set of a multivariate model. Independent samples can come from the same batch from which calibration samples are selected. (ICH Q2)

Internal testing

Internal testing is a process of checking if unique samples processed by the model yield the correct predictions (qualitative or quantitative).

Internal testing serves as means to establish the optimal number of latent variables, estimate the standard error and detect potential outliers. (ICH Q2)

Internal test set

A set of data obtained from samples that have physical and chemical characteristics that span a range of variabilities similar to the samples used to construct the calibration set. (ICH Q14)

Latent variables

Mathematically derived variables that are directly related to measured variables and are used in further processing. (ICH Q2)

Model maintenance

The process of ensuring continued model performance over the lifecycle of a multivariate model, which often includes outlier diagnostics and resulting actions for model redevelopment or change in the maintenance plans. (ICH Q14)

Model validation

The process of determining the suitability of a model by challenging it with independent test data and comparing the results against predetermined performance criteria. (ICH Q2)

Multivariate analytical procedure

An analytical procedure where a result is determined through a multivariate calibration model utilising more than one input variable. (ICH Q2)

Outlier diagnostic

Tests that can identify unusual or atypical data in a multivariate analytical procedure. (ICH Q14)

Reference analytical procedure

A separate analytical procedure used to obtain the reference values of the calibration and validation samples for a multivariate analytical procedure. (ICH Q2)

Reference sample

A sample representative of the test sample with a known value for the property of interest, used for calibration. (ICH Q14)

Validation set

A set of data used to give an independent assessment of the performance of the calibration model. (ICH Q2)

12. REFERENCES

ICH Q2 Validation of Analytical Procedures

ICH Q6A Specifications: Test Procedures and Acceptance Criteria For New Drug Substances and New Drug Products: Chemical Substances

ICH Q6B Specifications: Test Procedures and Acceptance Criteria For Biotechnological/Biological Products

ICH Q8 Pharmaceutical Development

ICH Q9 Quality Risk Management

ICH Q10 Pharmaceutical Quality System

ICH Q11 Development and Manufacture of Drug Substances

ICH Q12 Technical and Regulatory Considerations for Pharmaceutical Product Lifecycle Management

ICH M4Q The Common Technical Document For The Registration of Pharmaceuticals For Human Use

ICH-Endorsed Guide for ICH Q8/Q9/Q10 Implementation

13. ANNEX

13.1 Annex A: Examples of application of ICH Q14 principles

The examples provided in this Annex are for illustrative purposes only. Other approaches are possible. They suggest how the concepts described in ICH Q14 could be applied and should not be used as a template or the sole basis for a regulatory submission.

The examples have been created to illustrate:

- How analytical procedure performance characteristics, derived from the product context and knowledge, could be summarised in an ATP;
- How performance characteristics described in the ATP could be applied to select a suitable analytical technology, guide the development of an analytical procedure and help define the analytical procedure control strategy;
- How to identify ECs for analytical procedures developed using elements of the enhanced approach;
- How QRM and the adherence to associated criteria for relevant performance characteristics and/or the subsequent execution of a bridging study can ensure the post-change quality of the measured result and help to justify the respective reporting categories for ECs and the post approval change management of analytical procedures.

As described in Chapter 4 of ICH Q14, QRM can be used to evaluate the impact of prospective changes for analytical procedures. The list below describes examples of risk factors and risk reduction measures to identify the risks associated with the changes to an analytical procedure. The outcome of the risk assessment (risk level: high, medium or low) feeds into the design and extent of the studies needed to support the change.

Selected risk (risk factors)

- Relevance of the test
    - Potential clinical impact of the measured attribute (efficacy, safety, pharmacokinetics and immunogenicity), e.g., controlling CQA vs. non CQA;
    - Extent of knowledge of the attribute;
    - Attribute ensured by other elements of the control strategy (testing or process control);
- Complexity of the technology
    - Platform technologies;
    - Novel vs. established technology (e.g., in pharmacopoeias);
    - Several attributes reported as a sum (e.g., charge variants for large molecules);
    - Biological assays, cell-based assays, immunochemical assays;
    - Multi-attribute analytical procedure;
    - Multivariate analytical procedure;
- Extent of the change
    - Change of one or several parameters outside the already proven acceptable ranges;

- Change of the analytical procedure within existing analytical procedure performance characteristics and associated criteria;
- Change to a new analytical procedure using a different technology;
- Change to analytical procedure performance criteria (e.g., due to tightening a specification limit).

Risk reduction

Risk reduction is defined in ICH Q9 as actions taken to lessen the probability of occurrence of harm and the severity of that harm.

Different kinds of knowledge can lead to reduction of risk, for example:

- Product and process knowledge
    - Knowledge about quality attributes of the drug substance/drug product and acceptable ranges of CQAs;
    - Well justified analytical procedure performance criteria cover/link to CQAs and their acceptable ranges;
    - Evidence to control the CQAs through the process parameter settings
    - Knowledge of the degradation pathways demonstrated by the analysis of relevant stressed samples;
    - Other product knowledge (e.g., impurity profile, particle size and distribution);
- Analytical procedure understanding and analytical procedure control strategy
    - Knowledge about analytical procedure parameters and their impact on measurement performance;
    - Proven analytical procedure robustness, e.g., harmonised procedures (compendial tests);
    - Enhanced analytical procedure understanding (e.g., DoE studies) supporting justification of acceptable ranges (e.g., PAR, MODR);
    - Other knowledge from development of analytical procedure;
    - System Suitability Test ensures relevant analytical procedure attributes;
    - Ongoing monitoring of analytical procedure output;
    - Clear link between signal and CQA to be measured (e.g., peak characterisation available, specificity);
- Bridging strategy for changes to analytical procedures
    - Availability of reference material, relevant historical and or stressed samples to support analytical procedure output assessment against performance criteria (demonstrated ability to control the CQA);
    - Comparison to output of previous analytical procedure (understanding and acceptance of risk for potential differences);
    - Demonstrated understanding of risks associated with parameter changes and potential interactions with other parameters;

- Prior experience with similar changes, analytes or technologies including platform analytical procedures.

13.1.1 Measurement of stereoisomers as specific process related impurities in a small molecule drug substance (DS)

Introduction and Background

"Sakuratinib Maleate" is a small molecule DS with multiple chiral centres. The chirality of the molecule, its degradation pathway and the impurities are well characterised. From this knowledge and the established manufacturing process controls, the six stereoisomers (Impurity A-F) were found to be potentially present in the final product.

Table 1: Analytical target profile

<table>
<tr><td colspan="3">Intended Purpose</td></tr>
<tr><td colspan="3">Quantitation of the six stereoisomers A-F in Sakuratinib Maleate DS for release testing</td></tr>
<tr><td colspan="3">Link to CQA (Stereoisomeric Purity)</td></tr>
<tr><td colspan="3">The analytical procedure should allow for the quantitation of the individual stereoisomers A-F and determination of the total sum to verify the CQA Stereoisomeric Purity ≥99.0%</td></tr>
<tr><td colspan="3">Characteristics of the Reportable Results</td></tr>
<tr><td>Performance Characteristics</td><td>Acceptance Criteria</td><td>Rationale</td></tr>
<tr><td>Accuracy</td><td>80–120% average recovery of spiked DS with Impurities A-E (specified at NMT 0.1% each)

90–110% average recovery of spiked DS with Impurity F (specified at NMT 0.5%)</td><td rowspan="2">For example, at a specification level of 0.1%, 20% bias would lead to a variation of the analytical result of 0.02%, which was found acceptable for a release decision.

In a similar fashion, values for precision were derived. The recovery criteria for accuracy were set with respect to the reported result and taking into consideration any correction or response factors</td></tr>
<tr><td>Precision</td><td>Intermediate Precision RSD:

Impurities A–E ≤15%

Impurity F ≤10%</td></tr>
<tr><td>Specificity</td><td>Analytical procedure should be able to quantitate impurities A–F in presence of other likely process related substances or DS degradation products with an acceptable bias of not more than 0.02%</td><td>Potential interference with quantitation of specified impurities by other regular components in the sample</td></tr>
<tr><td>Reportable Range</td><td>Impurities A–E: at least 0.05–0.12%

Impurity F: at least 0.05–0.6%</td><td>Reporting threshold to 120% of specification limit</td></tr>
</table>

Technology Selection

Multiple analytical technologies are available for the separation of stereoisomers. In this example, HPLC was chosen because development studies showed good potential for separation of stereoisomers. As detection mode, UV detection was selected as it was known that the molecule had sufficient UV absorption properties.

Analytical Procedure Development

The chiral HPLC procedure for quantitation of stereoisomers was developed using enhanced principles. Below is a summary of the activities conducted during enhanced development.

- An understanding of the chemistry, process, and impurities that have potential to be present in the drug substance was established;
- Reference materials were made available for development and validation;
- Conducted risk assessment and evaluating prior knowledge to identify the analytical procedure parameters that can impact performance of the procedure;
- Conducted modelling and multi-variate experiments including robustness testing to explore ranges and interactions between identified analytical procedure parameters;
- Defined analytical procedure control strategy based on procedure understanding including set-points for relevant analytical procedure parameters and SST.

Analytical Procedure

For the purpose of this example, a summary of the analytical procedure is provided below. This does not reflect the entirety of the analytical procedure description in the dossier.

Table 2: Summary of the analytical procedure description

Column	Chiral Column, Amylose tris-(3,5-dimethylphenylcarbamate), immobilised on porous, spherical, silica particles, 4.6 mm ID x 250 mm, 3 µm
Mobile Phase	*n*-hexane / ethanol / TFA (80/20/0.1)
Flow Rate	1 mL/min
Column temperature	30°C
Detection	UV 214 nm
Injection Volume	5 µL
Standard/Sample Concentration	1.0 mg/mL
System Suitability Tests	*Controlled Parameters*
Resolution between critical peak pair: DS Main Peak and Impurity D ≥ 2.0	Column, Temperature*, Mobile Phase, Flow Rate
S/N at QL; DS at 0.05% >10	Injection Volume, Column, Mobile Phase, Standard/Sample Concentration, Detection Wavelength
Repeatability of injection of DS at 0.5% level ≤ 5%	Injection Volume, Mobile Phase

* For example, the retention time models built from data collected during analytical procedure development screens were used to assess the robustness of temperature and other parameters, that could potentially affect the performance characteristics (e.g., specificity). The in silico robustness was verified experimentally by confirming resolution at the centre point and design points that generated the minimum and maximum main peak retention time.

Analytical Procedure Validation

After the analytical procedure development was finalised and the analytical procedure control strategy established, a validation study was planned and completed according to the ICH Q2 guideline.

Description of Established Conditions (ECs), Reporting Categories, and Justifications

The applicant proposed and justified established conditions and reporting categories, as part of the submission. For the purpose of this example, Table 3 describes the proposed ECs, their proposed reporting categories and examples of parameters that are not ECs.

Note: The extent of ECs and associated reporting categories listed in this table depend on the extent of knowledge gained, information and justification provided in the dossier. The dossier is subject to regulatory review. The information provided in this example is only part of the knowledge available that will be submitted and is provided for illustrative purposes only. The extent of ECs (EC or not EC designation), actual reporting categories, and data requirements may differ by region. Depending on the nature and extent of the change (e.g., change to a different technology), a PACMP may be required.

Table 3: Evaluated risk, proposed established conditions and proposed reporting categories

**Established Condition**	**Overall Risk Category**	**Proposed Reporting Category**[1)]	**Comment**
Performance Characteristics and Criteria as described in the ATP: Accuracy, Precision, Specificity, Range (see Annex A , Table 1)	High	PA	The performance characteristics and criteria ensure the quality of the reportable result and link to the CQA.<br><br>If widening of the performance criteria is necessary, it will be reported as PA
Technology: Chiral Liquid Chromatography<br><br>Suitable chiral separation technique to meet performance characteristics defined in ATP	Medium	NM	A technique that meets the performance characteristics and criteria ensures the quality of the reportable result and link to the CQA.<br><br>There is a strong understanding between product knowledge, intended purpose, and the analytical procedure performance established to enable the design of future bridging studies.<br><br>A change resulting in a widening of the specification acceptance criteria might require a higher reporting category
System Suitability Test and parameter-control relationship (see Annex A, Table 2)	Medium	NL/NM	SST was developed for the LC procedure based on a risk analysis and ensures adherence to the performance characteristics and criteria. Control relationships were established through prior knowledge (general principles of technique) and during procedure development.<br><br>If the SST criteria are widened the reporting category would be higher
LC Column: Amylose tris-(3,5-dimethylphenylcarbamate), immobilised on porous, spherical silica particles<br><br>Mobile Phase Components:<br><br>*n*-Hexane, Ethanol, TFA<br><br>Method of detection: UV 214 nm	Low	NL/NM	The LC column, mobile phase components and mode of detection are the main parameters, defining the separation mechanism and detection. Changing these parameters may result in the need to adapt the SST
**The other analytical procedure parameters defined as ECs are omitted for the purpose of this example**			
**The following conditions are examples of parameters that are not ECs**[2)]**:**			

Established Condition	Overall Risk Category	Proposed Reporting Category[1)]	Comment
Ratio of mobile phase components: n-Hexane/Ethanol/TFA (80/20/0.1)<br>Instrumental conditions:<br>Temperature: 30°C<br>Column length, packing particle size	Low	-	These parameters are controlled by the SST. Robustness testing supported by modelling was performed at the centre point and the extrema that generated the minimum and maximum main band retention time
Preparation of test solutions and reference materials: 1 mg/mL DS in mobile phase	Low	-	The performance over the working range has been demonstrated through the linearity experiments during validation

1) PA: Prior Approval, NM: Notification Moderate; NL: Notification Low (as per ICH Q12 definitions)

2) Depending on the region, some of this information is included in an approval letter

Change Management and Bridging Strategy

The change described below is an example of one that could occur during the lifecycle of a product and analytical procedure. When the product was initially submitted and approved, Supercritical Fluid Chromatography (SFC) was not selected as the analytical technique due to unavailability in the commercial facility. Years after approval, the applicant desires to change the technique to SFC as it is a more environmentally-friendly technology and is now available at the commercial site.

For this change, following development of the SFC procedure the applicant will perform a structured risk assessment to evaluate potential impact on the performance characteristics and the link to CQA (stereoisomeric purity) as defined in the ATP. As an outcome of the risk assessment, experimental bridging studies to demonstrate adherence to the performance characteristics and associated criteria will be performed. Validation of the new analytical procedure and comparative analysis of representative samples and reference materials will be performed.

The applicant should not implement the new analytical procedure using the predefined reporting category unless adherence to the performance characteristics and associated criteria defined in the ATP is demonstrated during the bridging studies. If the precondition of adherence to the ATP cannot be met, a higher reporting category would apply.

The example in Annex A, Figure 1 illustrates a post-approval change in technique as well as the steps an applicant would follow when implementing the change. The information in the table above (ECs and reporting categories) would need to be agreed upon up front with the regulatory authority.

Figure 1: Example of work process of applicant to change an approved analytical procedure

Post-approval Change
Technique from chiral HPLC to chiral SFC

Reassessment of the risk of the change with the following points considered

- **Relevance of the test:** High, control of the CQA (quantitation of stereoisomers)
- **Complexity of the test:** well-established technology
- **Extent of the change:** related technologies; prior knowledge of chemistry, process and impurities informs the procedure development

Estimated risk: High

Development approach for chiral SFC procedure

- **Enhanced development approach of the same principles as completed for the HPLC procedure was followed**
- **Risk assessment was conducted to identify the analytical procedure parameters that can impact performance of the procedure**
- **Modelling and/or multi-variate experiments were performed to explore ranges and interactions between identified analytical procedure parameters**
- **An analytical procedure control strategy was defined**

Re-confirmation of the following points

- **Adherence to criteria for relevant performance characteristics:** Those are defined as ECs
- **Sufficient information or prior knowledge to design appropriate bridging studies:** Yes

Risk assessment result before bridging study execution is in agreement with the submitted risk category.
Overall risk category: Medium

Execution of analytical procedure development

- **Procedure parameters for chiral SFC were determined through knowledge of the process and impurities, and enhanced practices for development**
- **Analytical procedure description (including AP control strategy (SST)) was finalized**

Demonstration of analytical procedure performance

- **The procedure was validated by a technology specific validation protocol**
- **Through above studies, relevant performance characteristics and the analytical procedure attributes were evaluated and confirmed**
- **Execution of bridging study (ICH Q14 Table 2)**

Conclusions

The impact of the change was determined based on the bridging study result

- **Impact on the test performance:** The relevant performance characteristics and the analytical procedure attributes met their criteria
- **Bridging study results met all acceptance criteria**

Regulatory reporting

Reported according to the pre-agreed reporting category in registration as notification moderate and submitted suitable documents.

13.1.2 Measurement of potency for an anti-TNF-alpha monoclonal antibody

Introduction and Background

The example presented refers to the measurement of the relative potency of the drug, in this case an anti-TNF-alpha monoclonal antibody, in drug substance and in drug product at release and for stability testing.

Assumptions for the example:

- Mode of action: the neutralisation of the biological activity of soluble TNF-alpha by preventing TNF-alpha from binding to the TNF-alpha receptor;
- Fc-effector functions are out of scope;
- Specification limits for the relative potency: 80% to 125% compared to reference material;
- Potency assay to be developed is able to detect a change and/or a shift in potency upon forced degradation.

Table 4: Analytical target profile

**Intended Purpose**		
Measurement of the potency of an anti-TNF-alpha monoclonal antibody in drug substance and in drug product at release and for stability testing.		
**Link to CQA (biological activity)**		
The mode of action of the drug is the neutralisation of the biological activity of soluble TNF-alpha by preventing TNF-alpha from binding to the TNF-alpha receptor. Target acceptance criteria: 80% to 125% relative potency[1)]		
**Characteristics of the Reportable Result**		
**Performance Characteristics**	**Acceptance criteria**	**Rationale**
Accuracy	Accuracy is assessed via a linearity experiment that covers the reportable range. No trend in relative bias is observed over the tested relative potency range<br>The 95% confidence interval of the slope of the fitted regression line between theoretical and measured potency falls within a range of 0.8 to 1.25<br>The upper and lower 90% confidence interval for the relative bias calculated at each potency level is not more than 20%[1)]	Parameters are assessed based on compendial guidance<br>The acceptance criteria are determined considering the intended purpose of the measurement<br>Selected performance characteristic ensures that the intended analytical procedure delivers the quality of the reportable result
Precision	Upper 95% confidence interval for the average intermediate precision across levels across the reportable range (95% CI % geometric coefficient of variation) is not more than 20%[1)]	

Specificity	Analytical procedure is specific for the intended mechanism of action of the active ingredient	Critical characteristic of a bioassay to ensure specificity towards the targeted biological activity
	No interference from relevant process related impurities or matrix components	For example, process related and matrix components do not significantly affect the characteristics of the dose response curve
	Assay is stability indicating i.e., capable of detecting a change in potency and/or a change in the shape of the dose response curve, confirmed using forced degraded samples	To ensure that the product remains within specification over its shelf-life
Reportable range	The potency range is the range that meets accuracy and precision. It should include the specification range (80% to 120% of the specification range in this case corresponding to 64% to 150% for a specification of 80% to 125% relative potency[1)])	Stated range for which the required accuracy and precision characteristics are demonstrated

1) Individual values are just an example and can be different from product to product.

Technology Selection

Binding assays and cell-based bioassays are suitable technologies for the measurement of the relative potency of an anti-TNF-alpha monoclonal antibody when considering the ATP above. The two assays rely on the binding of the anti-TNF-alpha monoclonal antibody to the soluble TNF-alpha. While the signal of a binding assay directly measures the binding, the cell-based assay may target a later stage event in the signalling cascade. Out of different formats of cell-based assay, the cell-based proliferation assay was chosen as it is widely used and a well characterised cell line was available.

Analytical Procedure Development

The development of the analytical procedure described has been performed using an enhanced approach and was based on extensive knowledge of the molecule and relative potency assays, considering the following points for example:

- Performance characteristics and associated criteria for the analytical procedure defined in the ATP;
- Extensive analytical procedure understanding gained from prior knowledge and development studies guided by QRM principles. Factors considered during risk assessment are shown in Annex A, Figure 2: e.g.,
  - The cell and its performance (cell density, cell viability, number of passages);
  - Stability indicating properties using forced degradation samples;
- Robustness evaluation was conducted and its outcome was reflected in the analytical procedure control strategy.

Figure 2: Ishikawa diagram

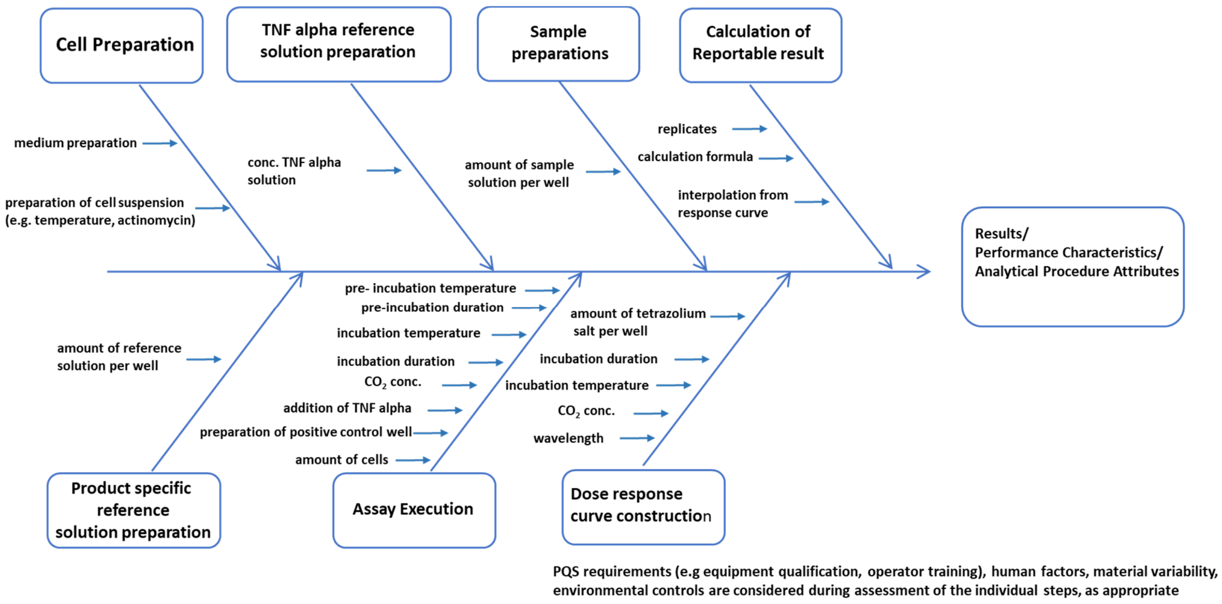

Analytical Procedure

For the purpose of this example, a summary of the analytical procedure is provided below. This does not reflect the entirety of the procedure description in the dossier.

Table 5 Analytical procedure description

Unit Operation	Description
Cell preparation	Prepare a suspension of WEHI-164 cells containing $1x10^6$ cells per millilitre, using assay medium containing 2 µg/mL of actinomycin D
Reference solution and test solution preparation	Included in analytical procedure description in the dossier but not listed in this table
Plate preparation	
Plating cells	
Absorbance measurement	
Calculations	
Solutions & reagents preparation	WEHI-164 cells (ATCC), TNF-alpha solution of suitable concentration, assay and culture medium including components and concentrations-Actinomycin D, Tetrazolium salt WST-8
**Analytical Procedure Control Strategy**	
System suitability test	1. The dose-response curve obtained for the reference standard curve corresponds to a sigmoid curve with upper and lower plateaus corresponding to 'cell only control' and 'cell + TNF-alpha control', respectively<br>2. The dose-response curve obtained for the test sample corresponds to a sigmoid curve with upper and lower plateaus corresponding to 'cell only control' and 'cell treated with TNF-alpha control', respectively.<br>3. The coefficient of determination calculated for each standard curve (r^2) is not less than 0.97 *<br>4. Maximum value (cell only) to minimum value (TNF-alpha control) ratio: minimum 3.0*
Sample suitability assessment	Assessment of similarity/parallelism:<br>- The upper asymptote ratio (A_{std}/A_{test}): 0.8–1.2*<br>- The lower asymptote ratio (D_{std}/D_{test}): 0.8–1.2*<br>- The Hill slope ratio (B_{std}/B_{test}): 0.8–1.2*<br>- The upper to lower asymptote ratio ($(D–A)_{std}/(D–A)_{test}$): 0.8–1.2*

* The ways of assessing of similarity/parallelism as well as individual values are just examples and can be different from product to product.

Analytical procedure validation

After the analytical procedure development was finalised and the analytical procedure control strategy established, a validation study was planned and completed according to the recommendations in ICH Q2.

Description of established conditions, reporting categories, and justifications

The applicant proposed and justified established conditions and reporting categories, as part of the submission. For the purpose of this example, Annex A Table 6 describes a portion of the proposed ECs, their proposed reporting and an example of a parameter that is not an EC.

Note: The extent of ECs and associated reporting categories listed in this table depend on the extent of knowledge gained, information and justification provided in the dossier. The dossier is subject to regulatory review. The information provided in this example is only part of the knowledge available that will be submitted and is provided for illustrative purposes only. The extent of ECs (EC or not EC designation), actual reporting categories, and data requirements may differ by region. Depending on the nature and extent of the change (e.g., change to a different technology), a PACMP may be required.

Table 6: Evaluated risk, proposed established conditions and proposed reporting categories

**Established condition**	**Overall Risk Category**	**Proposed Reporting Category[1)]**	**Comment**
Performance characteristics and associated criteria as defined in the ATP (Annex A Table 4)	High	PA	The performance characteristics and criteria ensure the quality of the reportable result and link to the CQA. Widening of performance characteristics and criteria could have an impact on the control of the CQA
Technology (principle)<br>Cell Based Assay	High or Medium	PA or NM	Adherence to performance characteristics and criteria ensured by control strategy and defined bridging strategy (see below) to assess impact of changes<br>Change would be reported as Notification Moderate if no impact of the change on the specification acceptance criteria and as Prior Approval if there is an impact on the specification acceptance criteria
Analytical procedure control strategy elements (SST 1-4, sample suitability assessment)			
System suitability test<br>(see Annex A Table 5)	Medium	NM[2)]	Performance of the analytical procedure is ensured by<br>• Direct control of individual analytical procedure steps through analytical procedure

Established condition	Overall Risk Category	Proposed Reporting Category[1)]	Comment
Sample suitability assessment<br><br>(see Annex A Table 5)	Medium	NM[2)]	control strategy elements listed in Annex A Table 5 (and the dossier)<br>• Defined analytical procedure control strategy elements which ensures the adherence to the ATP<br>• Adherence to the performance characteristics and criteria after a change of analytical procedure control strategy elements<br><br>If assurance of performance of the analytical procedure cannot be demonstrated, the change needs to be reported as Prior Approval
Cell Preparation			
Cell line:<br><br>WEHI-164 cells (ATCC)	Medium	NM	Based on demonstrated understanding of the mode of action (link to CQA) the suitability of the responsive cell line will be confirmed by responding to the TNF-alpha (survival of the cell in presence of the drug and cell death without drug)<br><br>Adherence to ATP ensured by control strategy and defined bridging strategy (see below) to assess impact of changes<br><br>System suitability test ensures the suitability of the cell line and its performance (number of passages, confluency, cell counting, cell viability, signal amplitude, shape of the response curve)
Preparation of cells: sub culturing	Low	NL	Sufficient cell performance to detect changes in the quality of the drug is ensured by:<br>• System suitability covers the suitability of the cell preparation (number of passages, confluency, cell counting, cell viability, signal amplitude, shape of the response curve)<br>• Changes in cell metabolism that impact performance of the analytical procedure and link to CQA will be detected
Medium composition:<br><br>RPMI 1640, L-glutamine, heat-inactivated foetal bovine serum, and a suitable antibiotic	Low	NL	

Established condition	Overall Risk Category	Proposed Reporting Category[1)]	Comment
Preparation of a suspension of WEHI-164 cells containing $1x10^6$ cells per millilitre, using assay medium containing 2 µg/mL of actinomycin D.	Low	NL	• Changes that lead to insufficient cell performance will not be implemented as they could have an impact on the defined performance characteristics and would require prior approval<br>Analytical procedure control strategy ensures adherence to performance characteristics and criteria. The extent of the bridging study will depend on the extent of the change
**The other analytical procedure parameters defined as ECs are omitted for the purpose of this example**			
**The following is an example of a parameter that is not an EC:**			
Plating format	Low	-	No impact on assay output based on development data

1) PA: Prior Approval, NM: Notification Moderate; NL: Notification Low (as per ICH Q12 definitions)

2) Based on regional requirements the proposed reporting category may need to be elevated to PA

Change assessment and bridging strategy

For every change, the applicant will perform a risk assessment to evaluate potential impact on the performance characteristics and the link to CQA (biological activity) as defined in the respective ATP. The outcome of the risk assessment informs the extent of the bridging studies used to demonstrate adherence to the performance characteristics and associated criteria. These can include, if necessary, full or partial revalidation of the analytical procedure performance characteristics affected by the change and/or comparative analysis of representative samples and reference material.

The applicant should not implement the new analytical procedure using the predefined reporting category unless adherence to the performance characteristics and associated criteria defined in the ATP are demonstrated during the bridging studies. If the precondition of adherence to the ATP cannot be met, a higher reporting category would apply.

The example in Annex A Figure 3 illustrates a post-approval change in the cell preparation from subculture to ready to use cells and includes the steps an applicant would follow when actually implementing the change.

The ECs and reporting categories (see Annex A Table 6) would need to be proposed following ICH Q14 Figure 2 and agreed up front with the regulatory authority.

Figure 3: Example of work process of applicant to change an approved analytical procedure

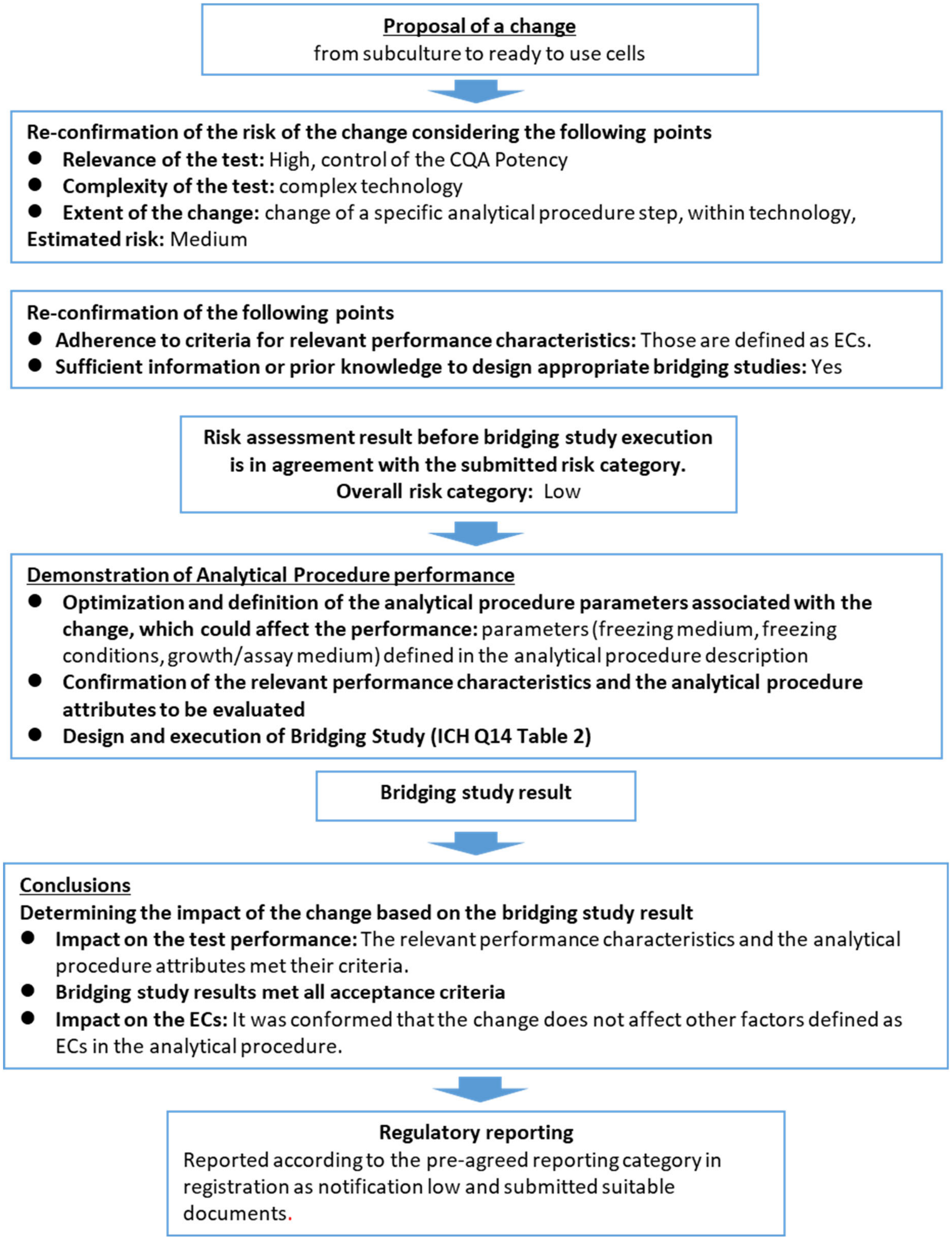

13.2. Annex B: Example of multivariate model lifecycle components

	Example 1	Example 2	Example 3
Model Description	On-line NIR to determine blending ranges to achieve blend uniformity during development	Measurement of Content Uniformity and Assay of uncoated tablets by NIR used for product release	Glucose Raman model used for qualitative identification testing on incoming raw material release for GMP use
	Model Category - Low Impact	Model Category - High Impact	Model Category - High impact
	User requirements	Defined model requirements (*e.g.,* ATP)	Defined model requirements (*e.g.,* ATP)
Risk Assessment	Initial assessment based on existing knowledge, laboratory and pilot studies, or DoE, as appropriate	Formal risk assessment based on knowledge gained during initial development	Formal risk assessment with knowledge gained during initial development
Model Development - Calibration	Scientifically sound approach based on laboratory and pilot data and previous experience	Formal design-based approach (*e.g.,* DoE) covering appropriate ranges of relevant variability sources with established acceptance criteria that are suitable for the intended purpose	Formal design-based approach covering appropriate ranges of relevant variability sources (raw material, lots, packaging, instrument-to-instrument, user, software limitation) with established acceptance criteria that are suitable for the intended purpose. Establish an identification threshold that has the same probability of detection as the existing analytical procedure and a suitable alternative analytical procedure should the Raman analytical procedure fail
Validation	Assess specificity and robustness, optionally assess linearity and/or precision	Full validation covering applicable performance characteristics across reportable ranges with established acceptance criteria (ICH Q2)	Full validation covering applicable performance characteristics across reportable ranges with established acceptance criteria (ICH Q2). Include establishing suitable comparability of Raman procedure to existing analytical procedure for release (can be reference analytical procedure)
Performance Monitoring	Routine monitoring - maintain data sources (instruments), automation connectivity, and data integrity	Routine monitoring - maintain data sources (instruments), automation connectivity, and data integrity	Routine monitoring - maintain data sources (instruments), automation connectivity, and data integrity
	Real-time diagnostics - implement initial diagnostics to confirm model performance in real-time	Real-time diagnostics - implement routine diagnostics to confirm model performance in real-time	Real-time diagnostics - implement routine diagnostics to confirm model performance in real-time

	Periodic monitoring - if applicable, compare model predicted results to reference analytical procedure at a frequency that is scientifically justified or on an event driven basis as needed	Periodic monitoring - compare model predicted results to reference analytical procedure at a frequency that is scientifically justified or on an event driven basis	Periodic monitoring - compare model predicted results to reference analytical procedure at a frequency that is scientifically justified or on an event driven basis
Model Maintenance	Model Update - updates are common during the process development stage as new experimental data becomes available	Model Update - updates should be triggered based on Model Monitoring and Maintenance Strategy	Model Update - updates should be triggered based on Model Monitoring and Maintenance Strategy
	Change Management per PQS	Change Management per PQS. with regulatory communication as required	Change Management per PQS, with regulatory communication as required
